# Development and application of core collection, KASP markers and SNP-based DNA fingerprinting in *Ziziphus jujuba*

**DOI:** 10.3389/fpls.2026.1773602

**Published:** 2026-04-23

**Authors:** Bingqi Shen, Yanni Chen, Kun Li, Juan Jin, Ye Yuan, Lili Li, Chong Chen, Dingyu Fan, Qing Hao, Lei Yang

**Affiliations:** 1 Institute of Fruit and Vegetable Research, Xinjiang Uygur Autonomous Region Academy of Agricultural Sciences, Key Laboratory of Genome Research and Genetic Improvement of Xinjiang Characteristic Fruits and Vegetables, Urumqi, China; 2 College of Urban and Rural Construction, Fuyang Institute of Technology, Fuyang, China

**Keywords:** core collection, germplasm fingerprinting, KASP marker, SNPs, *Ziziphus jujuba*

## Abstract

The nutritional and medicinal importance of jujube (*Ziziphus jujuba*) has spurred ongoing genomic research to enhance the effective utilization of its germplasm resources. However, the limited information about the genetic diversity and genetic relatedness of the existing germplasm resources has restricted the improvement of jujube germplasm. This study analyzed the resequencing data of 460 representative jujube germplasms and identified 585,131 high-quality SNPs. After strict screening, 500 candidate SNPs were obtained from these SNPs, and finally 100 core markers were selected for KASP marker development, with an average Shannon-Weiner index of 0.868 and a polymorphism information content (PIC) value of 0.412, indicating high informativeness and discriminatory power. Through the analysis of the minimum core markers and core germplasms, 92 core germplasm subsets were determined, and a fingerprint identification system covering all 460 jujube germplasms was constructed. Ten jujube germplasms were randomly selected from 460 jujube samples, and 40 core SNPs were verified by PCR and Sanger sequencing. Finally, 23 SNPs were successfully converted into KASP markers (conversion rate 57.5%). These 23 KASP markers were used for genotyping 96 samples (46 natural populations and 50 hybrid offspring), showing high discrimination and effectively distinguishing different populations and hybrid offspring. Genotyping the representative varieties with core markers and generating unique QR codes provide a strong technical support for resolving intellectual property disputes caused by homonymous varieties or varieties with different names but the same identity. This study provides essential genetic resources for the precise identification of jujube germplasm and substantially supports the conservation and use of genetic resources, varietal authentication, and marker-assisted breeding in *Z. jujuba*.

## 1 Introduction

Jujube (*Ziziphus jujuba* Mill.) is a prominent fruit tree in China that is valued for its nutritional and medicinal properties. It has extensive genetic resources and a long cultivation history. Jujube has generated abundant varietal diversity through prolonged natural evolution and targeted artificial selection. Hitherto, more than 800 jujube cultivars have been documented (Liu et al., 2020; Liu and Wang, 2009). Nevertheless, accurately classifying and managing jujube germplasm presents significant challenges. First, there is considerable confusion in variety nomenclature, with the same cultivar often known by multiple local names and different cultivars sharing similar names. For example, the high-quality fresh food variety *Z.jujuba* var. ‘Dongzao’ has more than 20 local names, complicating accurate variety identification (Wang et al., 2014). Second, the origins and genetic lineages of many cultivars remain unclear. Frequent germplasm exchanges among different production regions, primarily through asexual propagation methods such as grafting or cuttings, combined with the paucity of historical documentation, have hindered the reconstruction of the genetic background for most jujube cultivars (Song et al., 2021). Third, the classification of closely related species remains contentious. The genus *Ziziphus* comprises more than 200 species, and the genetic boundaries between cultivated jujubes, wild jujubes *(Z. jujuba* var. spinosa), Indian jujubes (*Z. mauritiana*), and other related taxa are poorly defined. Interspecies hybridization and gene introgression further complicate taxonomic delineation (Liu et al., 2020). Consequently, issues such as mixed varieties, ambiguous genetic background, and inadequate intellectual property protection have substantially constrained the effective utilization of jujube germplasm, cultivar rights, and molecular breeding initiatives.

The conventional morphological identification of jujube depends on phenotypic characteristics such as fruit shape and leaf morphology. These traits are highly influenced by environmental conditions and exhibit low polymorphism, limiting their utility for precise classification (Liu and Zhao, 2009). Various molecular markers, including Sequence-Related Amplified Polymorphism (SRAP), SNPs, and Simple sequence repeat (SSR), have been applied for the characterization and management of jujube germplasm (Fu et al., 2016; Bai, 2008; Xiao et al., 2015). Among these, SSR markers have become a cornerstone of jujube molecular research due to their co-dominant inheritance, high polymorphism, and stability (Xiao et al., 2015). Although previous studies have assessed the genetic integrity of 962 jujube germplasms, 448 germplasms (47.6% of the total) exhibited synonymous mislabeling, in which different names corresponded to identical SSR genotypes, resulting in a relatively high misidentification rate (Xu et al., 2016). Existing SSR markers are primarily used for small-scale population validation and lack systematic genome-wide screening. Furthermore, studies on population structure remain limited, core germplasm constructions are still in their infancy, and genetic research in jujube continues to lag behind the progress achieved in other fruit crops. The absence of segregating populations due to challenges in artificial hybridization (e.g., small flowers and low fruiting rate) and the limited development of functional markers are the two major bottlenecks.

DNA fingerprinting employs molecular markers or specific DNA sequences to assess genetic variation and distinguish between plant varieties (Iqbal et al., 2021). DNA fingerprinting databases have been established for several crops, including corn (Tian et al., 2015), grape (Wang et al., 2022), bottle gourd (Wang et al., 2021), and broccoli (Shen et al., 2021), highlighting its strong applicability in cultivar identification and germplasm innovation. The completion of the jujube reference genome has enabled the development of novel molecular markers over the past few years. SNP marker technology derived from whole-genome resequencing has been applied to construct high-density genetic linkage maps (such as a map comprising 3,678 SNPs) and facilitate quantitative trait locus (QTL) mapping, thereby advancing the understanding of complex traits in jujube (Liu et al., 2014). SNP markers have become a pivotal tool for resolving the intricate genetic background of jujube and its related taxa because of their broad genomic distribution, high polymorphism, genetic stability, and suitability for high-throughput detection. In applications such as genetic diversity analysis, variety identification, molecular breeding, and germplasm conservation, SNP markers have demonstrated substantial practical value (Sapkota et al., 2024). Song et al. (2021) identified 96 high-quality SNPs from 114 jujube germplasm accessions in Ningxia for marker development and genetic diversity studies. These markers enable the precise identification of jujube cultivars and provide a robust foundation for germplasm fingerprinting profiles, variety protection, seed purity testing, and efficient germplasm management. Despite these advancements, most of the existing studies are limited to specific regions or specific populations, lacking a nationwide coverage of major cultivated varieties and wild jujube resources. Secondly, the standardization of SNPs is low and the validation of the results is insufficient, which highlights the necessity of developing and validating the core SNPs.

In this study, a core collection of jujube varieties was constructed using high-quality SNPs generated from whole-genome resequencing of 460 jujube germplasms. Genetic diversity analysis was subsequently conducted to assess the representativeness of the selected core germplasms. SNP-based DNA fingerprinting profiles were developed to achieve precise distinction among germplasms. A set of 100 SNPs was ultimately selected to construct the DNA fingerprinting system. Furthermore, population genetic analysis and variety authentication of 96 representative accessions were performed based on KASP genotyping to validate the accuracy and robustness of the core SNP markers. Overall, these findings enhance the effective conservation and use of jujube germplasm resources while providing a solid scientific foundation and reference framework for continuous germplasm collection, management, and identification.

## 2 Materials and methods

### 2.1 Plant materials

A total of 460 whole-genome resequencing datasets representing jujube germplasm with diverse morphological traits were used to screen a core SNPs set (**Supplementary Table 1**), including publicly available datasets and sequencing data from 75 germplasms generated in this study (**Supplementary Table 1**) (Guo et al., 2020; Li et al., 2024; Zhang et al., 2022). KASP genotyping was subsequently performed on 96 accessions, comprising 46 natural populations and 50 hybrid lines of jujube (**Supplementary Table 2**).

### 2.2 SNP selection

Following quality control of the resequencing data, clean reads were aligned to the jujube reference genome (PRJCA049993) using Burrows-Wheeler Aligner (BWA v0.7.8, parameters: bwa mem -t 3 -M) (Li and Durbin, 2010). Duplicate reads were subsequently removed with Samtools (v1.15) (Li et al., 2009). SNP variant were calling was conducted in Genome Analysis Toolkit (GATK) (v4.2.0.0) (McKenna et al., 2010), generating Genomic Variant Call Format (GVCF) files for each accession. All individual GVCFs were integrated using CombineGVCFs, and genotyping of the merged variant file was conducted using GenotypeGVCFs to produce a comprehensive variant dataset. High-confidence SNPs were filtered using ANNOVAR to ensure the reliability of downstream analyses based on the following criteria: coverage depth > 4, missing rate < 10%, and minor allele frequency (MAF) > 0.5% (Wang et al., 2010). After excluding low-quality and low-discriminatory SNPs, the remaining high-quality SNPs were retained for the identification and selection of core SNPs across the 460 jujube germplasm resources.

### 2.3 Screening of the core germplasm collection

A representative core collection of jujube germplasm was constructed by evaluating 460 accessions using Core Hunter II software (De Beukelaer et al., 2012). A weighted multicriteria strategy was applied, incorporating modified Rogers’ distance (weight = 0.7) and Shannon’s diversity index (weight = 0.3), to identify core subsets representing 0.1, 0.2, 0.3, 0.4, 0.5, 0.6, 0.7, 0.8, and 0.9 of the total germplasm. The genic coverage (CV) of the candidate subsets was assessed using Core Hunter II (http://www.corehunter.org/). The final core collection size was determined by comparing allele coverage (CV) and multiple genetic parameters, including expected heterozygosity (He), Shannon-Weiner index, Nei’s gene diversity, and PIC, between the core subset and the complete germplasm set to ensure maximal representativeness and diversity retention.

### 2.4 Core SNP evaluation

Core SNP performance was evaluated using multiple population genetic analyses. Principal component analysis (PCA) was conducted with EIGENSOFT to characterize clustering patterns among accessions based on SNP genotypes (Price et al., 2006). Additionally, PCA was performed using GCTA software (v1.94.1, parameter: gcta64 --pca 3), and the resulting clusters were visualized in R using the ggplot2 package. A neighbor-joining phylogenetic tree was generated using distance matrices derived from SNP data in MEGA (v11.0.10) with 1000 bootstrap replicates (Kumar et al., 2018). The population structure and ancestry proportions were inferred using ADMIXTURE (v1.3.0, parameter: admixture --cv) (Alexander et al., 2009). Analyses were separately performed for the SNP dataset and the selected core SNP loci to verify the representativeness and robustness of the core markers. The number of ancestral clusters (K value) was set from 2 to 8, and the lowest cross-validation error was used to determine the optimal K value.

### 2.5 Fingerprint marker screening

A set of stringent selection criteria was applied to identify high-quality SNPs suitable for cultivar fingerprinting to ensure genomic representativeness, strong discriminative capacity, and high analytical reliability. Specifically, markers were required to: (1) exhibit uniform genomic distribution, high marker quality, and no missing loci; (2) exclude SNP loci with a MAF < 0.5%; (3) display a PIC > 0.4; (4) conform to Hardy-Weinberg equilibrium (*p* > 1×10^-6^); and (5) lack additional variants within 200 bp upstream and downstream of the target SNP loci satisfying these criteria were retained for subsequent fingerprinting analysis.

### 2.6 KASP marker design and genotyping

The 500-bp upstream and downstream flanking sequences surrounding each candidate SNP locus were extracted to develop the KASP marker. These sequences were aligned to the reference genome using BLASTN to remove nonspecific hits, and only unique sequences were retained for primer design. For each SNP target, two allele-specific primers and one common primer were generated. Primer design parameters were set as follows: (1) Guanine-Cytosine (GC) content < 60%; (2) melting temperature (Tm) between 55°C and 62 °C; and (3) PCR product size ≤ 120 bp. Sangon Biotech Co., Ltd. (Shanghai, China) synthesized the primers, including FAM and VIC fluorescent tails. The KASP primer design was conducted using Primer Premier V6.10. Genotyping reactions were performed in 384-well plates with a 5 μL reaction volume per sample. Fluorescence detection was performed using a BMG POLARstar Omega scanner, and genotype calling was performed using KlusterCaller v3.4.1. Detection data were visualized using the SNPviewer v2.0 software (LGC, Biosearch Technologies, Beverly, MA, USA).

### 2.7 Construction of DNA fingerprints pattern and two-dimensional barcode encoding

DNA fingerprint profiles were generated based on the selected SNP loci using RStudio (v4.4.0). Two-dimensional barcodes were produced using the online platform Caoliaoerweima (http://cli.im/) to facilitate efficient visualization and retrieval of genotype information for each variety. Each barcode enables direct scanning access to the corresponding genotype data for the associated jujube variety.

## 3 Results

### 3.1 SLAF-seq data analysis and SNP detection

Following quality control and filtering of sequencing data from 460 jujube germplasms, clean reads were aligned to the jujube reference genome, yielding an average sequencing depth of approximately 17.22×. The mean alignment rate reached 99.01%, with values ranging from 94.55% to 99.67%, thereby fully satisfying the requirements for downstream genomic analyses. Subsequent variant calling, genotyping, and filtering using GATK produced 20,708,799 high-quality SNP loci for further study. After a second round of filtering using ANNOVAR, 585,131 high-quality SNP loci were retained (**Table 1**). Functional annotation revealed that the majority of SNPs were located in the intergenic (363,691; 62.1%) and intronic (83,186; 14.2%) regions, whereas the exonic regions contained only 73,741 SNPs (12.6%). The overall transition-to-transversion (Ts/Tv) ratio was approximately 1.971, reflecting the predominance of synonymous mutations resulting from transitions.

**Table 1:** SNP detection and filtering results.

Category	Number of SNPs
Transitions (Ts)	388,212
Transversions (Tv)	196,919
Ts/Tv ratio	1.971
Total	585,131

### 3.2 Development of the core collection

**Figure 1:**
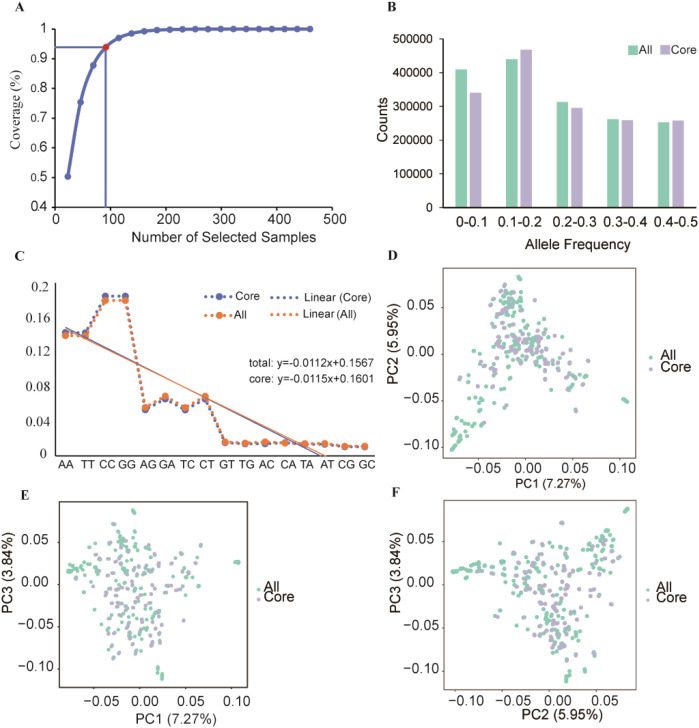
Screening and evaluation of the jujube core collection. **(A)** Allele coverage assessment across 460 jujube germplasm samples. The red dots indicate the 92 accessions selected for the core collection. **(B)** MAF distribution. The x-axis denotes MAF intervals, and the y-axis indicates the corresponding frequency. **(C)** Genotype frequency distribution for the full set of 460 jujube germplasm and the selected core set of 92 accessions. The x-axis shows the 10 possible genotypes, and the y-axis shows their proportional representation. The orange dashed line denotes the genotype distribution of the full germplasm set, whereas the blue dashed line indicates that of the core collection. The orange and blue solid lines show the corresponding fitted curves for the full germplasm and core set, respectively. The upper right corner of the figure presents a linear equation describing the fitted data. **(D–F)** PCA plots of the 460 germplasms and 92 core accessions. Each point represents an individual accession, with cyan and purple indicating all 460 jujube germplasm resources and 92 accessions included in the core germplasm set, respectively.

A core collection of 460 jujube germplasm accessions was established using 585,131 high-quality SNPs and Core Hunter software. The germplasms were comprehensively evaluated and screened based on a weighted combination of the modified Roger’s distance (weight = 0.7) and Shannon-Weiner diversity index (weight = 0.3). The assessment of gene coverage (CV) among the selected materials identified 92 accessions, representing 20% of the total collection, which were designated as the core germplasm. Genetic diversity analysis of these 92 core germplasms revealed observed heterozygosity (Ho) ranging from 0.000 to 1.000 (mean = 0.176) (**Figure 1A**, **Supplementary Table 3**), and expected heterozygosity (He) ranging from 0.103 to 0.500 (mean = 0.326) (**Table 2**). Nei’s diversity index varied from 0.348 to 0.448, while the average Shannon-Weiner index and PIC were 0.868 and 0.412, respectively. The evaluation of allele frequencies demonstrated that the ten possible genotypes derived from the four nucleotides (A, C, G, and T), including their dinucleotide combinations, were uniformly distributed across both the complete germplasm set and the 92 core accessions (**Figures 1B, C**). The comparison of MAF values between the core germplasm set and the entire collection revealed that the greatest proportion of MAF values fell within the 0.1–0.2 range, whereas the distribution of MAF values between 0.3 and 0.5 was consistent between the core and full germplasm sets. PCA of the 92 core germplasm sets demonstrated clustering patterns that were largely consistent with those observed in the complete set of 460 germplasms (**Figures 1D–F**), confirming that the core collection effectively captures the overall genetic diversity of the species.

**Table 2:** Genetic diversity parameters of the full and core jujube germplasm sets.

Germplasm set	Number of accessions	Observed heterozygosity (Ho)	Expected heterozygosity (He)	Nei’s diversity index	Shannon-Weiner index	PIC
All	460	0.363 (0.000-1.000)	0.312 (0.097-0.500)	0.402 (0.278-0.442)	0.854 (0.651-0.915)	0.402 (0.278-0.442)
Core	92	0.176 (0.000-1.000)	0.326 (0.103-0.500)	0.411 (0.348-0.448)	0.868 (0.769-0.924)	0.412 (0.348-0.448)

### 3.3 Core SNPs screening and DNA fingerprint construction

**Figure 2:**
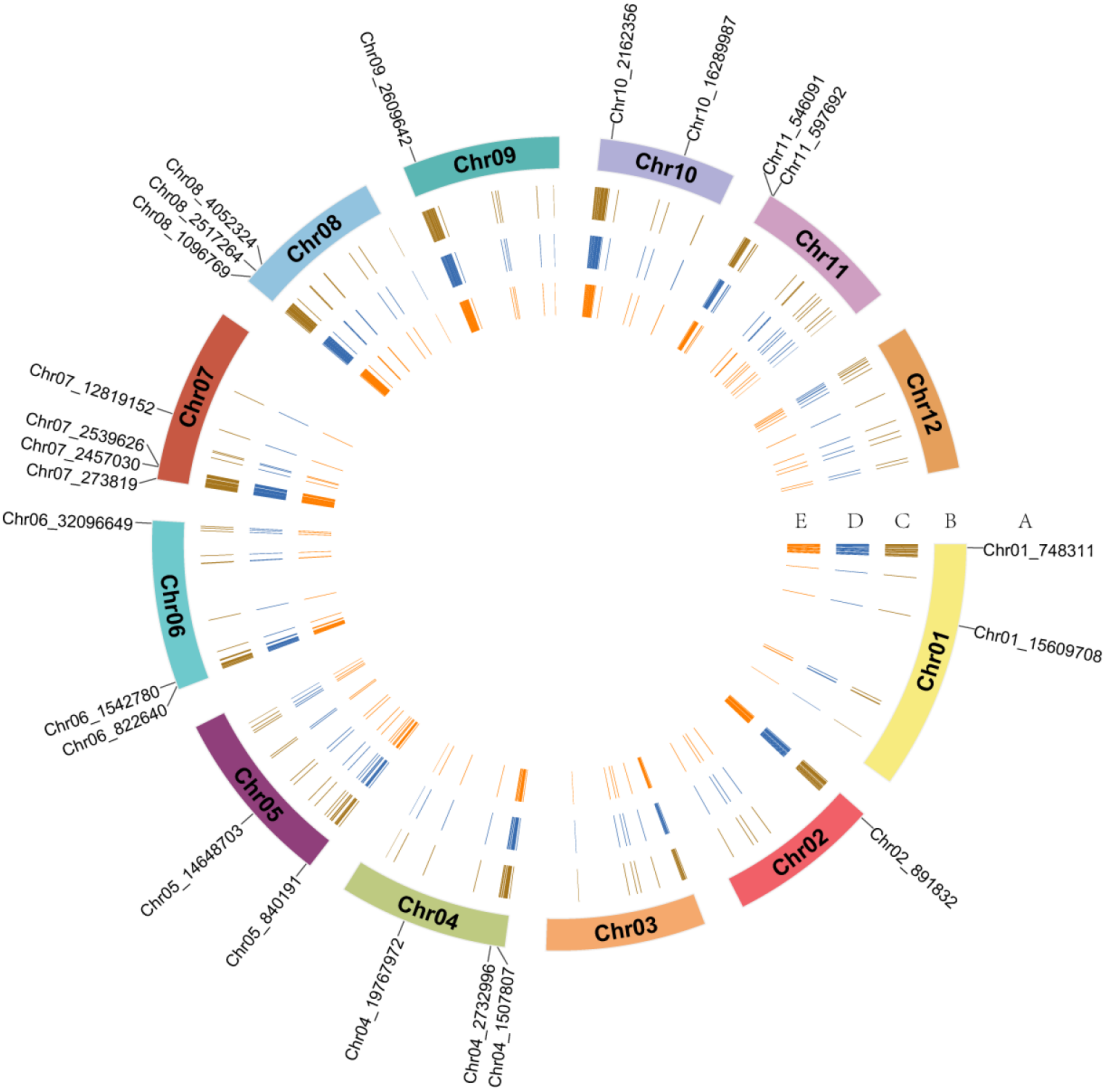
Genetic information map of the 500 high-quality SNP loci identified in this study. The map comprises four concentric circles, arranged from the outermost to the innermost: **(A)** names of the 23 KASP markers; **(B)** jujube chromosome numbers; **(C)** minor allele frequency (MAF); **(D)** polymorphism information content (PIC); **(E)** heterozygosity rate.

**Figure 3:**
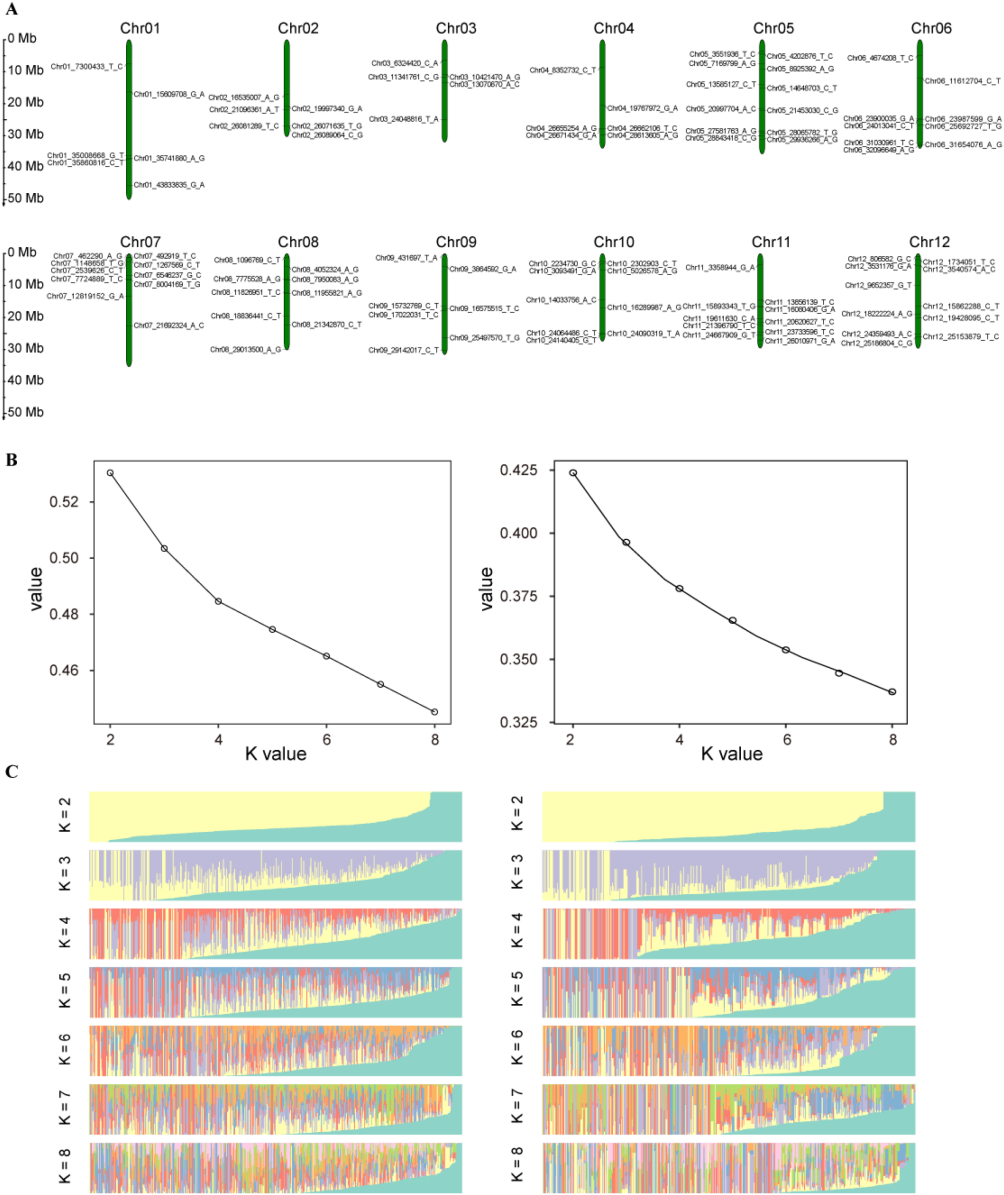
Analysis of the distribution and population structure of core SNPs. **(A)** Distribution of 100 core SNPs across 12 jujube chromosomes. **(B)** Delta K values for 460 jujube germplasm resources based on the core SNPs set (left) and using all SNP loci (right). **(C)** Population structure of 460 jujube germplasm resources inferred from the core SNPs set (left) and from all SNP loci (right) across different numbers of subgroups (K).

To identify suitable core SNPs, the germplasm resources were filtered using stringent criteria, including high marker quality, strong discriminatory power, broad genomic representation, uniform genomic distribution, and high specificity. A total of 500 variant loci were initially selected as candidate markers (**Supplementary Table 4**). These candidates were further refined by incorporating additional parameters, such as heterozygosity, deletion rate, MAF, and adjacent distance, to ensure an even distribution across the 12 chromosomes (**Figure 2**). Ultimately, 100 SNPs were uniformly distributed across the 12 chromosomes (**Figure 3A**). The discriminatory power of the final core SNPs was compared against the full SNP dataset used for population structure analysis in jujube germplasm to evaluate its effectiveness. STRUCTURE was used to conduct population structure analysis of 460 jujube germplasm resources. The cross-validation (CV) error rate remained similar across K values ranging from 2 to 8 (**Figure 3B**). At K = 2, the accessions were partitioned into two groups, whereas at K = 3, the samples could still be grouped into three, with mixed colors within each group indicating genetic interconnection and interpenetration (**Figure 3C**). Comparison of the population structure derived from all SNP sites with that obtained using the 100 selected core SNP markers revealed a high degree of similarity, demonstrating that the core SNP set had strong representativeness and discriminatory power. The PIC of the 100 core SNPs across the entire germplasm collection ranged from 0.405 to 0.577, with an average of 0.477, indicating sufficient polymorphism. The mean MAF and observed heterozygosity of these markers were 0.362 and 0.563, respectively (**Supplementary Table 5**).

**Figure 4:**
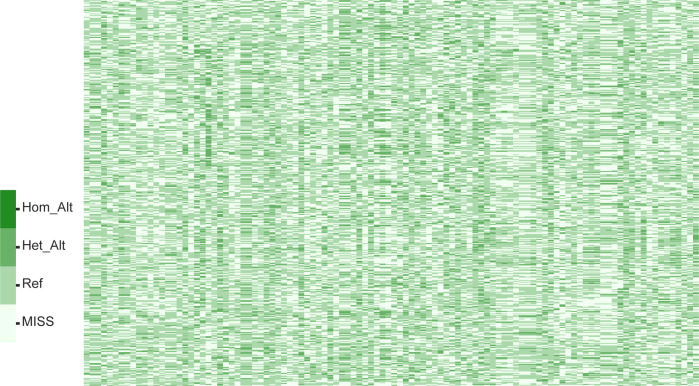
DNA fingerprinting map based on 100 core SNPs Each row in the matrix corresponds to an SNP locus, and each column represents a germplasm accession. The color gradient from light to dark indicates different genotypic states: MISS (missing loci), Ref (homozygous loci matching the reference genome), Het_Alt (heterozygous mutation loci), and Hom_Alt (homozygous mutation loci).

Genotyping data from 100 core SNPs across 460 accessions were integrated and analyzed in R to generate DNA fingerprints to enable rapid and accurate identification of jujube varieties. Each row in the resulting fingerprint matrix corresponds to an individual SNPs, and each column represents a germplasm accession (**Figure 4**). The online platform Caoliaoerweima (http://cli.im/) was used to encode the genotype data of 92 core jujube germplasms and generate two-dimensional (2D) barcode fingerprints for each variety (**Supplementary Figure 1**), which include information such as variety name and genotype data. These 2D barcodes can be scanned using a mobile device to directly access the corresponding genetic information. For instance, entry S14 in **Supplementary Figure 1** contains the following details: Name: Akesuxiaozao; fingerprint code: C/T G/A T/A T/T G/A A/A T/T A/A T/T G/G C/C G/G A/A C/C A/G A/A A/A C/C A/A A/A G/T A/A T/C C/T T/C T/T C/T A/G C/C G/T G/G G/A A/G G/A C/T C/A C/T A/G T/C G/G C/C G/G C/C A/G A/A T/T C/T C/C T/T C/G T/G G/A T/C C/C G/G C/T A/A A/A A/A T/A C/A G/A G/G T/C A/G G/G C/C C/C T/T A/A A/G C/C C/C A/A T/C C/A C/C G/G C/C T/T T/T G/G G/A A/A C/T A/C A/A A/G G/T G/A T/G A/G C/C G/G C/C G/T T/C A/G T/C T/A.

### 3.4 KASP primer design and validation of jujube germplasm resources

For marker validation, 10 jujube accessions (SaimisuNo.1, Chenguang, Zaocuiwang, Dongzao, Lelingwuhexiaozao, Linyilizao, Dalifengmiguan, Fucuimi, Huizao, and Junzao) were randomly selected from the 460 samples. Analytical accuracy was initially assessed using PCR and Sanger sequencing of 40 core SNP loci. A 500-bp flanking sequence was added upstream and downstream of each target SNP for the design and development of KASP primers to further verify the suitability of the candidate markers (**Supplementary Table 6**). Finally, 23 of the 40 core SNPs were successfully converted into functional KASP markers through first-generation sequencing validation (**Table 3**, **Supplementary Table 7**).

**Table 3:** KASP primer names, chromosomal locations, genomic positions, variant types, and primer sequences corresponding to the 23 identified KASP markers.

Marker	Chromosome	Marker	Allele X/Y	Primer sequences
SNP1	Chr01	748311	A/G	F1: GAAGGTGACCAAGTTCATGCTCTACAGTACCCTCTCCTGCATCTTATA F2: GAAGGTCGGAGTCAACGGATTCAGTACCCTCTCCTGCATCTTATG R: ATTCCTACAATCATCTCCATATGCA
SNP2	Chr02	891832	T/A	F1: GAAGGTGACCAAGTTCATGCTCCTCAACGTGATGCTTGGATAT F2: GAAGGTCGGAGTCAACGGATTCCTCAACGTGATGCTTGGATAA R: CCAAGAGATRTCATCGCTCCTTA
SNP3	Chr04	1507807	C/T	F1: GAAGGTGACCAAGTTCATGCTTTCAACCCCAAAATCACTTCAC F2: GAAGGTCGGAGTCAACGGATTTTCAACCCCAAAATCACTTCAT R: GATTGGGGTGAAATTCACAGAC
SNP4	Chr05	840191	C/A	F1: GAAGGTGACCAAGTTCATGCTGCCATCTCCCTGCTGAGC F2: GAAGGTCGGAGTCAACGGATTTGCCATCTCCCTGCTGAGA R: TTAAAGATGTTGTAATGTGGAGTTCC
SNP5	Chr06	822640	C/T	F1: GAAGGTGACCAAGTTCATGCTGAAAATTTTTGAACATGAAGACATATC F2: GAAGGTCGGAGTCAACGGATTGAAAATTTTTGAACATGAAGACATATT R: AAATTCAAAATTTCTCTTCGTGC
SNP6	Chr06	1542780	C/G	F1: GAAGGTGACCAAGTTCATGCTGACAAACAGTCCAGAGGTAGCC F2: GAAGGTCGGAGTCAACGGATTGACAAACAGTCCAGAGGTAGCG R: GTTAACAAACCAGCACACATGG
SNP7	Chr07	273819	G/A	F1: GAAGGTGACCAAGTTCATGCTGTCGAATAGGTCAGCAATTAAGG F2: GAAGGTCGGAGTCAACGGATTGGTCGAATAGGTCAGCAATTAAGA R: AAGGTCAGAGTCCGGAACAAA
SNP8	Chr07	2457030	G/A	F1: GAAGGTGACCAAGTTCATGCTCAAACGACCTCTCTTTGGCTG F2: GAAGGTCGGAGTCAACGGATTTCAAACGACCTCTCTTTGGCTA R: AGTGAAGTTGAATCTTAAACAACGC
SNP9	Chr08	4052324	A/G	F1: GAAGGTGACCAAGTTCATGCTAATCTGTCTTGACTGGCGACA F2: GAAGGTCGGAGTCAACGGATTATCTGTCTTGACTGGCGACG R: ACATCTCCTGTCCGAACGATC
SNP10	Chr09	2609642	G/A	F1: GAAGGTGACCAAGTTCATGCTTTCTGCCGAGCAAATATCG F2: GAAGGTCGGAGTCAACGGATTTTTCTGCCGAGCAAATATCA R: AAACACTTCTGAATCTCACGGAC
SNP11	Chr10	16289987	A/G	F1: GAAGGTGACCAAGTTCATGCTATGCATAAATGTGGTCATTGGAA F2: GAAGGTCGGAGTCAACGGATTATGCATAAATGTGGTCATTGGAG R: AGTAGTGCCAATTTCTCCTTTTTAA
SNP12	Chr11	546091	A/T	F1: GAAGGTGACCAAGTTCATGCTCCTTTGAATTTGGTGTACCATTGA F2: GAAGGTCGGAGTCAACGGATTCCTTTGAATTTGGTGTACCATTGT R: GGTTCAAATGGCAACTTTTCACT
SNP13	Chr11	97692	G/A	F1: GAAGGTGACCAAGTTCATGCTAGGAGCAAACAATGGCGAG F2: GAAGGTCGGAGTCAACGGATTAGGAGCAAACAATGGCGAA R: TCGATCAGGAGATACWGCAACT
SNP14	Chr04	2732996	A/C	F1: GAAGGTGACCAAGTTCATGCTCAGAGCGTTTTTATGCTTCCA F2: GAAGGTCGGAGTCAACGGATTCAGAGCGTTTTTATGCTTCCC R: GCATGCCAGTAAGCTCTATGAAT
SNP15	Chr08	2517264	C/T	F1: GAAGGTGACCAAGTTCATGCTCCAGTATAAAACTTAGGCTACCACTG F2: GAAGGTCGGAGTCAACGGATTGCCAGTATAAAACTTAGGCTACCACTA R: GCTACGCATATGGTCAGCCTAG
SNP16	Chr10	2162356	A/G	F1: GAAGGTGACCAAGTTCATGCTGGGAAAAGTGCATCAAGAACA F2: GAAGGTCGGAGTCAACGGATTGGGAAAAGTGCATCAAGAACG R: AGAATTGTTTATTACTAATGACTTTATCCTA
SNP17	Chr01	15609708	G/A	F1: GAAGGTGACCAAGTTCATGCTGCAATCAGTTTTTATCAACTACCTAAG F2: GAAGGTCGGAGTCAACGGATTGCAATCAGTTTTTATCAACTACCTAAA R: ACCTGAAGAGTTTGCTATTGGG
SNP18	Chr04	19767972	A/G	F1: GAAGGTGACCAAGTTCATGCTGCATTTGTTGTTGGATACTTGAA F2: GAAGGTCGGAGTCAACGGATTGCATTTGTTGTTGGATACTTGAG R: CAAACCTCTTTGGGAAGTTCAT
SNP19	Chr05	14648703	T/C	F1: GAAGGTGACCAAGTTCATGCTCCTCCACCTTATATATACATCGGACT F2: GAAGGTCGGAGTCAACGGATTCTCCACCTTATATATACATCGGACC R: GTTGTAATGGAGATAATGCTGCTTC
SNP20	Chr06	32096649	A/G	F1: GAAGGTGACCAAGTTCATGCTGTCCCCTGACCAATTGTTAATTA F2: GAAGGTCGGAGTCAACGGATTCCCCTGACCAATTGTTAATTG R: TTACAAGTAAGATTTAAATCAGTATTTTTAAT
SNP21	Chr07	2539626	T/C	F1: GAAGGTGACCAAGTTCATGCTCACCAAAATTTGGAGGGGTTA F2: GAAGGTCGGAGTCAACGGATTACCAAAATTTGGAGGGGTTG R: GGAAACCAACTTGAAAACCTGAC
SNP22	Chr07	12819152	G/A	F1: GAAGGTGACCAAGTTCATGCTGCCGCTATTAAAAGGCTACTG F2: GAAGGTCGGAGTCAACGGATTGGCCGCTATTAAAAGGCTACTA R: GCCTATAAGATAGTGTGCCATTCA
SNP23	Chr08	1096769	T/C	F1: GAAGGTGACCAAGTTCATGCTGGAAGAAAGAAGCCACTCCTACTT F2: GAAGGTCGGAGTCAACGGATTGGAAGAAAGAAGCCACTCCTACTC R: TTAACTTCCTTAACGACACTGGTAGA

**Figure 5:**
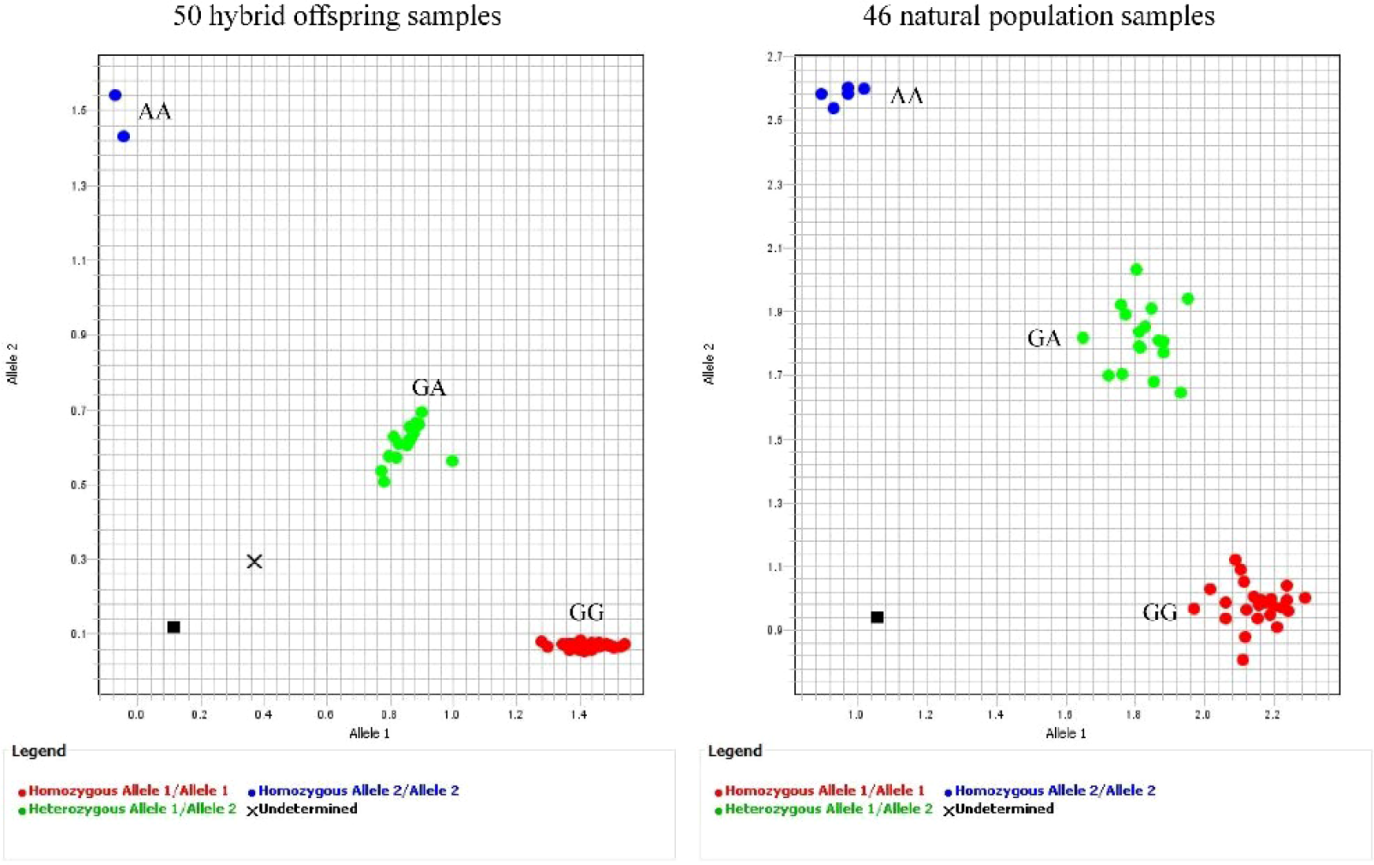
Representative results of fluorescence-based KASP genotyping using SNP17 for 46 natural population accessions and 50 hybrid offspring (Chr01_15609708).

To assess the effectiveness of the 23 KASP markers in distinguishing jujube varieties, 46 natural populations were chosen from 460 accessions, along with 8 hybrid offspring of ‘Huizao × Junzao’ and 42 hybrid offspring of ‘Fengtaixiaolingzao × Lengbaiyu’, for a total of 96 varieties analyzed via KASP (**Supplementary Table 2**). Genetic typing results demonstrated that all 23 KASP markers exhibited high discriminatory power, generating distinct genotyping profiles across the 46 natural populations and 50 hybrid offspring (**Figure 5**; **Supplementary Figure 2**; **Supplementary Table 8**).

Additionally, representative cultivars from the core collection, each associated with a unique QR code, were genotyped using the 23 KASP markers (**Table 4**), highlighting the potential of these markers to support the resolution of intellectual property disputes arising from the use of identical names for different varieties or multiple names for the same variety.

**Table 4:** Photographs, descriptions, and core SNP fingerprint information for representative cultivars.

Cultivar	Photos	Descriptions	Core SNP fingerprint	QR code
Zhenpingtailihong	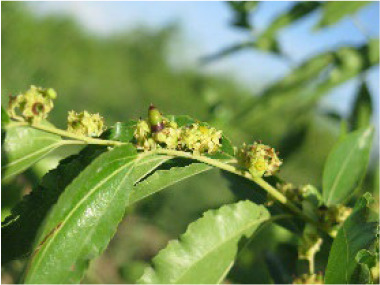	Flowers and young fruits are purple-red in color. As the fruits mature, the peel transitions from purple-red to pink and eventually to bright red, resulting in a highly ornamental appearance.	GTGTTGGGGTTAAAATGTGTTGCGAGCGTTGCATGAGCAC	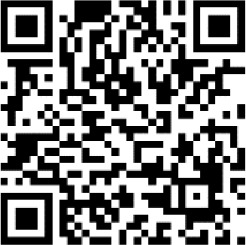
Shandonglizao	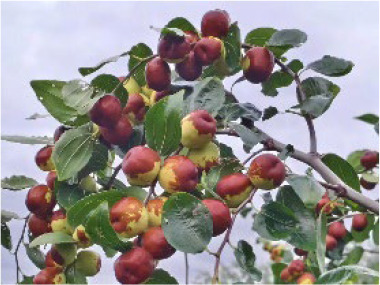	Late-maturing cultivar used primarily for fresh consumption, characterized by early bearing, exceptionally high yield potential, and superior quality of large fruits.	TTTATTCCTCAACGTGATGCTTGGATAATTGAATAAGGAG	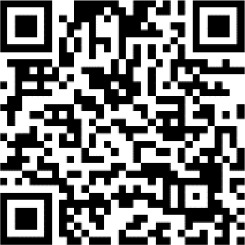
Dayewuhe	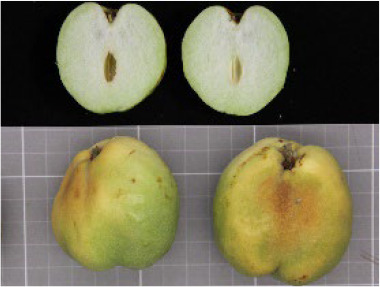	Extremely large, dark green leaves and undeveloped seeds are present. Seedless jujube is a rare germplasm with distinct and valuable traits.	AGAAAACTTCAACCCCAAAATCACTTCATAAGTCTGTGAA	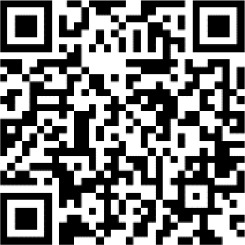
Jingcang No.1	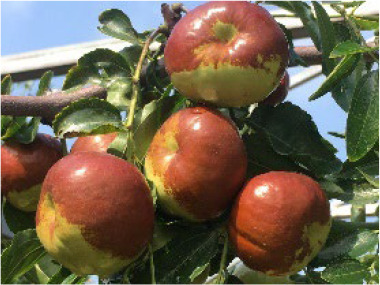	Late-maturing cultivar with strong self-pollination ability. The fruits are oblong and well suited for fresh consumption.	CCAACTACATATGATTGATAACCACATTCGATCCATTTGG	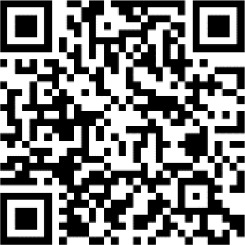
Shanxihuluzao	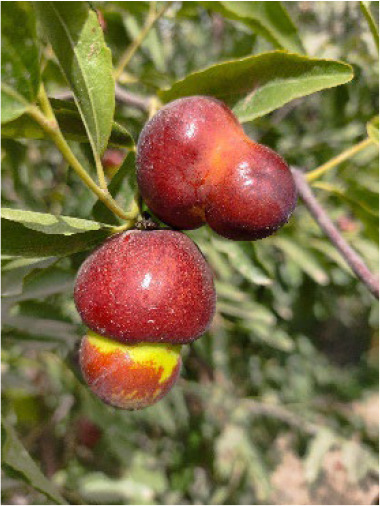	Large, gourd-shaped fruits with notable ornamental value are produced. Suitable for both fresh consumption and display purposes.	AACAGAGTTTGGTTTAGTTCCCTCTGCATGCATCTGTTTG	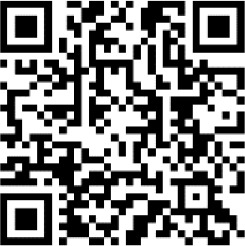
Saimisu No.1	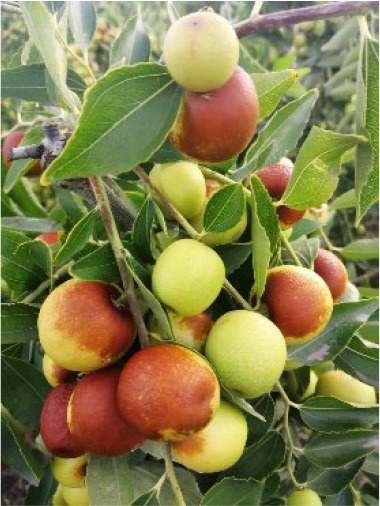	An early-maturing cultivar with high productivity, round fruit morphology, and high soluble sugar content.	CGAAAATTACGTTTACTACCATTTGCTCTTCCAATGCAAA	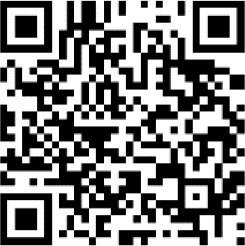
Jiancuizao	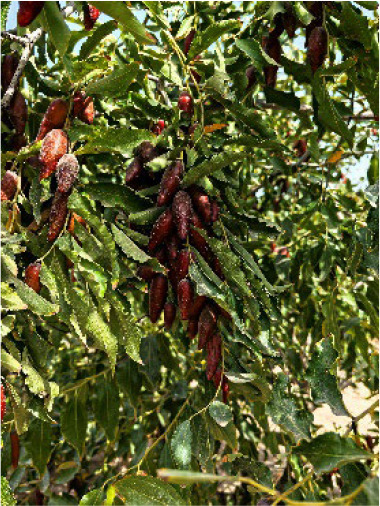	An early-maturing, medium-sized fresh fruit cultivar with high yield potential. Fruits exhibit an elongated, bullet-like shape.	GACAAACAGTCCAGAGGTAGCCGCCGTACCATGTGTGCTG	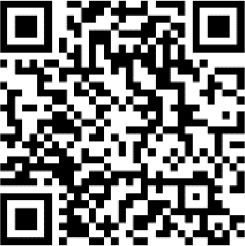
Jinmangguozao	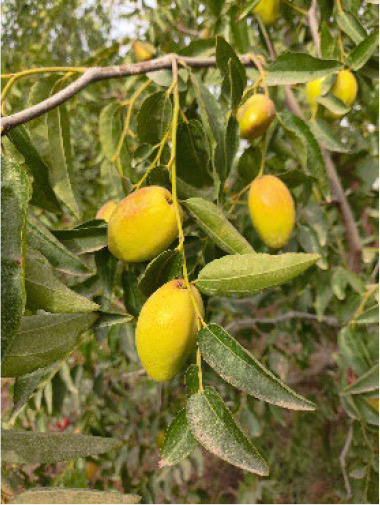	Late-maturing fresh fruit variety with robust yield performance. The fruits are morphologically similar to mangoes.	TCTAACTTGGTCAAATATTTTGCCCCACTAAGTTGGTCGA	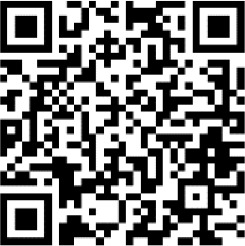
Suanzao No.1	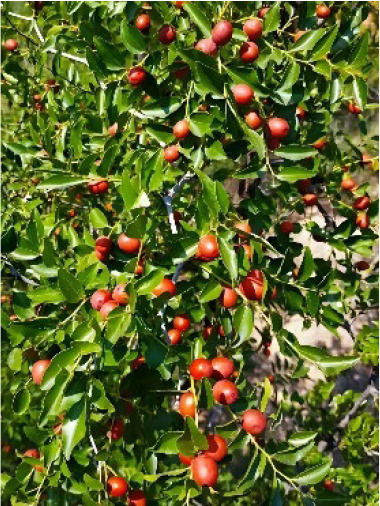	Wild jujube is the progenitor of domesticated jujube, which is widely used as a rootstock. It is known for its high nutritional value, medicinal importance, and strong drought and freezing stress tolerance.	TCTGCCTTTTGTTTTGCTTTCGAAAAACAGAAAAATCGGC	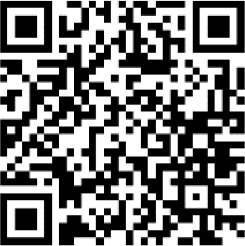

## 4 Discussion

Establishing DNA fingerprinting systems is critical for the rapid and precise identification of cultivar authenticity and genetic purity, thereby effectively protecting breeders’ rights and interests (Li et al., 2020). With the integration of high-throughput genotyping technologies, large-scale sample processing can be conducted efficiently, generating reliable and reproducible data. SNP markers have been extensively used in population structure analyses of numerous crop species, including cauliflower (*Brassica oleracea* var. *botrytis*) (Yang et al., 2022), bottle gourd (*Lagenaria siceraria*) (Wang et al., 2021), coffee (*Coffea canephora* L.) (Akpertey et al., 2021), and grape (*Vitis* spp.) (Bianchi et al., 2020). Whole-genome resequencing of 460 jujube varieties was performed, and 585,131 high-quality SNPs were identified via stringent filtering procedures, supported by an average sequencing depth of 17.22× and an alignment rate of 99.01%, ensuring high-confidence variant detection. The genomic distribution of SNPs was uneven, with the intergenic regions containing the largest proportion of variants (363,691; 62.1%), followed by intronic regions (83,186; 14.2%). In contrast, only 73,741 SNPs (12.6%) were located within the exonic regions. This distribution pattern reflects the evolutionary conservation of coding regions. The transition-to-transversion ratio was approximately 1.971, consistent with synonymous substitution enrichment, indicating that natural selection influences nucleotide substitution dynamics. These SNPs were distributed across all 12 jujube chromosomes and included numerous functionally relevant variants (e.g., 1,806 variants located in termination codons), providing a robust foundation for downstream genetic analyses and molecular marker development. A core collection of 92 germplasm accessions (representing approximately 20% of the total resources) was identified using Core Hunter and a complete set of 585,131 SNPs. The genetic diversity metrics of the core set (mean He = 0.326, PIC = 0.412) did not differ significantly from those of the full population, demonstrating that the reduced panel effectively retained the overall diversity of the entire collection. The distributions of allele frequencies, MAF values (predominantly falling within the 0.30-0.50 range), and PCA results consistently demonstrated that the core germplasms effectively represent the overall genetic diversity of the entire collection (**Figures 1C, D**). This approach addressed the challenge posed by the large number of conserved jujube germplasms and associated maintenance costs while establishing a streamlined and representative accession panel for the discovery of elite alleles and use in molecular breeding programs. The observed heterozygosity in the core germplasms (Ho = 0.176) was substantially lower than the expected heterozygosity (He = 0.326), indicating a pronounced level of inbreeding among cultivated jujube varieties. This finding underscores the need to enhance parental selection strategies in breeding to mitigate genetic erosion.

KASP technology enables efficient, flexible, accurate, and cost-effective genotyping, particularly when analyzing diverse samples using a limited number of SNP loci (Semagn et al., 2014). SNP-based fingerprinting systems developed from KASP markers have been successfully applied in multiple crops, including wheat (Rasheed et al., 2016), rice (Chen et al., 2014), cotton (Grewal et al., 2020), cabbage (Li et al., 2020), and cucumber (Zhang et al., 2020), thereby facilitating rapid and accurate varietal identification. Conventional marker systems, such as AFLP and SSR, have previously been employed for the genetic characterization of cauliflower and inbred and hybrid line fingerprinting (Fu et al., 2016). More recently, using SNP marker technology, Sapkota et al. (2024) revealed that significant morphological variations exist among American jujube accessions that share identical genetic backgrounds. This discrepancy implies that asexual propagation through somatic mutations may cause phenotypic divergence. Despite progress in molecular genotyping, an SNP-based fingerprinting system for jujube has not yet been established, highlighting the urgent need for a reliable and efficient platform to support species authentication, purity assessment, and population genetic studies. In this study, 585,131 high-quality SNPs were identified from whole-genome resequencing data, and computational simulation was applied to select an optimal SNP subset for DNA fingerprinting development. A fingerprint database consisting of 100 core SNPs was constructed, and these SNPs were subsequently converted into KASP markers to facilitate practical application. Among 40 randomly selected markers tested through KASP genotyping, 23 were successfully converted into KASP markers, enabling cost-effective and high-throughput discrimination of 100 jujube germplasms, including natural populations and hybrid descendants. This method is highly useful for rapid varietal identification and seed purity verification. The conversion rate (57.5%) was primarily affected by repetitive sequences or paralogous regions at certain loci, which reduced primer specificity. This limitation may be mitigated through optimized primer design or by selecting more conserved flanking regions for marker development. Despite the limited number of 23 KASP markers, the panel effectively differentiates natural populations and hybrid progenies across 100 germplasm resources, demonstrating its applicability for species identification and hybrid purity assessment. Moreover, the constructed fingerprint database (**Table 4**) facilitates digital management through two-dimensional barcoding, thereby offering robust technical support for jujube variety protection and seed market regulation.

## 5 Conclusion

In conclusion, this study developed a comprehensive genetic resource platform for jujube (*Ziziphus jujuba*) by integrating high-throughput SNP discovery, KASP genotyping, and whole-genome resequencing technologies. From 460 representative germplasms, we identified 585,131 high-quality SNPs, established a core collection of 92 accessions, and constructed a DNA fingerprinting system covering all 460 genotypes. Concurrently, 100 core KASP markers were developed, with 40 core SNPs validated via PCR and Sanger sequencing. Among these, 23 SNPs were successfully converted into functional KASP markers, which exhibited robust discriminatory power in distinguishing 46 natural population samples and 50 hybrid progenies. Collectively, our findings offer core genetic tools for precise jujube germplasm management, genetic diversity assessment, and marker-assisted breeding, ultimately facilitating the conservation and sustainable utilization of jujube genetic resources.

## Data Availability

The datasets presented in this study can be found in online repositories. The names of the repository/repositories and accession number(s) can be found in the article/**Supplementary Material**.

## Supplementary material

Supplementary Figure 1 The two-dimensional barcode fingerprints of 92 core jujube germplasms.

Supplementary Figure 2 The representative KASP-labelled fluorescence assay results of 46 natural population samples and 50 hybrid offspring samples.

Supplementary Table 1 List of 460 jujube accessions used in this study.

Supplementary Table 2 List of KASP genotyping population material used in this study.

Supplementary Table 3 List of 92 core germplasms assessment in this study.

Supplementary Table 4 List of 500 candidate SNP markers in this study.

Supplementary Table 5 List of 100 core SNP markers in this study.

Supplementary Table 6 List of 40 core SNPs validated through Sanger sequencing in this study.

Supplementary Table 7 List of PCR amplification results of 40 core SNPs in 10 samples in this study.

Supplementary Table 8 List of genotyping of 23 KASP markers primers in 96 samples in this study.
