# Supplementary material for: Development and application of core collection, KASP markers and SNP-based DNA fingerprinting in Ziziphus jujuba
Source: Front Plant Sci. 2026 Apr 23;17:1773602. doi: 10.3389/fpls.2026.1773602 (PMC13150858; doi:10.3389/fpls.2026.1773602)
Supplement: Supplementary Figure 1 — The two-dimensional barcode fingerprints of 92 core jujube germplasms. [file DataSheet1.docx]

**Development and Application of Core Collection, KASP Markers and SNP-Based DNA Fingerprinting in *Ziziphus jujuba***

Bingqi Shen ^a†^, Yanni Chen ^a†^, Kun Li ^b^, Juan Jin ^a^, Ye Yuan ^a^, Lili Li ^a^, Chong Chen ^a^, Dingyu Fan ^a^, Qing Hao ^a^*, Lei Yang ^a^*

^a^ Institute of Fruit and Vegetable Research，Xinjiang Uygur Autonomous Region Academy of Agricultural Sciences, Key Laboratory of Genome Research and Genetic Improvement of Xinjiang Characteristic Fruits and Vegetables, Urumqi 830091, China

^b^ College of Urban and Rural Construction, Fuyang Institute of Technology, Fuyang 236041, China

*Corresponding Authors: Qing Hao (haoqingxj@sohu.com); Lei Yang (yanglei9961@163.com)

† These authors contributed equally to this article.

**Supplemental data**

**Supplemental Data1.** The two-dimensional barcode fingerprints of 92 core jujube germplasms

**Supplemental Data2.** The representative KASP-labelled fluorescence assay results of 46 natural population samples and 50 hybrid offspring samples

| 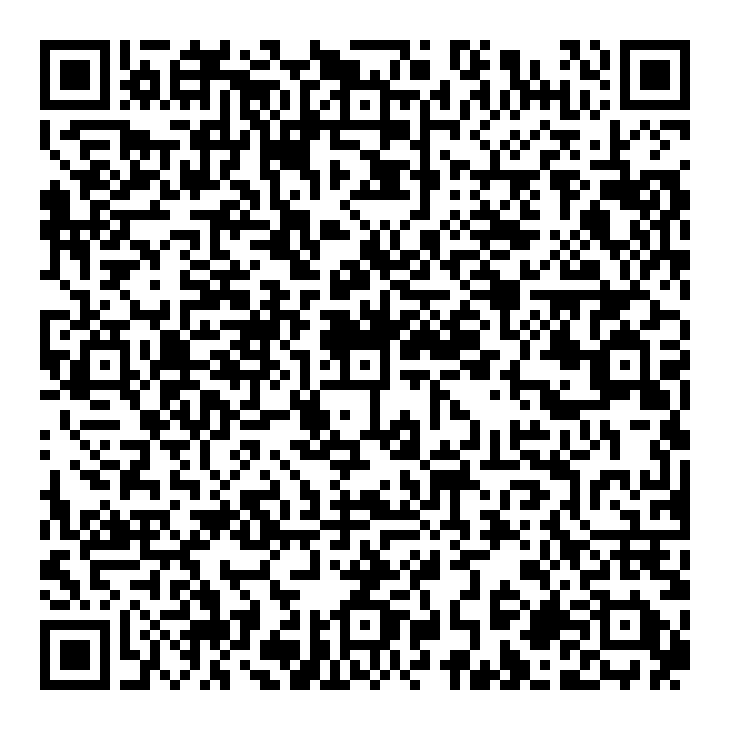C056 | 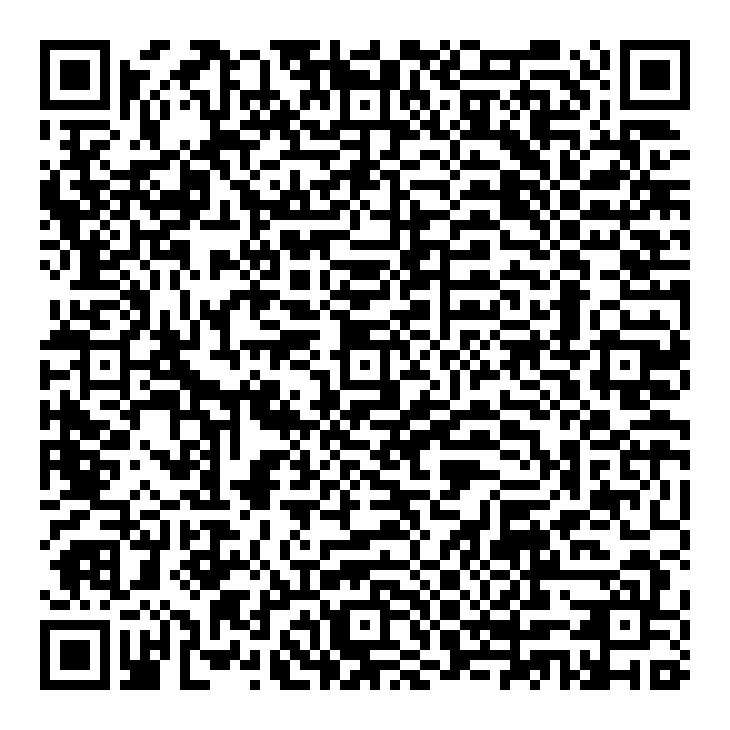C071 | 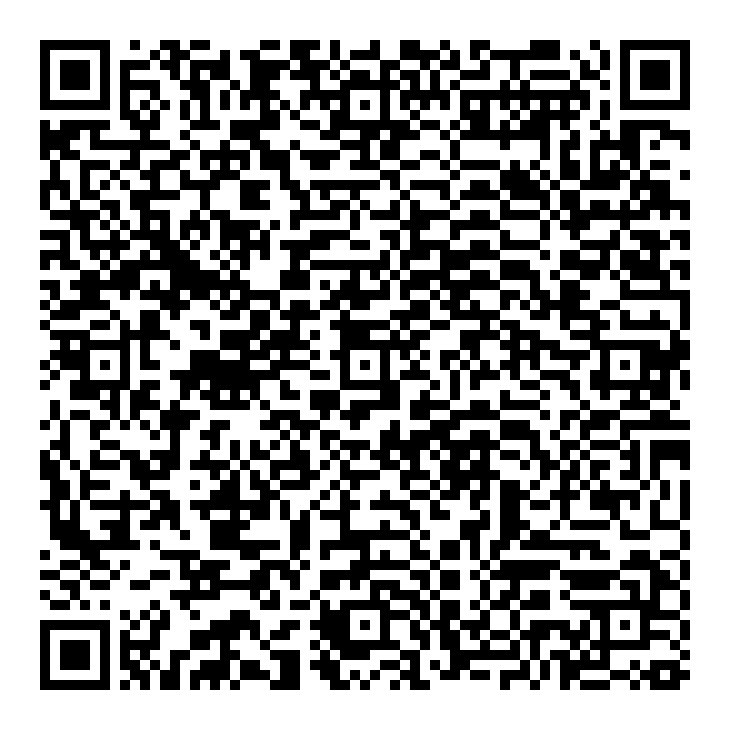C074 | 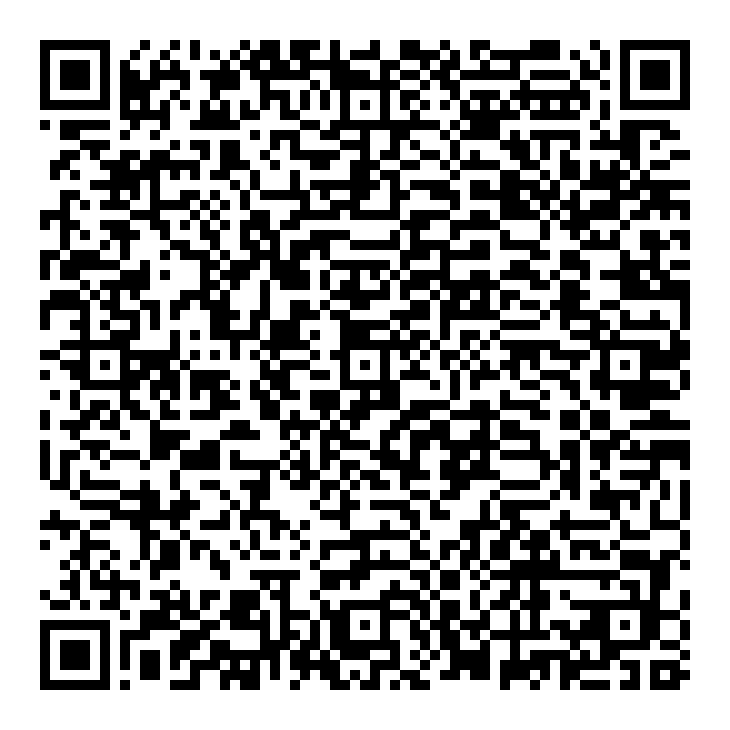C084 |
| --- | --- | --- | --- |
| 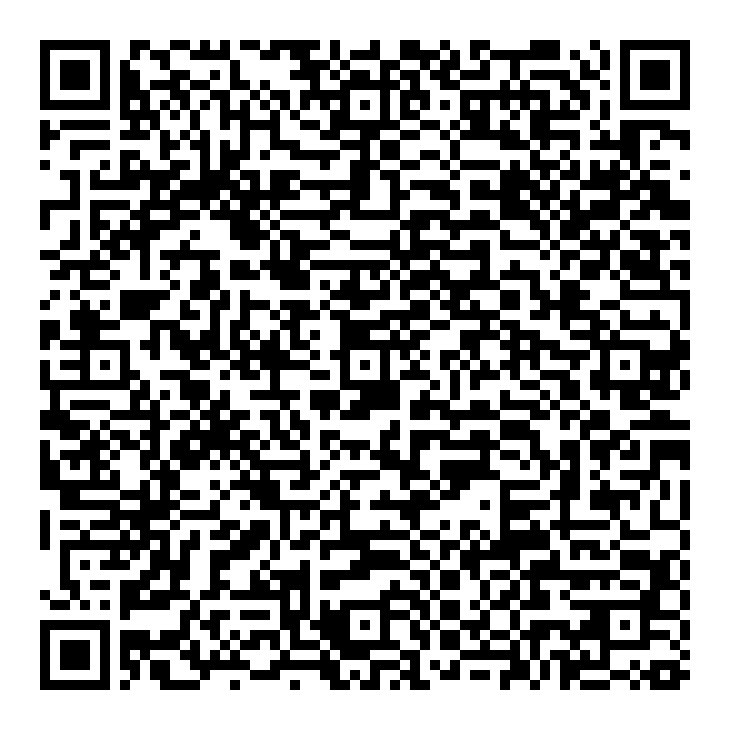C085 | 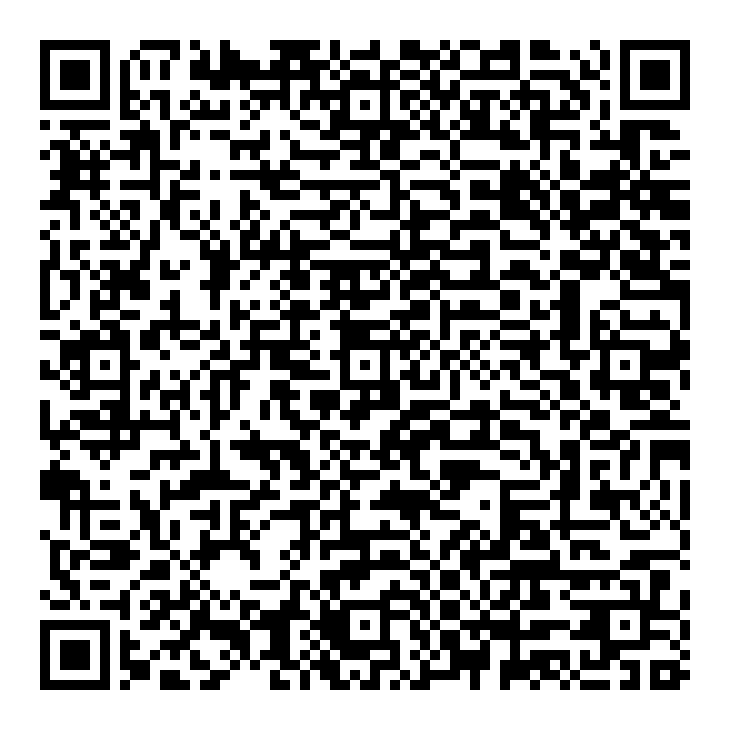C090 | 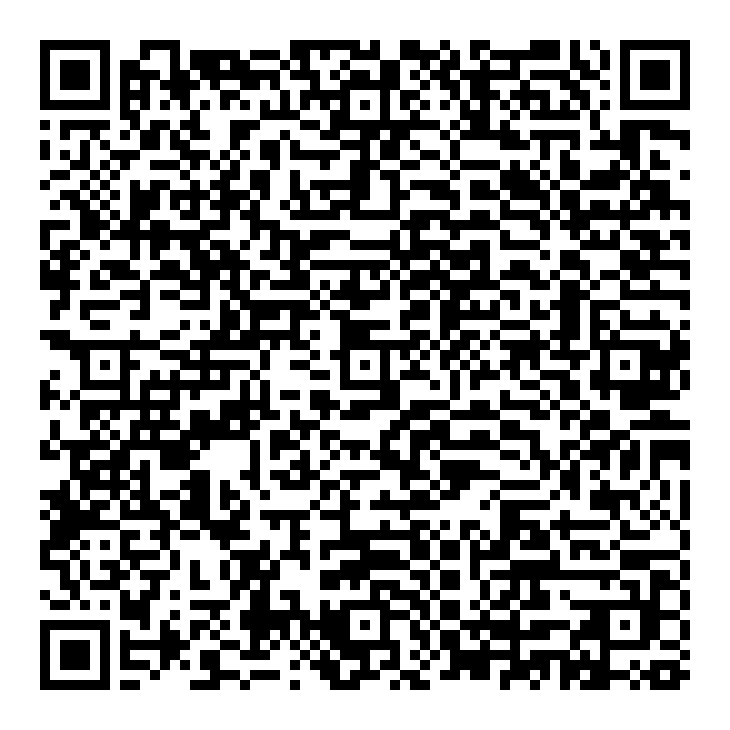C092 | 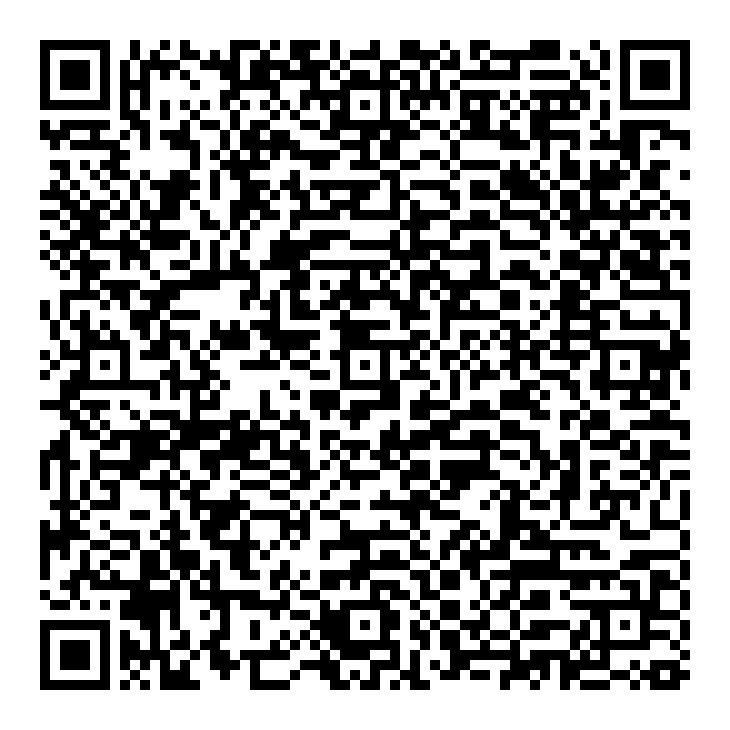C094 |
| 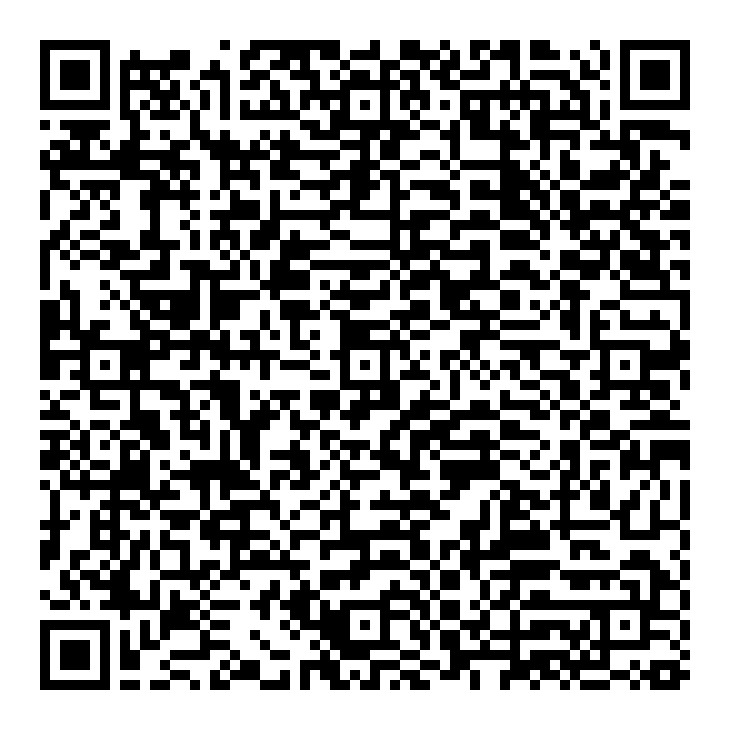C116 | 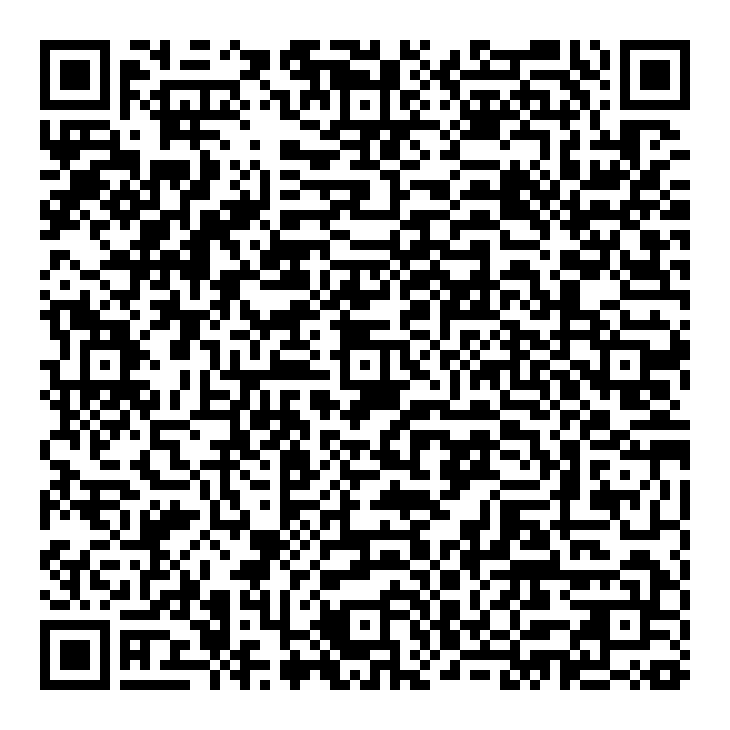C117 | 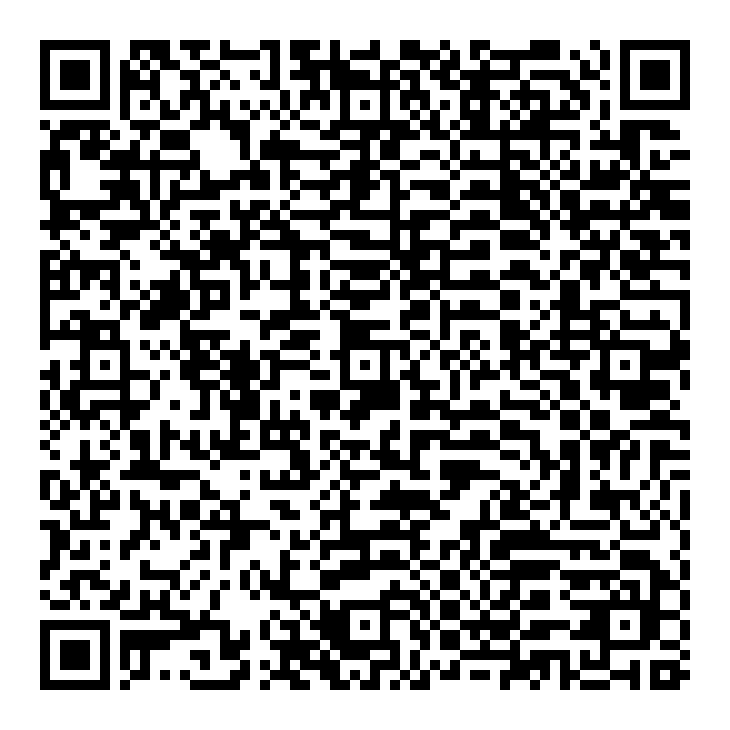C118 | 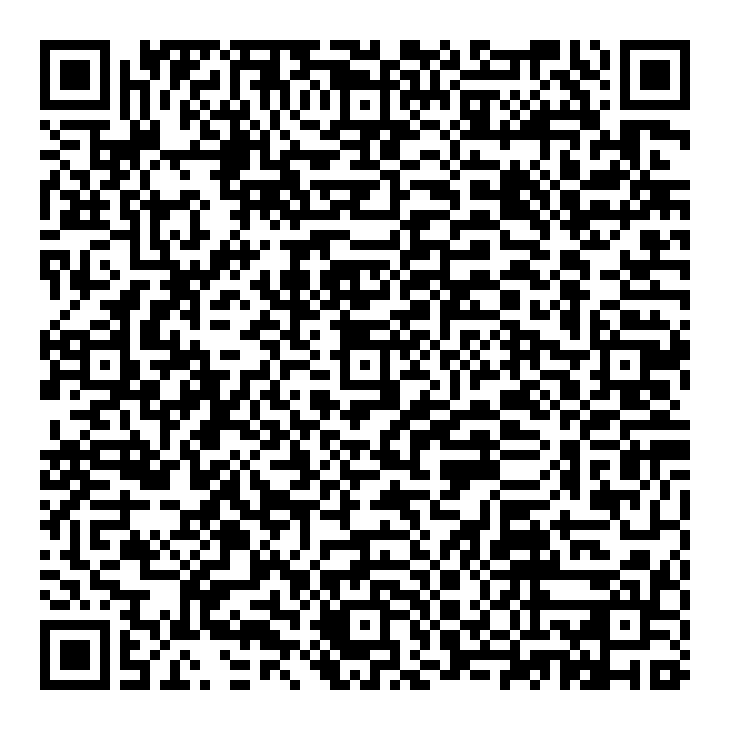C124 |
| 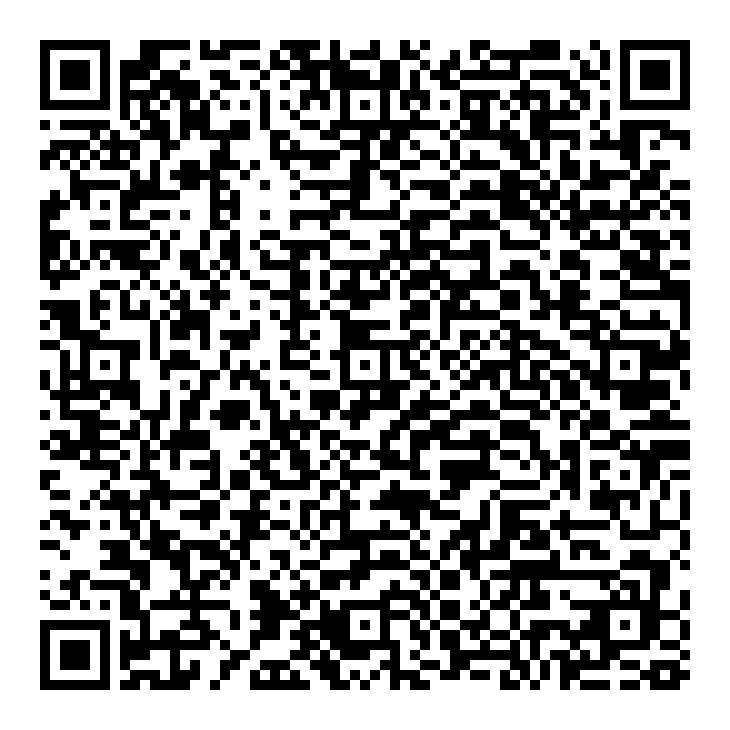C126 | 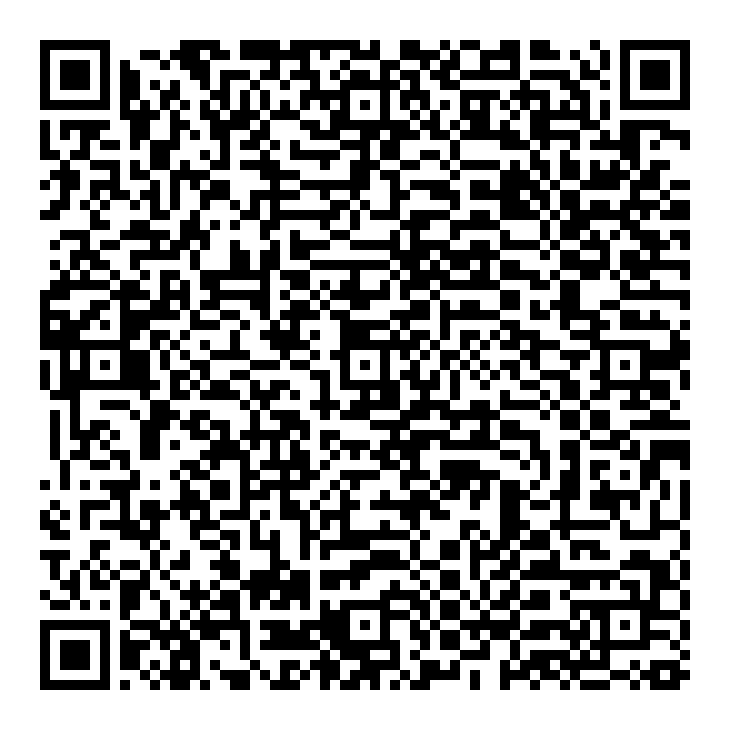C127 | 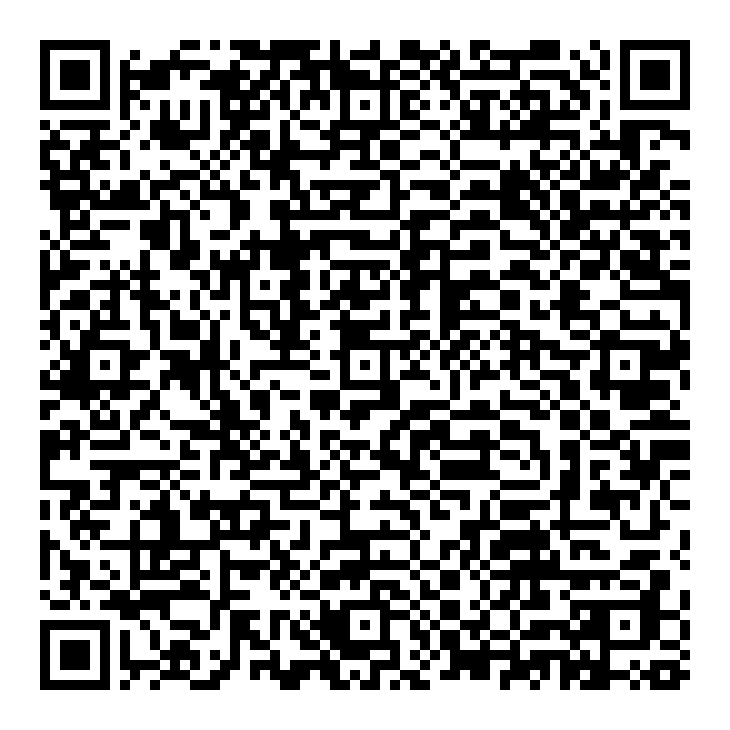C134 | 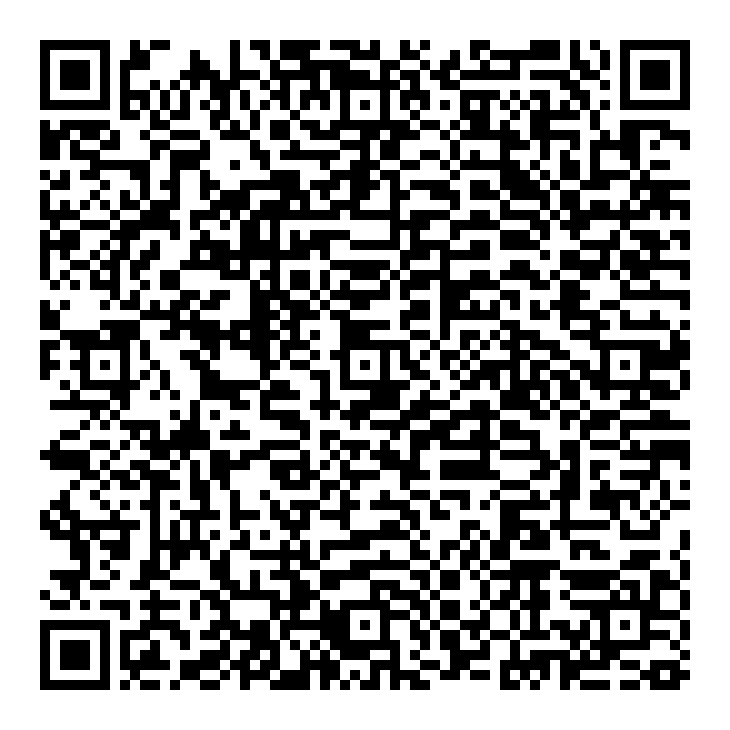C135 |
| 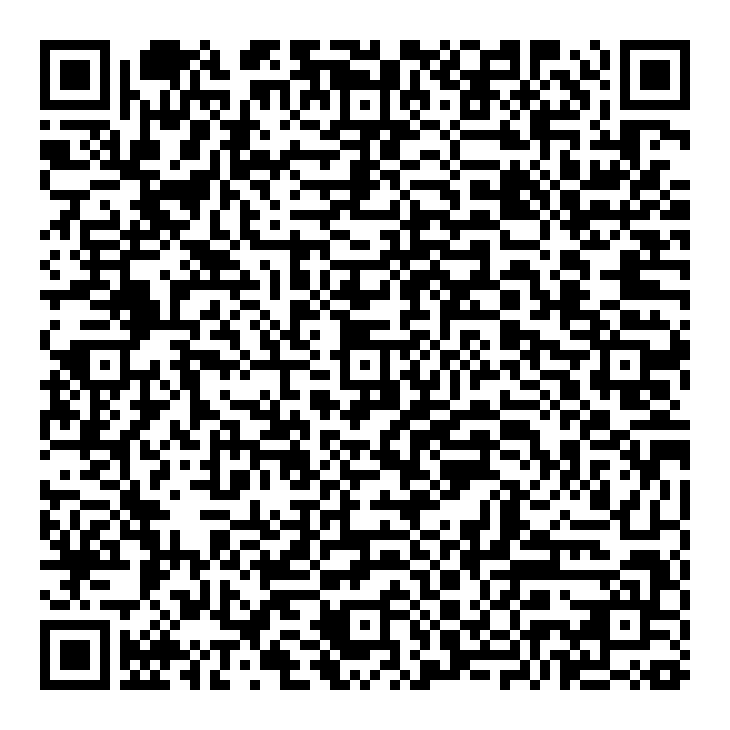C152 | 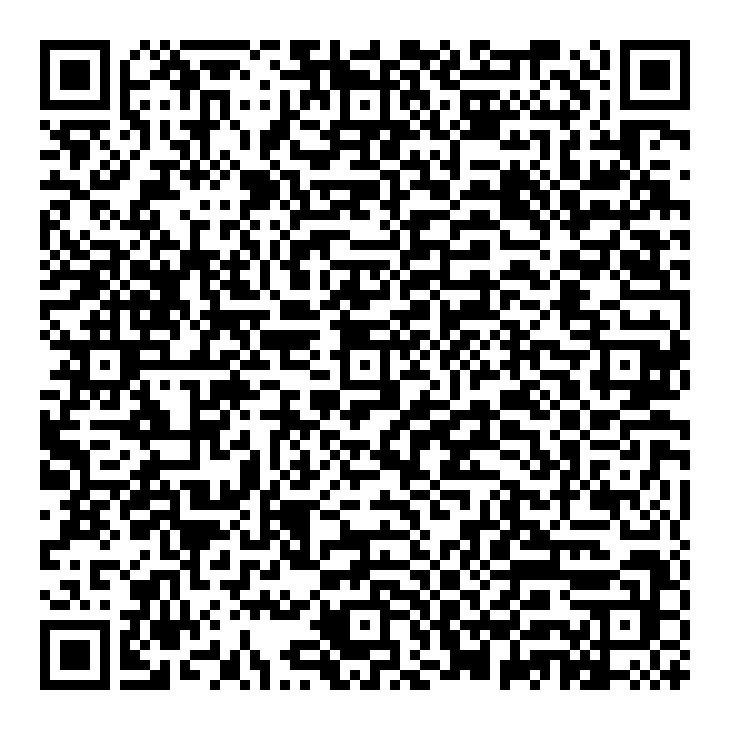C154 | 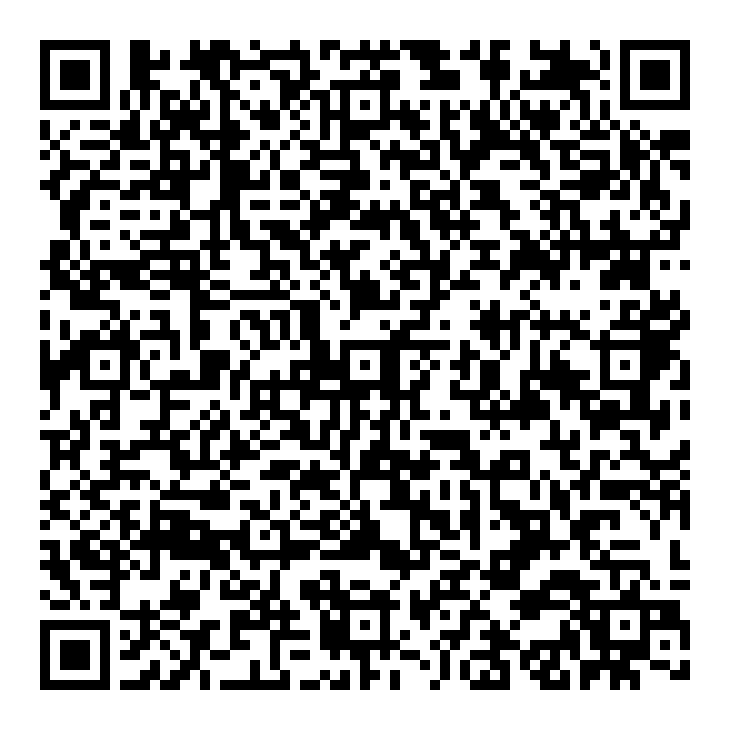C158 | 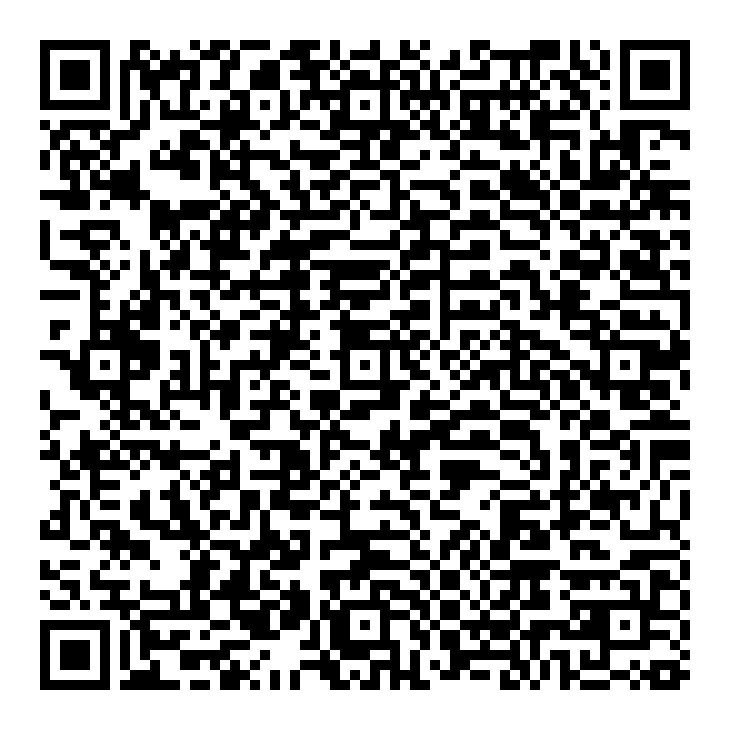C192 |
| 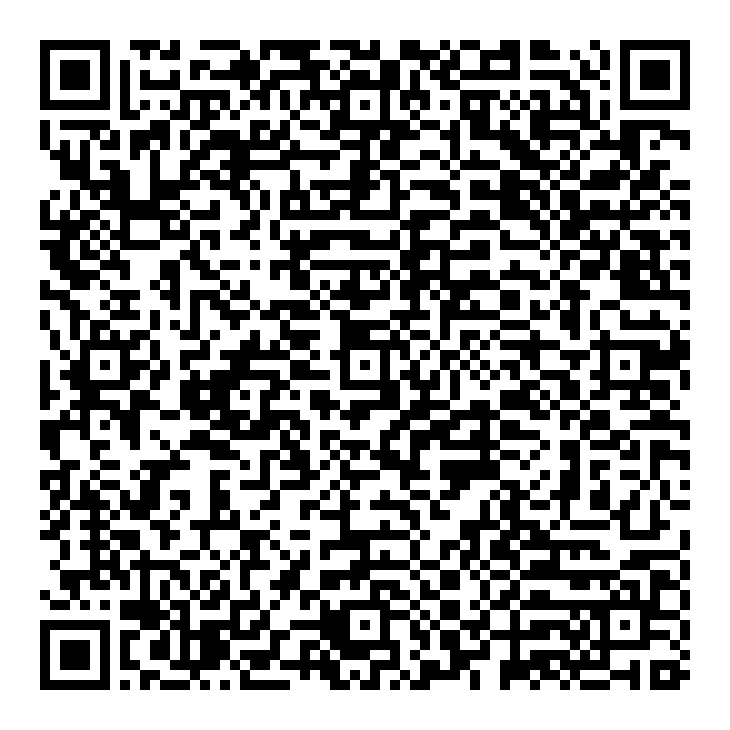C198 | 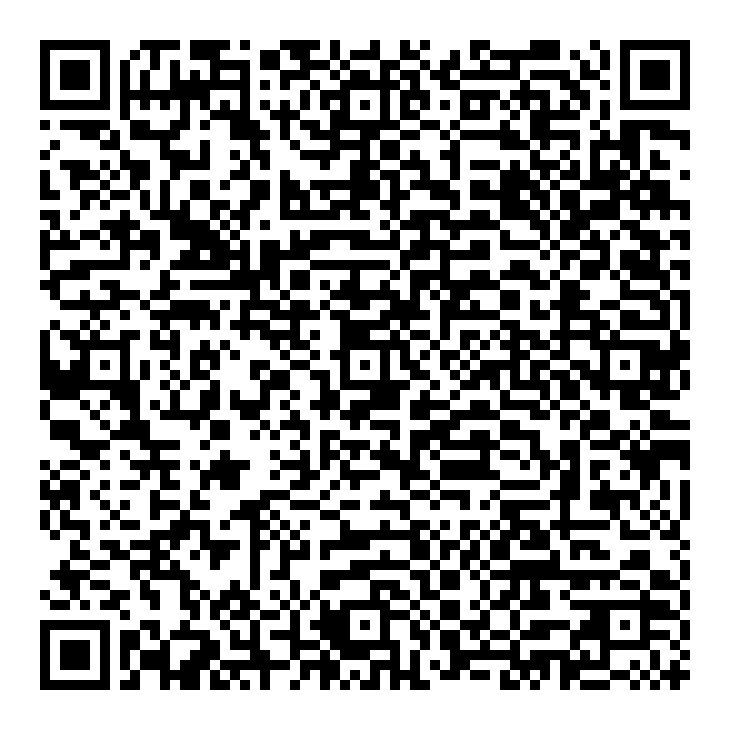C202 | 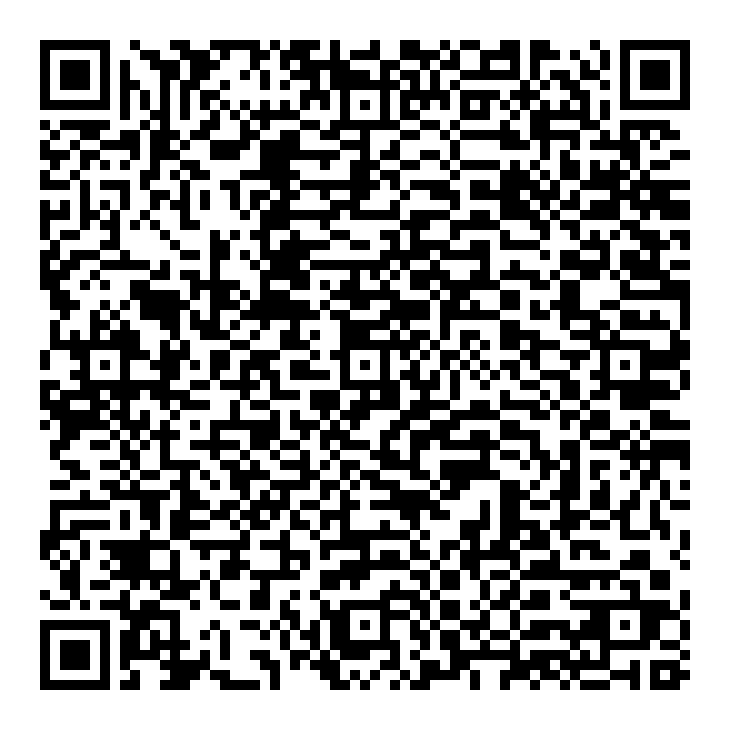C206 | 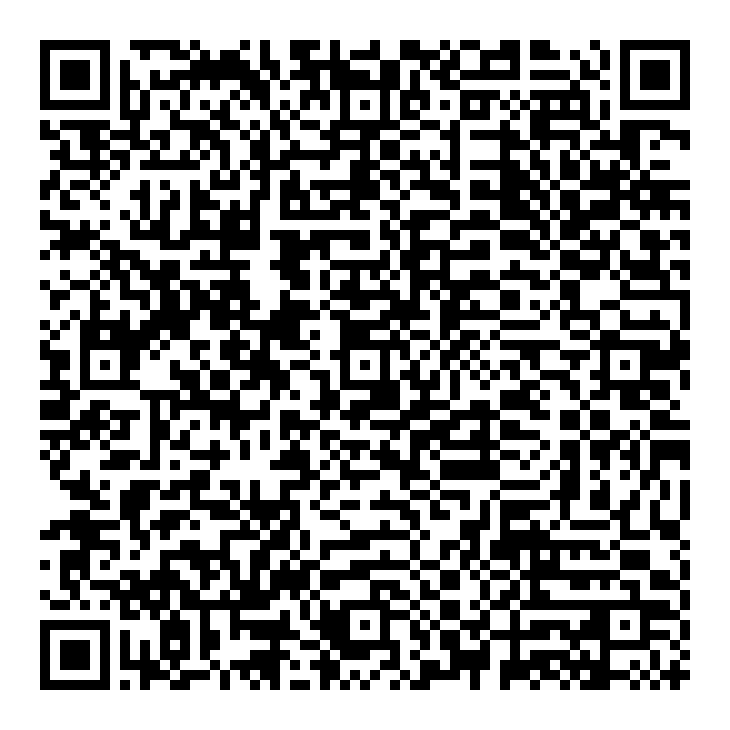C207 |
| 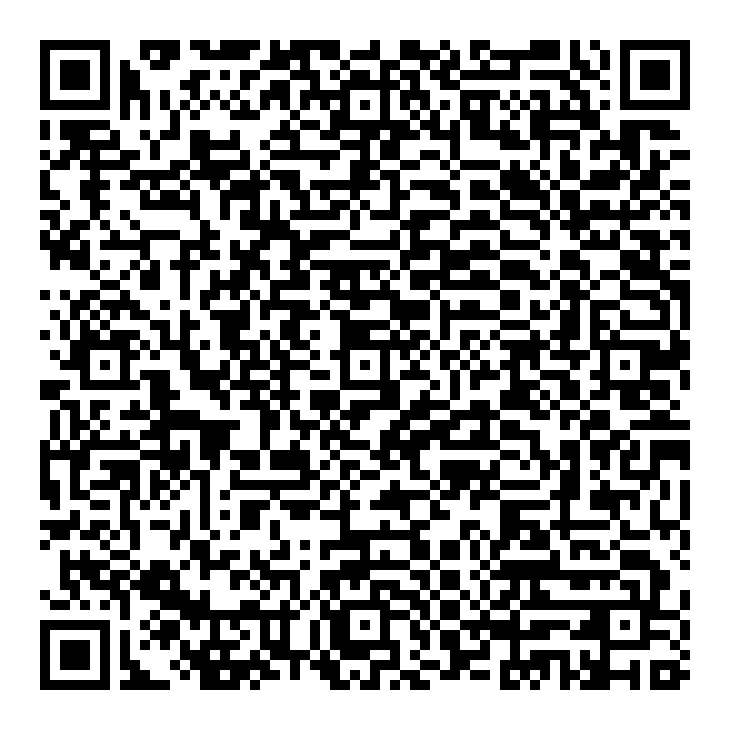C210 | 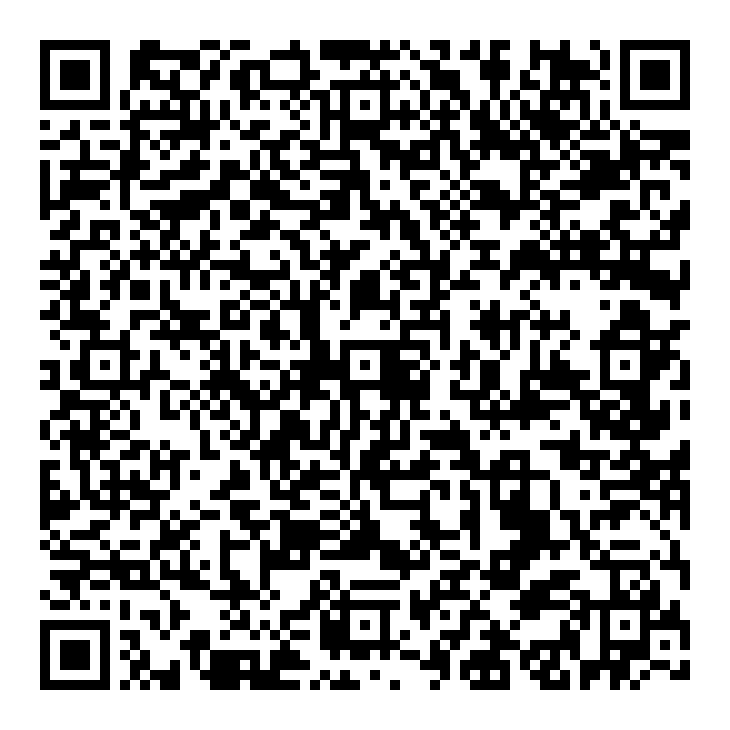C214 | 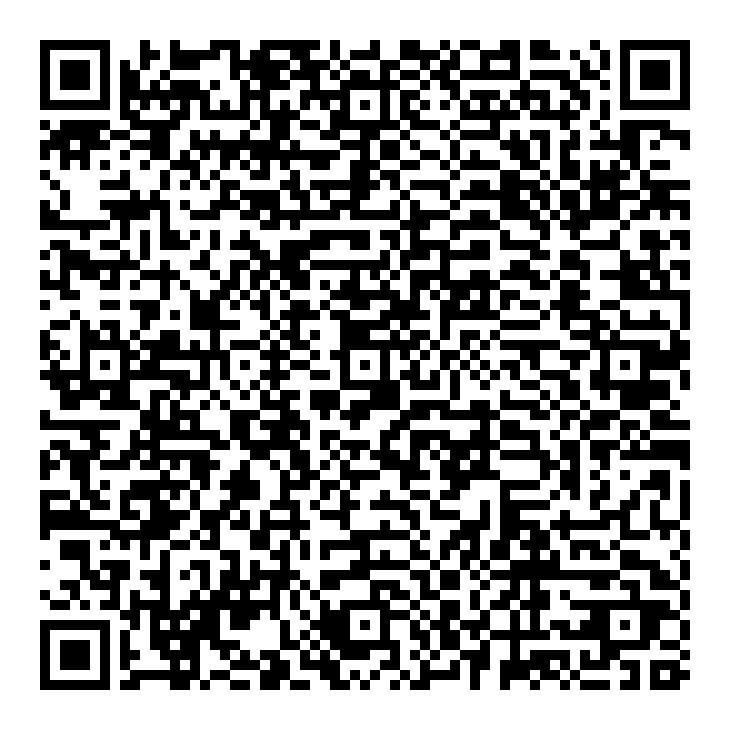C217 | 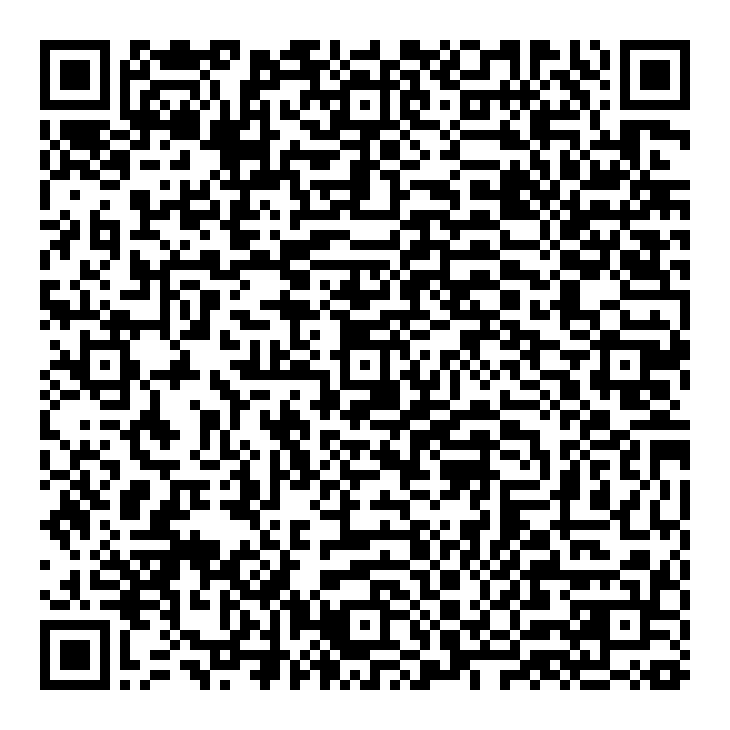C223 |
| 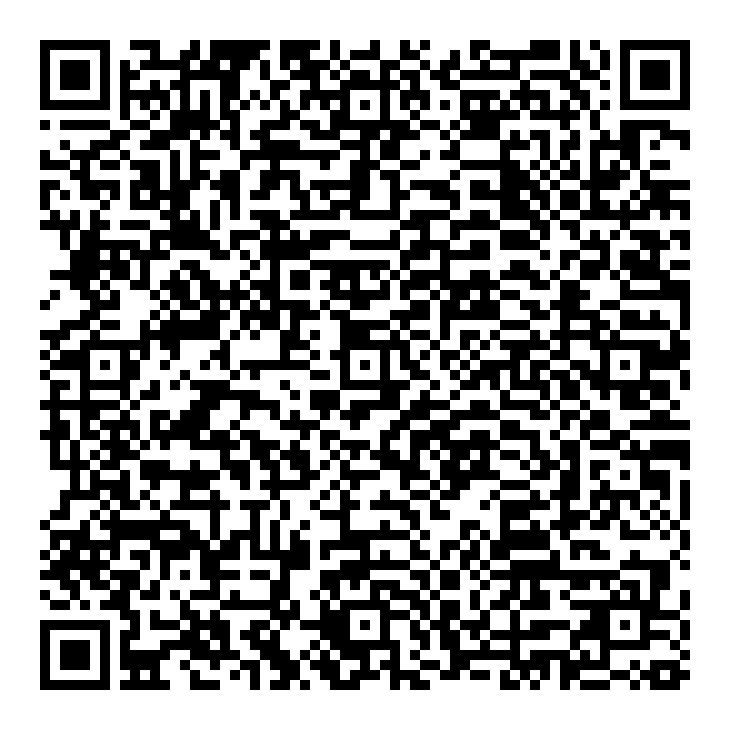C226 | 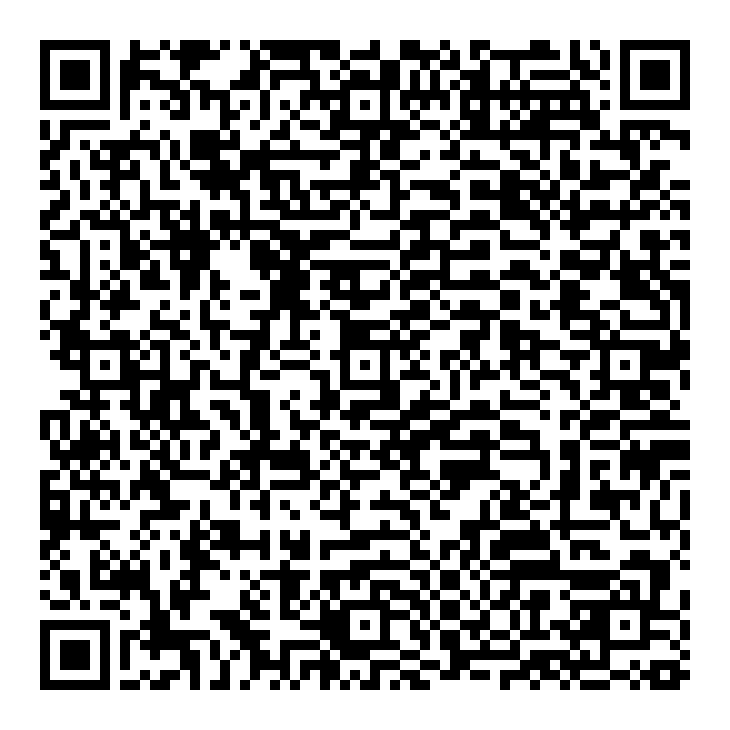C228 | 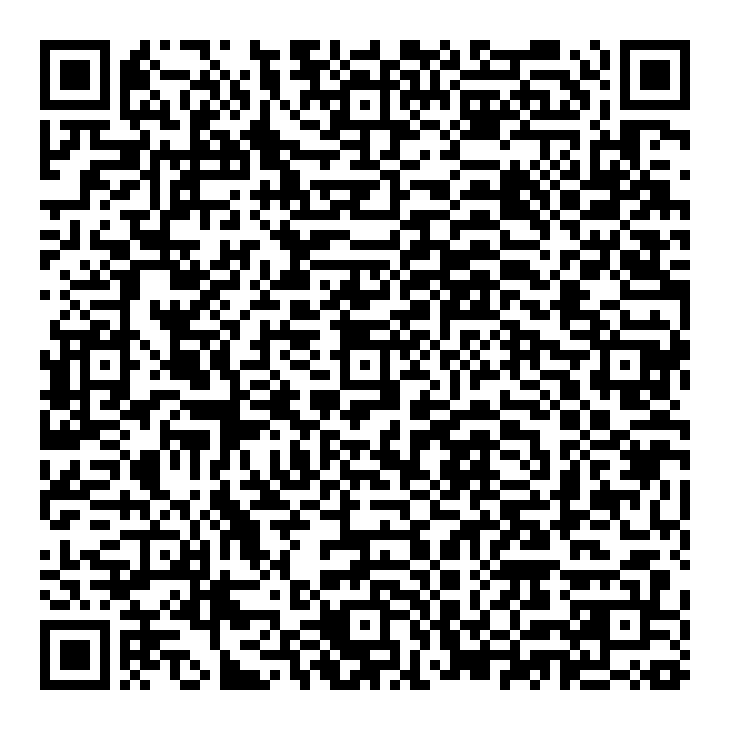C233 | 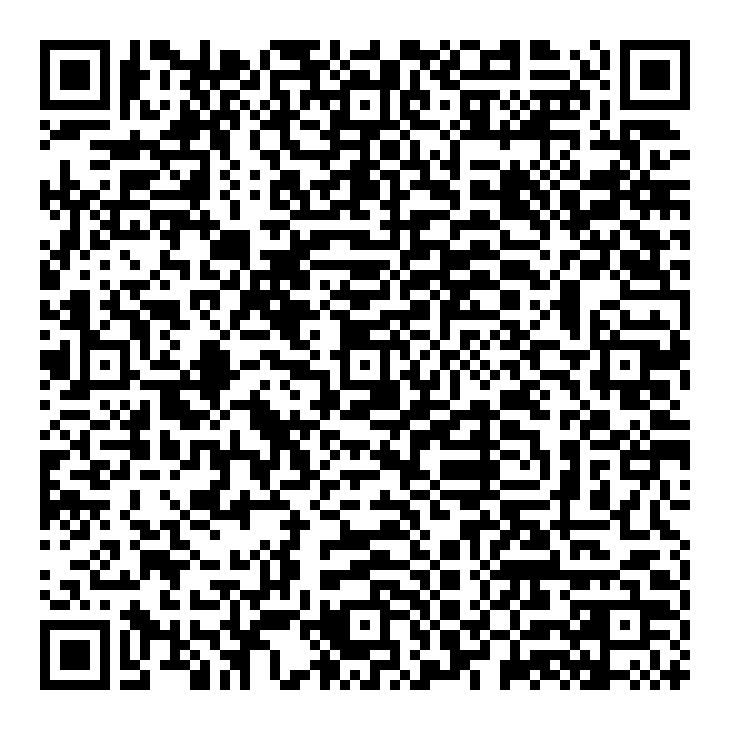C235 |
| 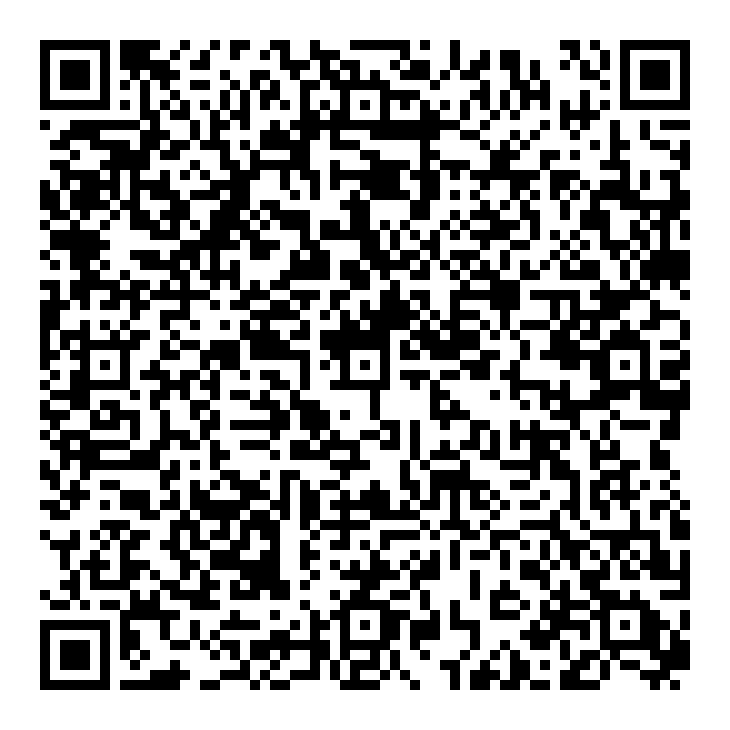C241 | 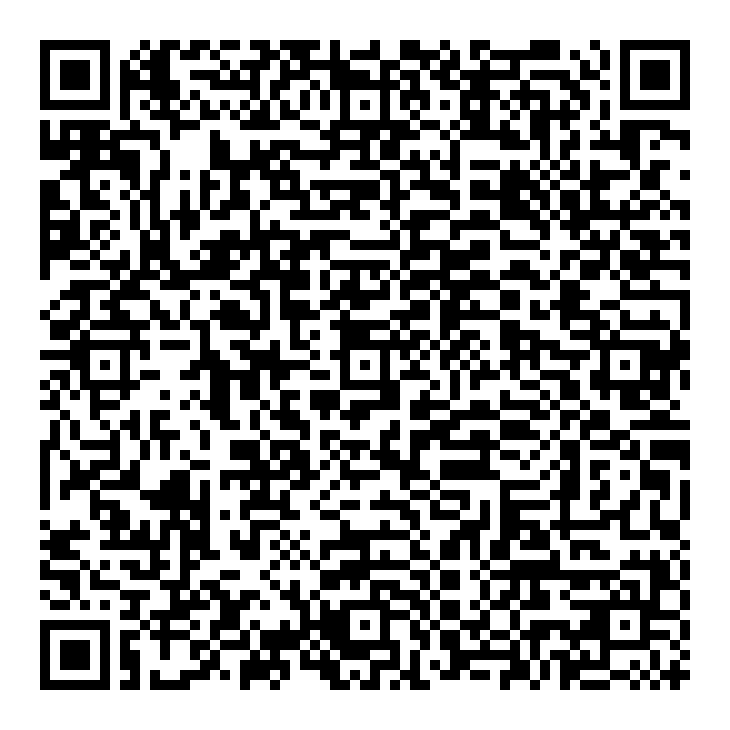C250 | 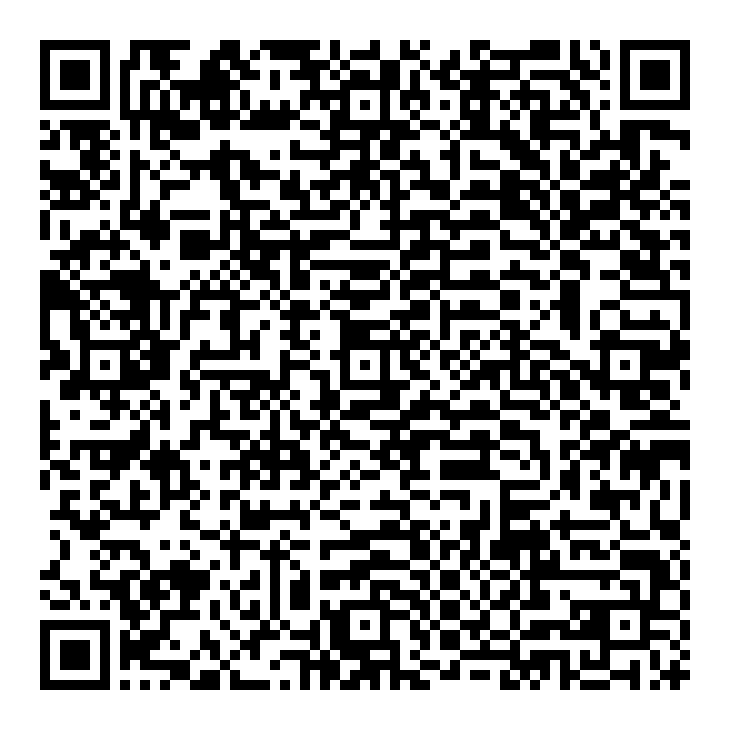C251 | 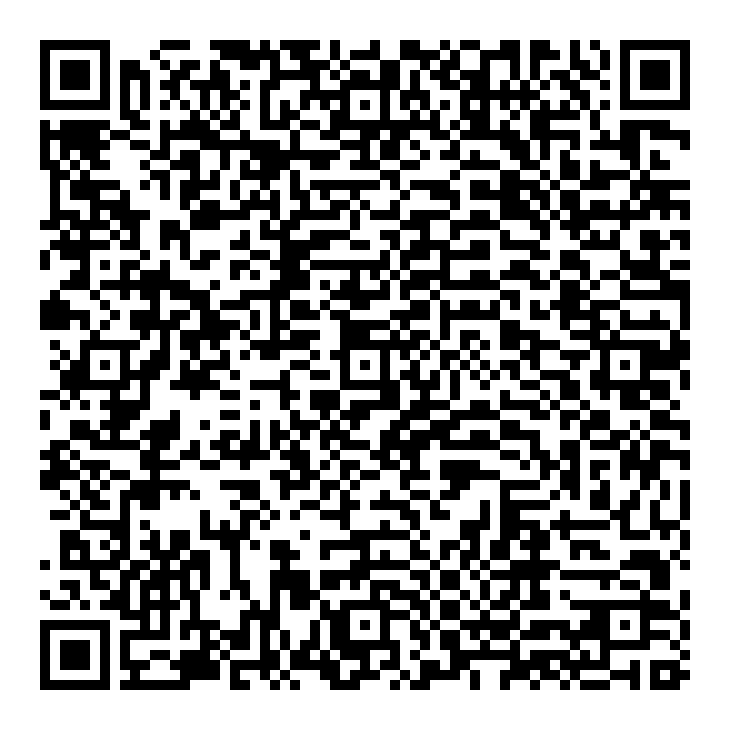C252 |
| 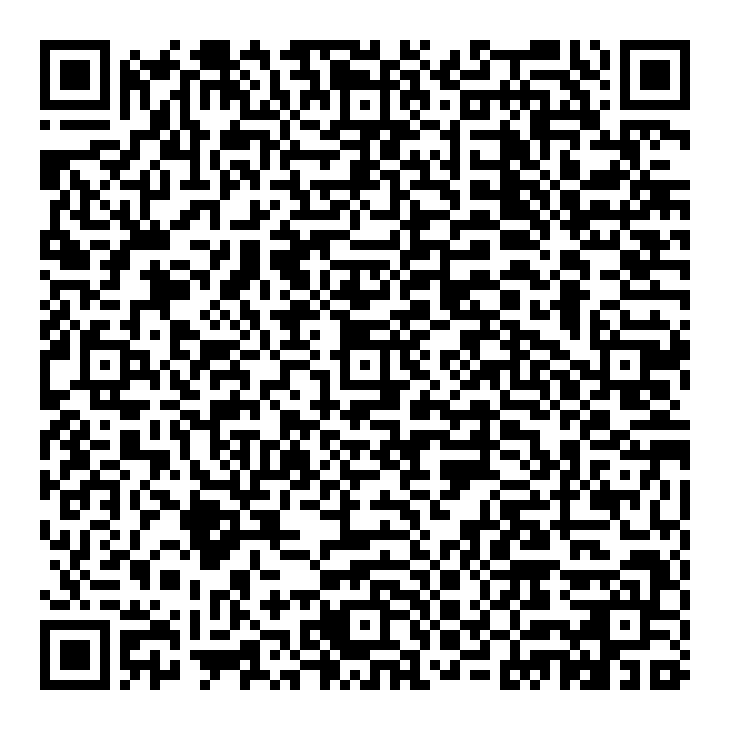C253 | 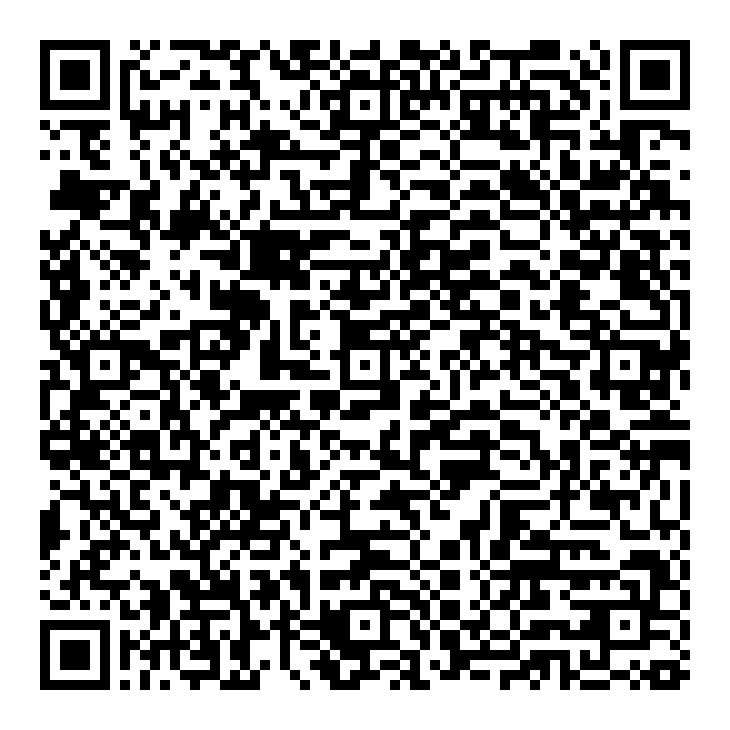C255 | 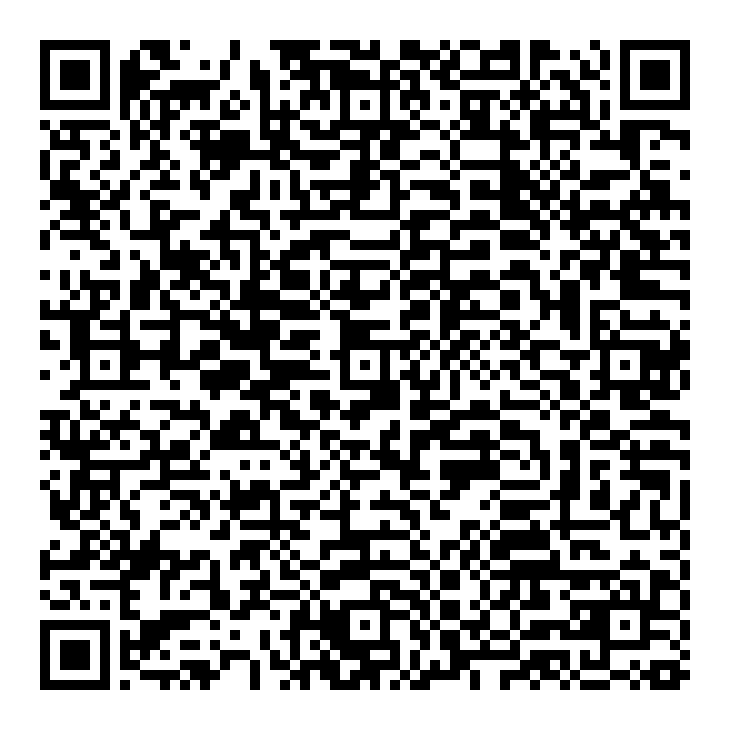C260 | 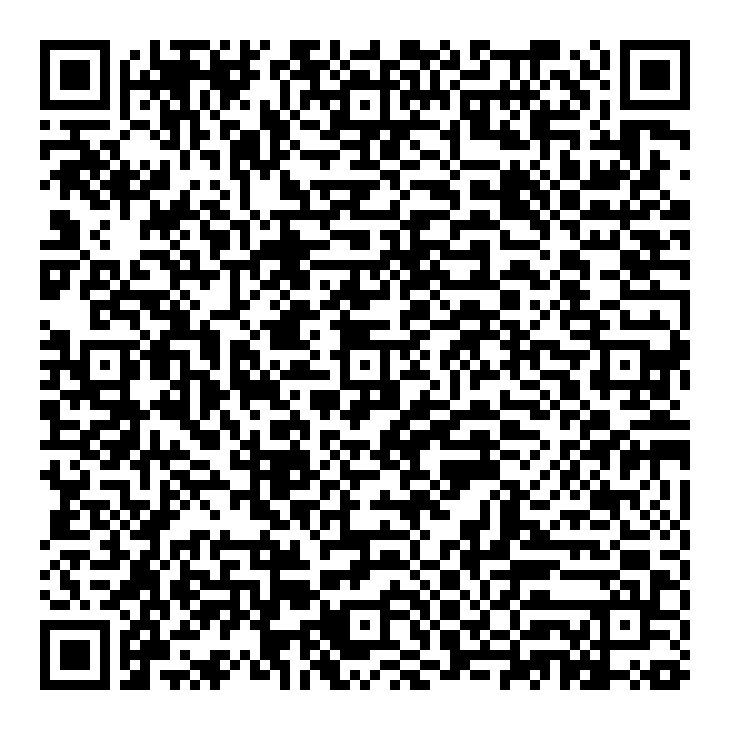C268 |
| 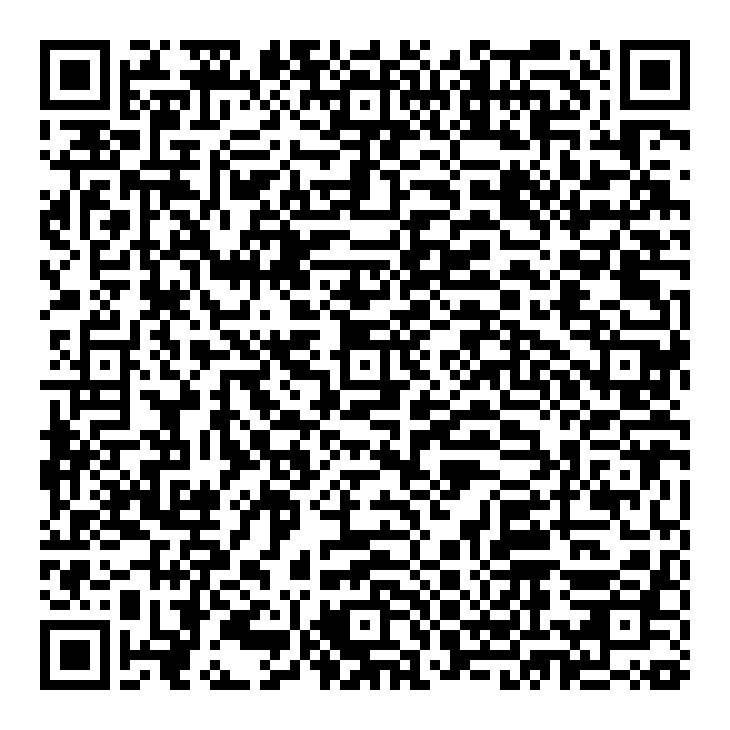C275 | 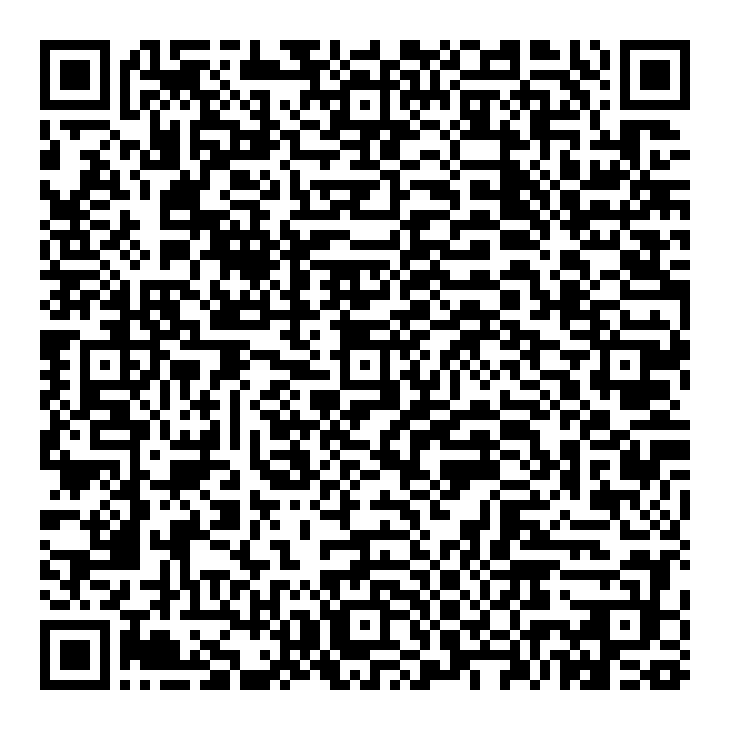C283 | 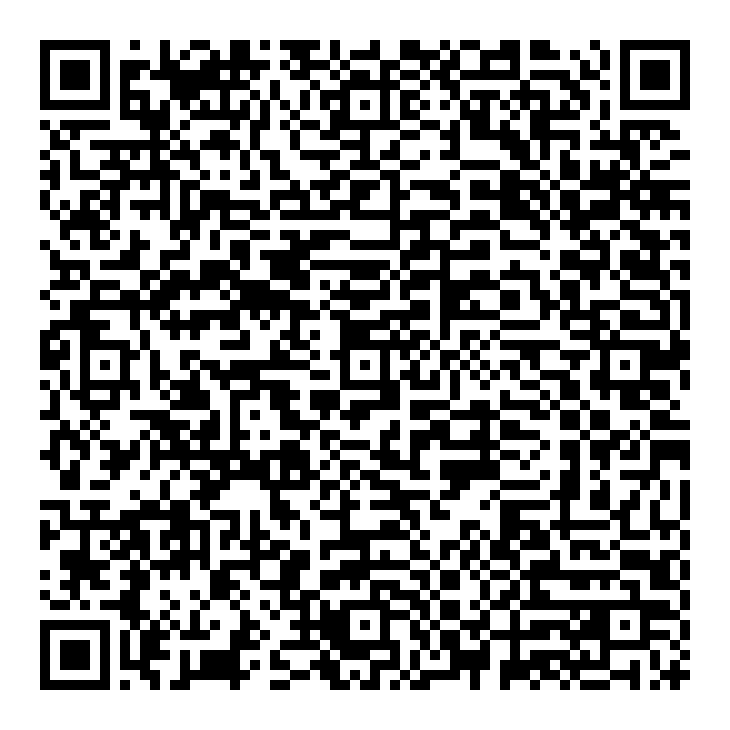C285 | 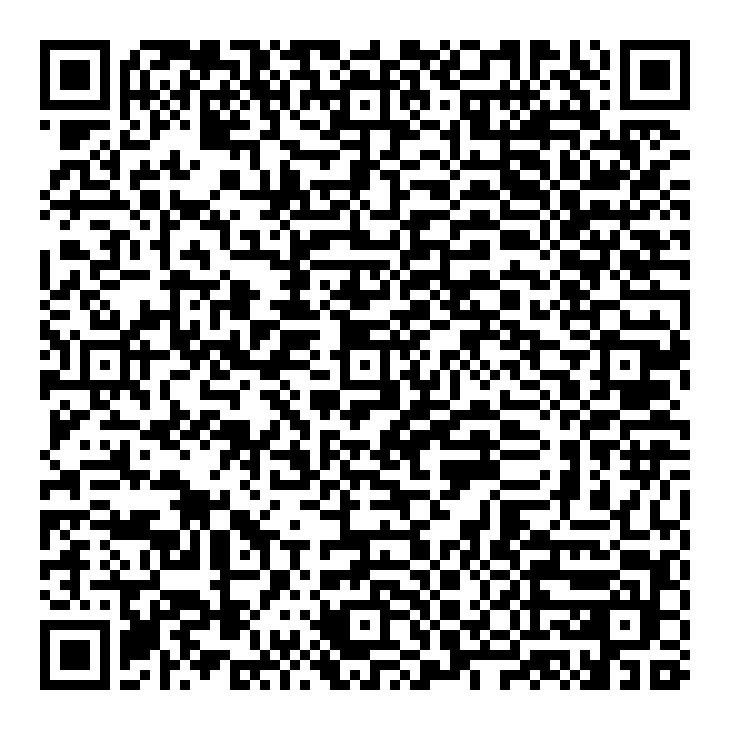C286 |
| 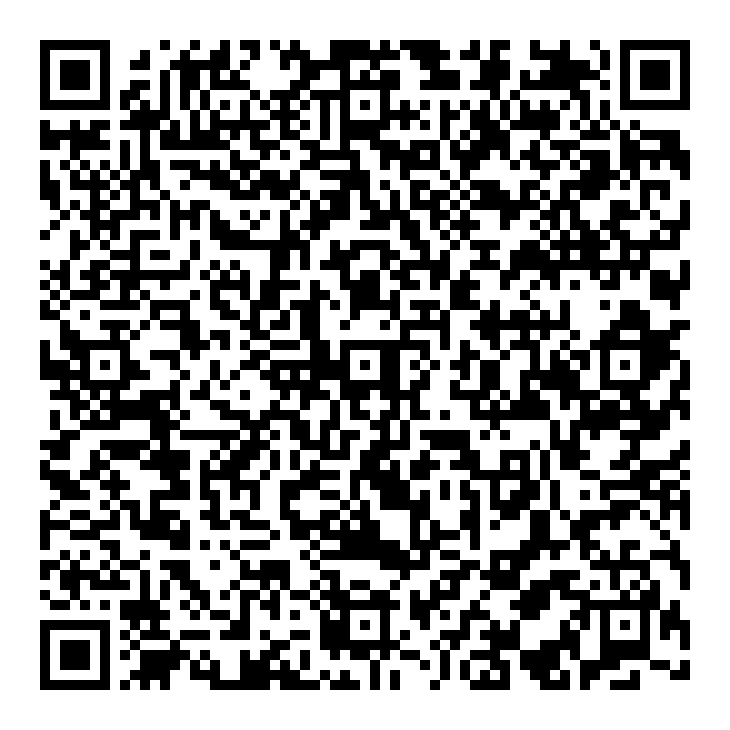C288 | 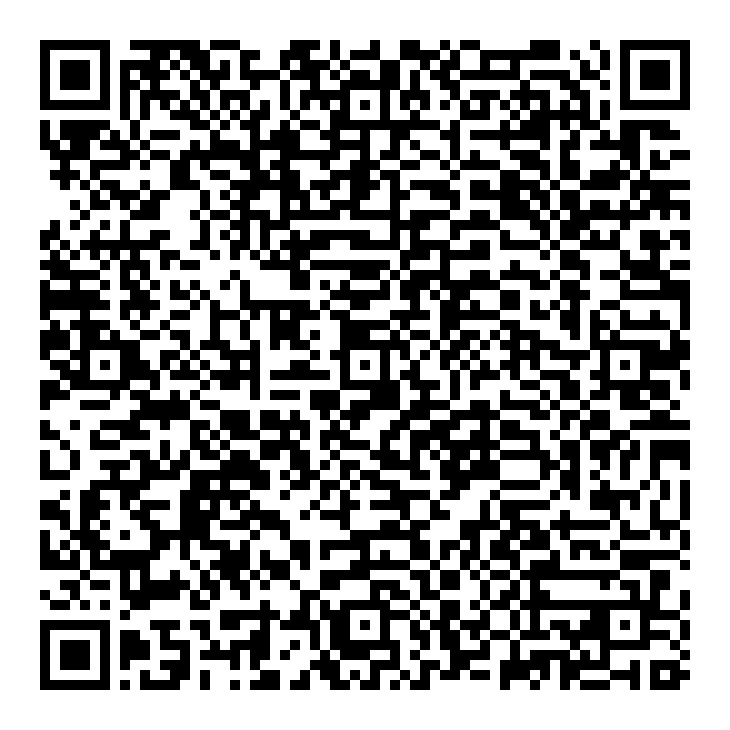C290 | 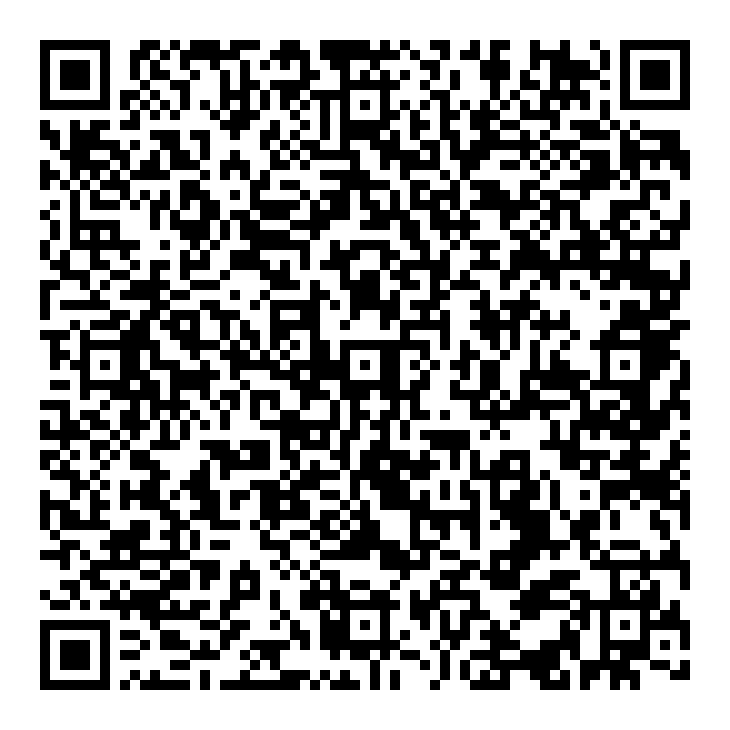C292 | 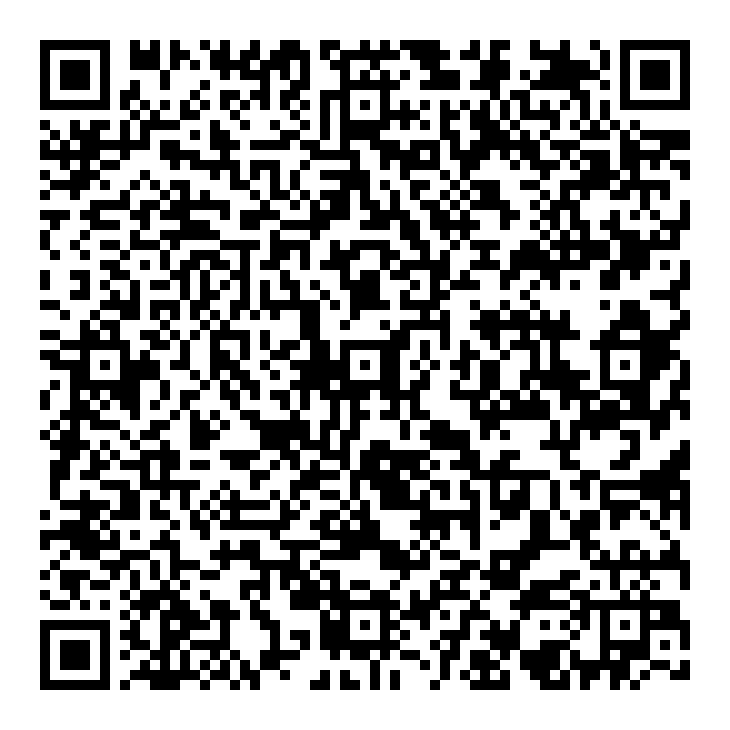C297 |
| 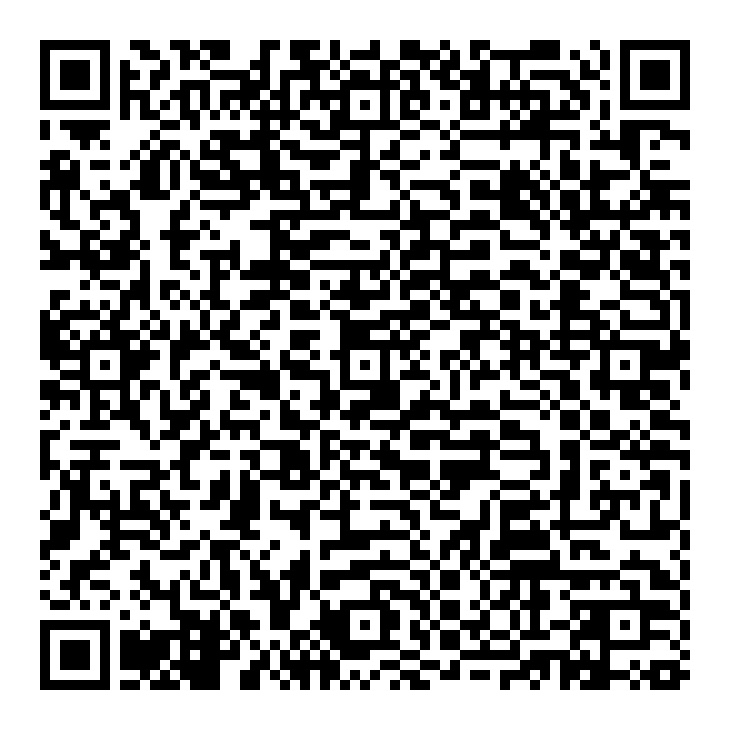C313 | 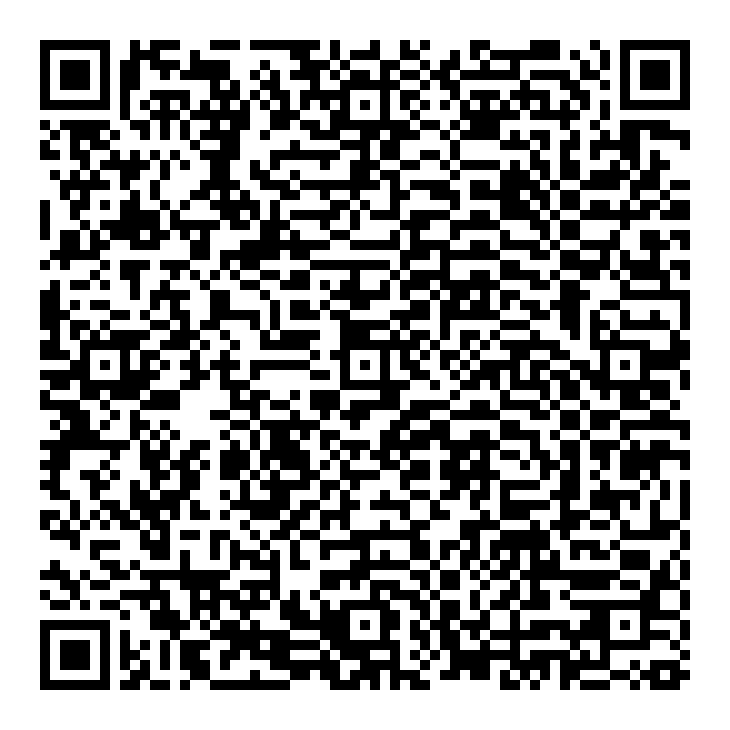C320 | 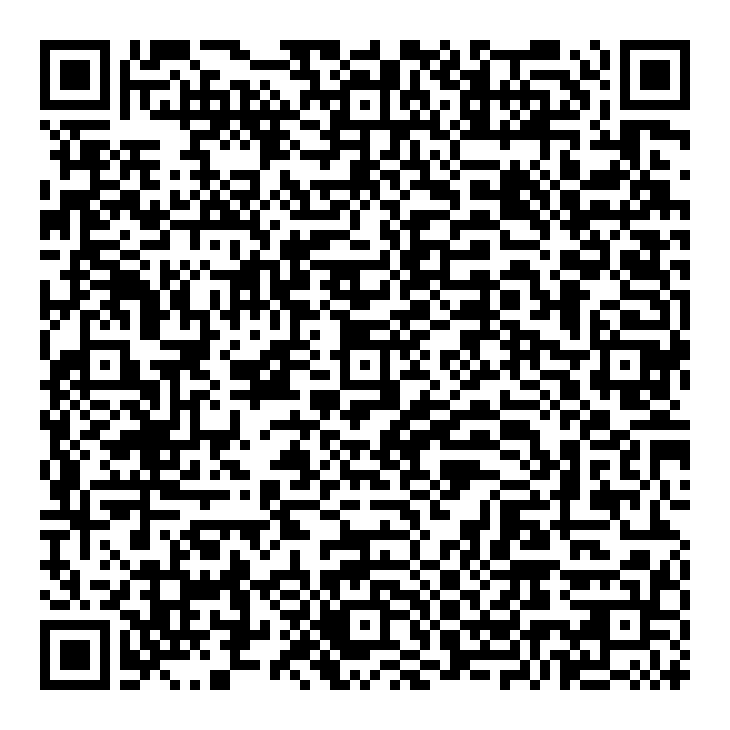C323 | 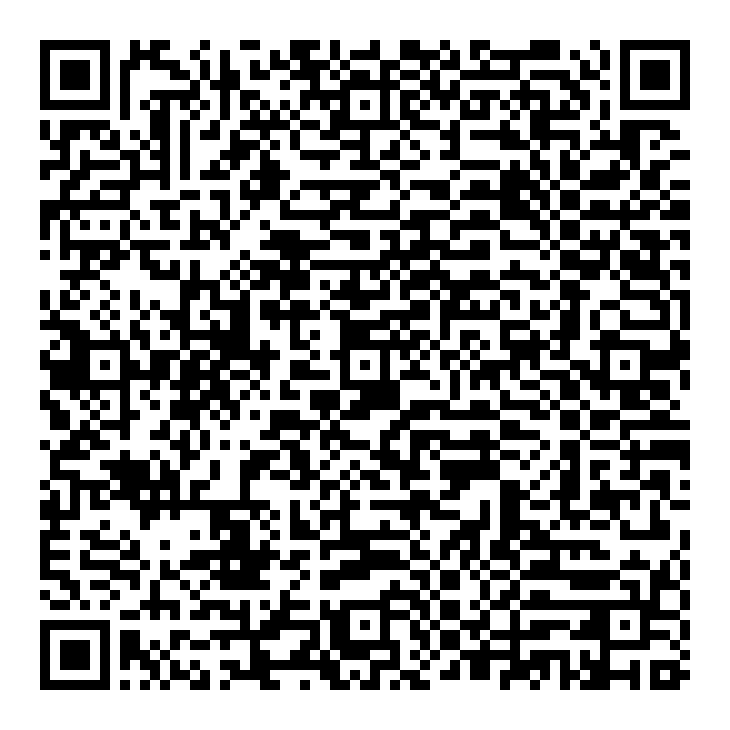C324 |
| 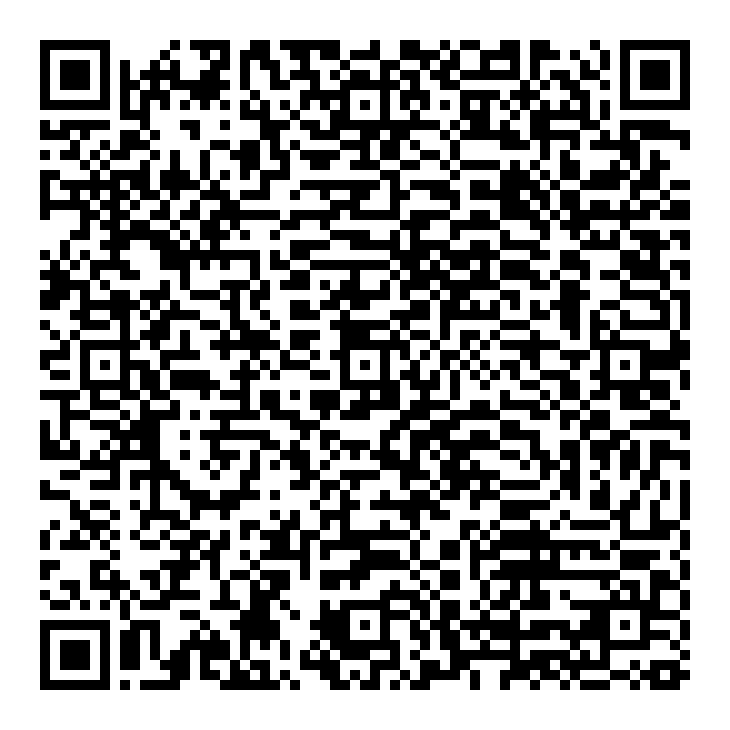C330 | 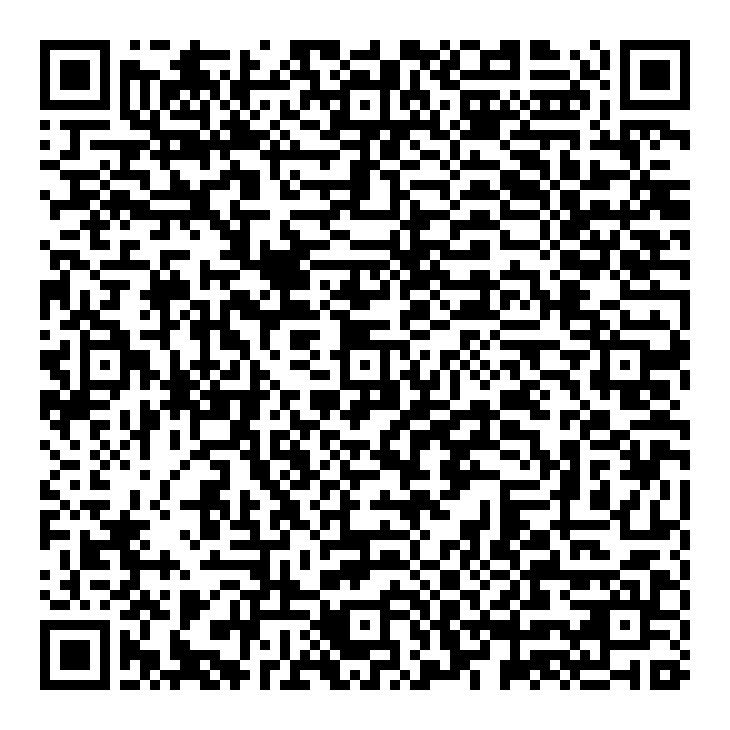C334 | 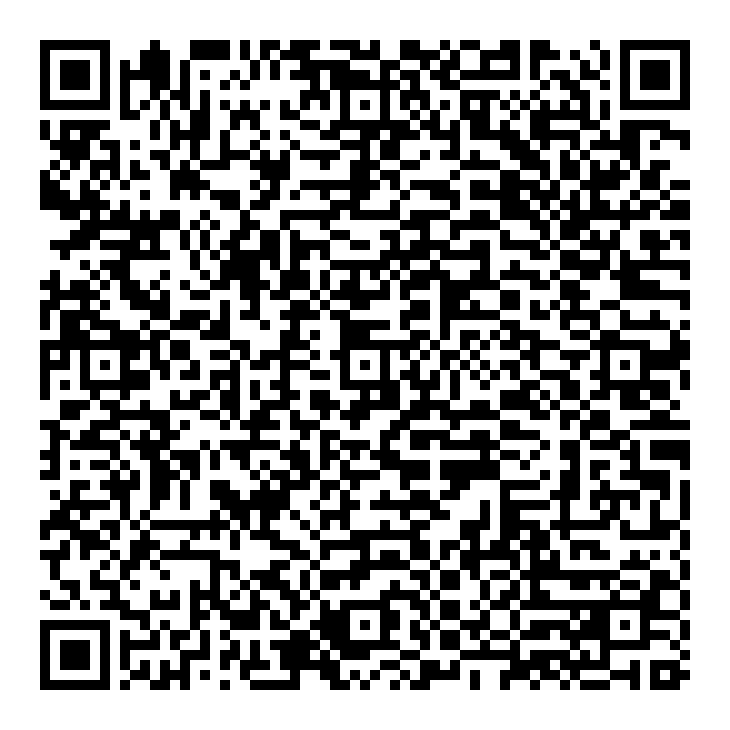C338 | 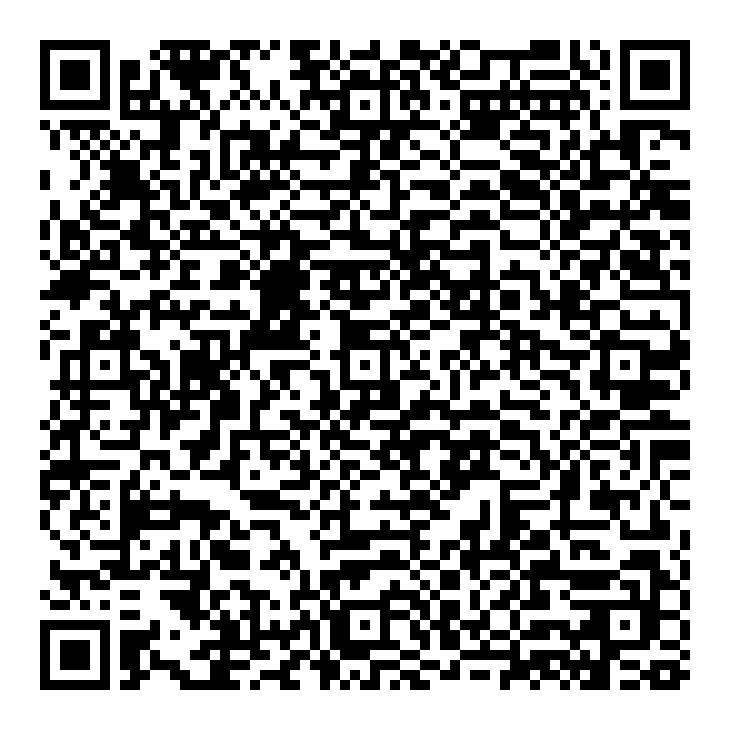C343 |
| 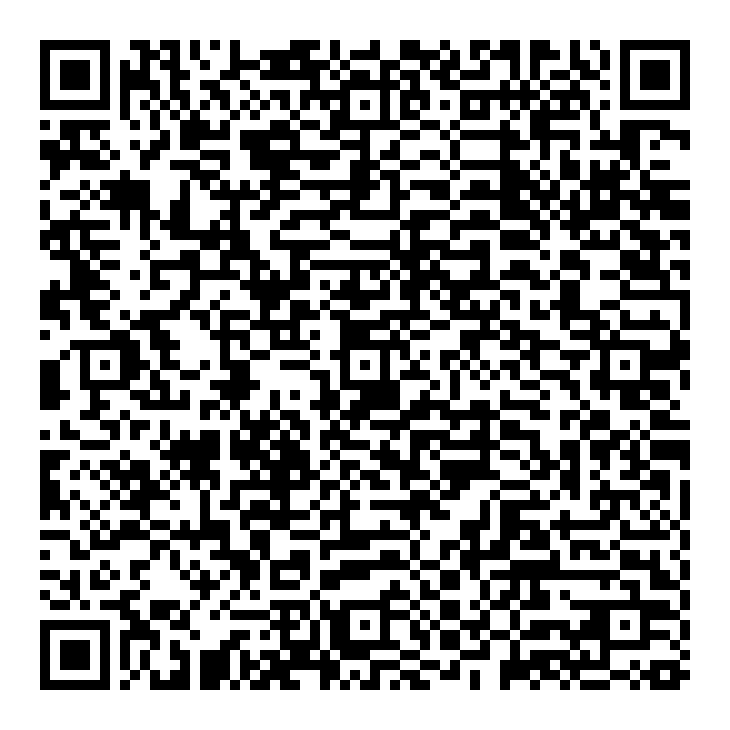C347 | 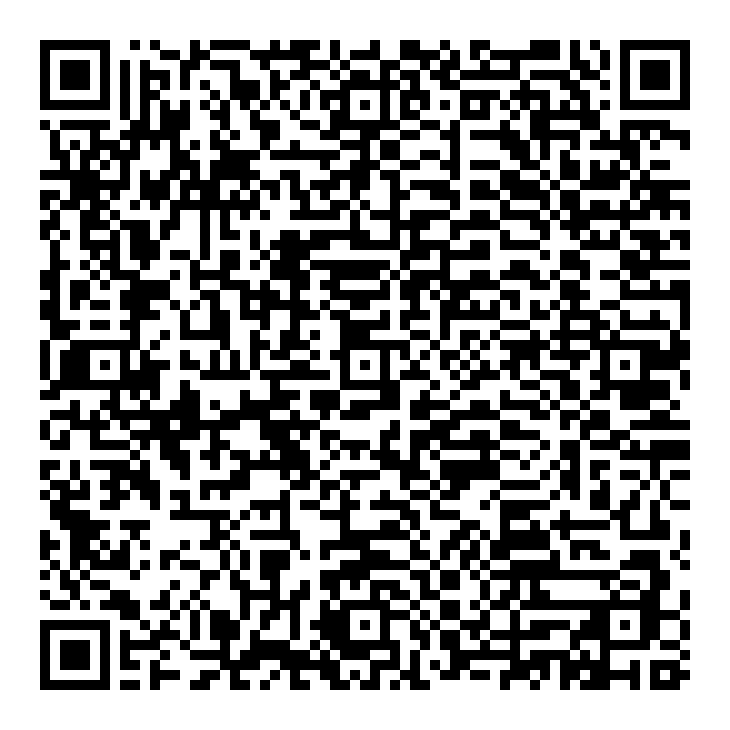C348 | 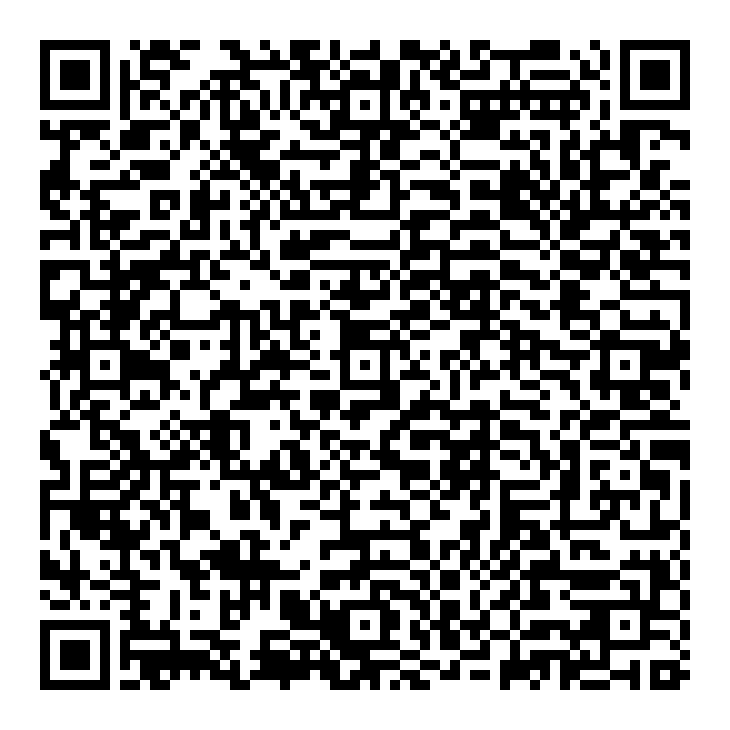C350 | 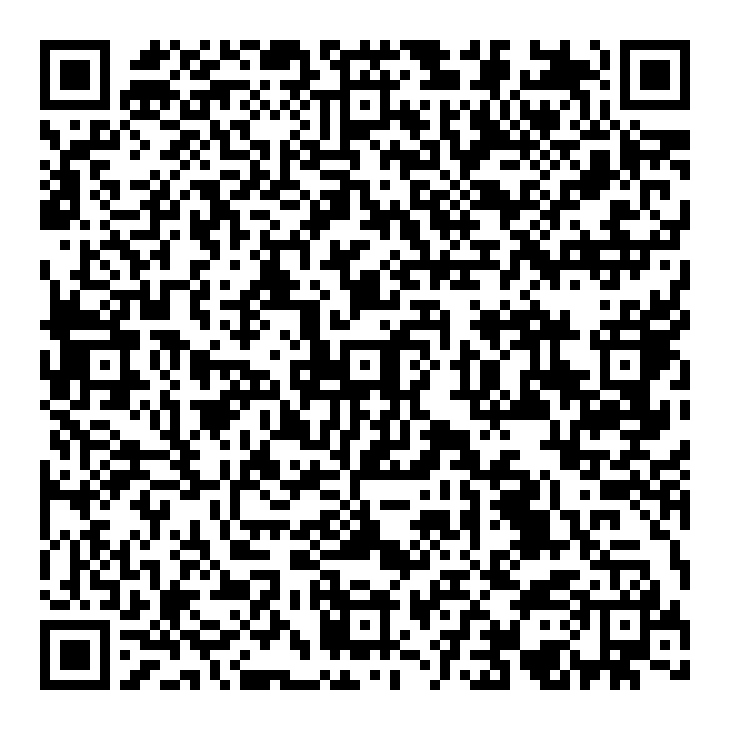C354 |
| 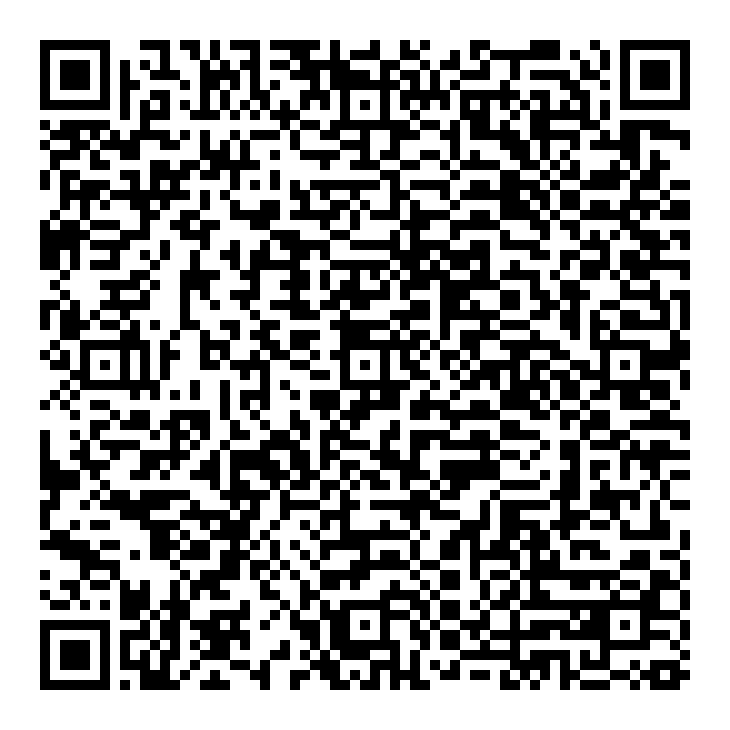C362 | 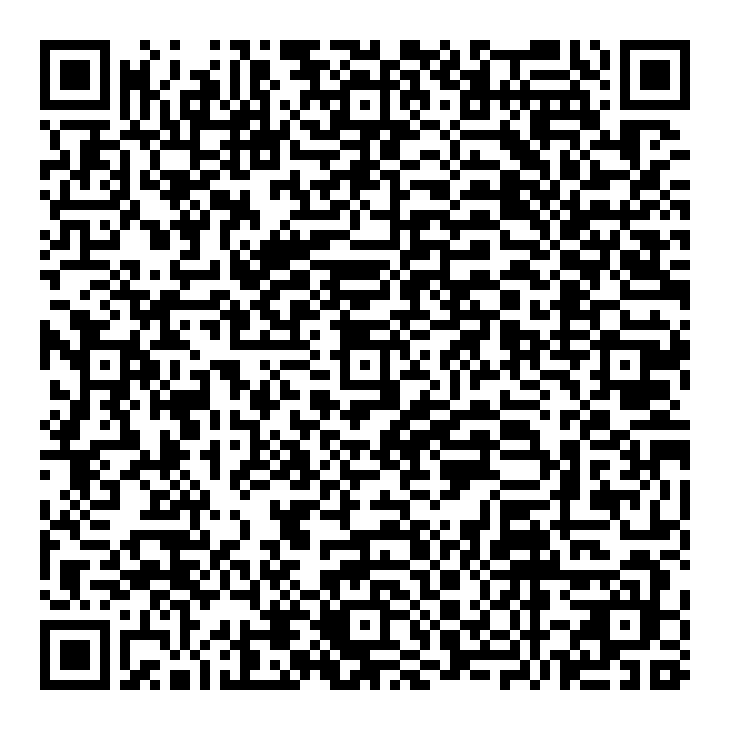C370 | 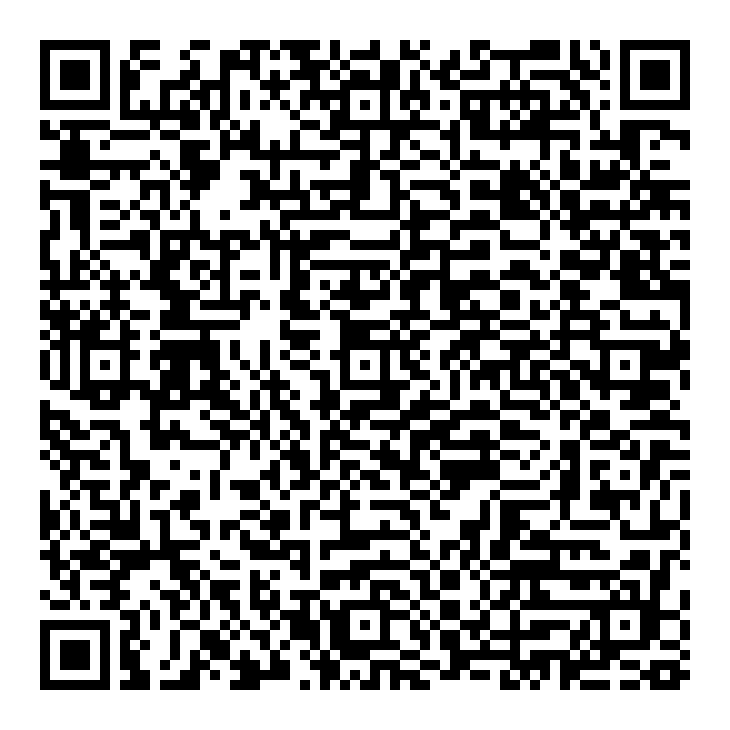C376 | 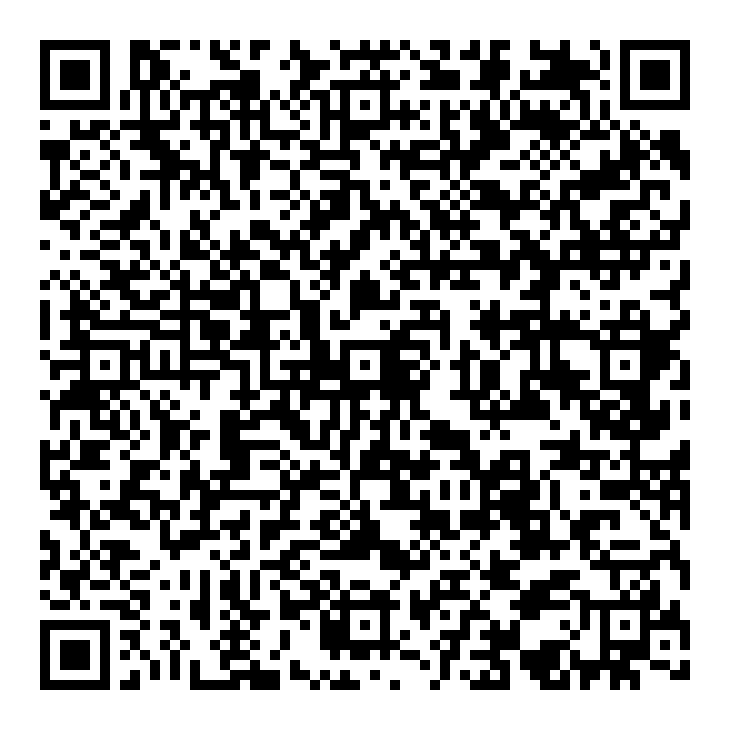C377 |
| 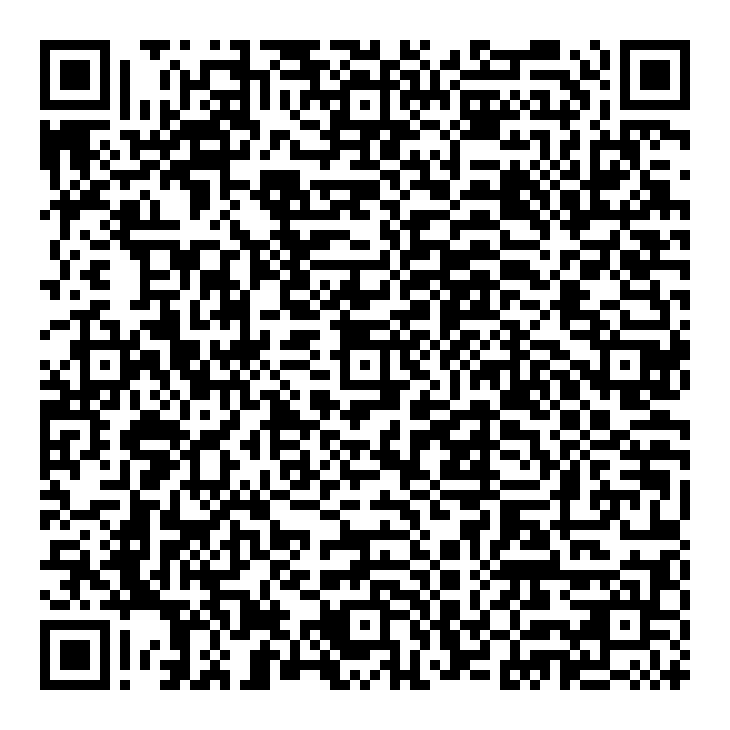C380 | 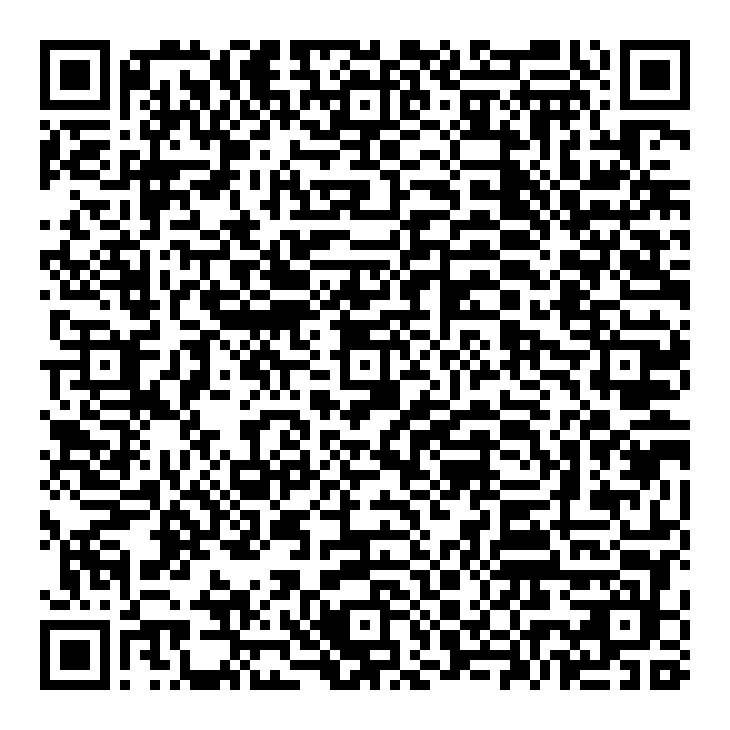C382 | 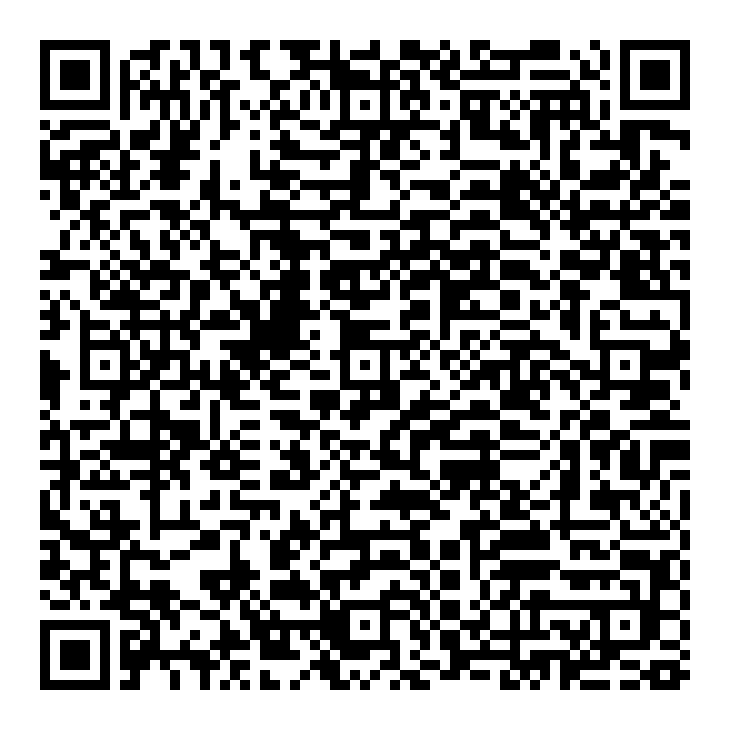C387 | 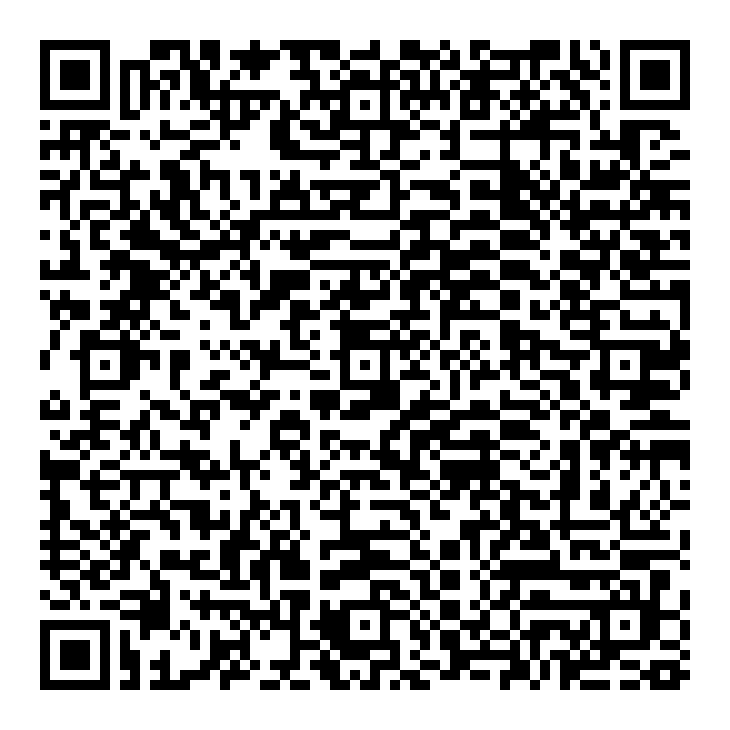C391 |
| 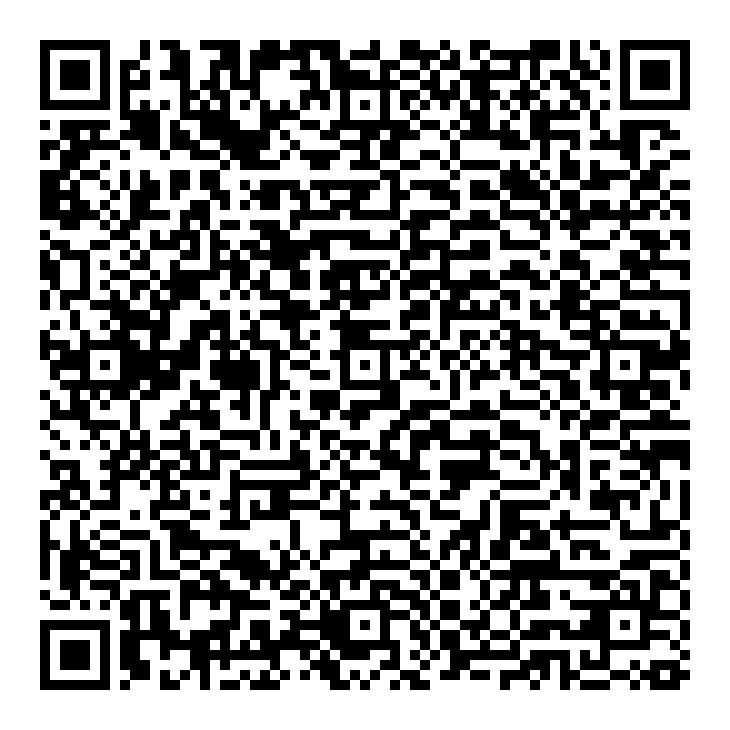C393 | 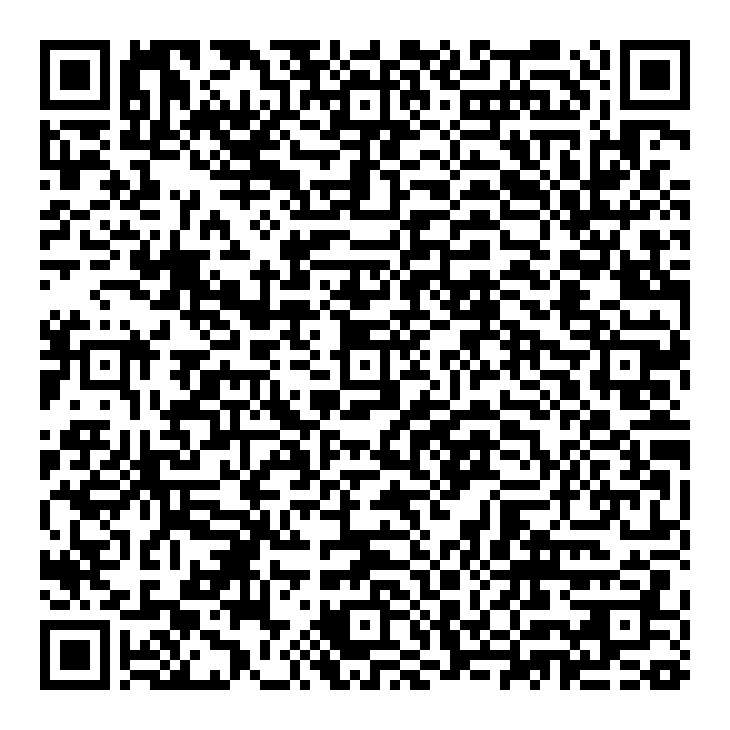C396 | 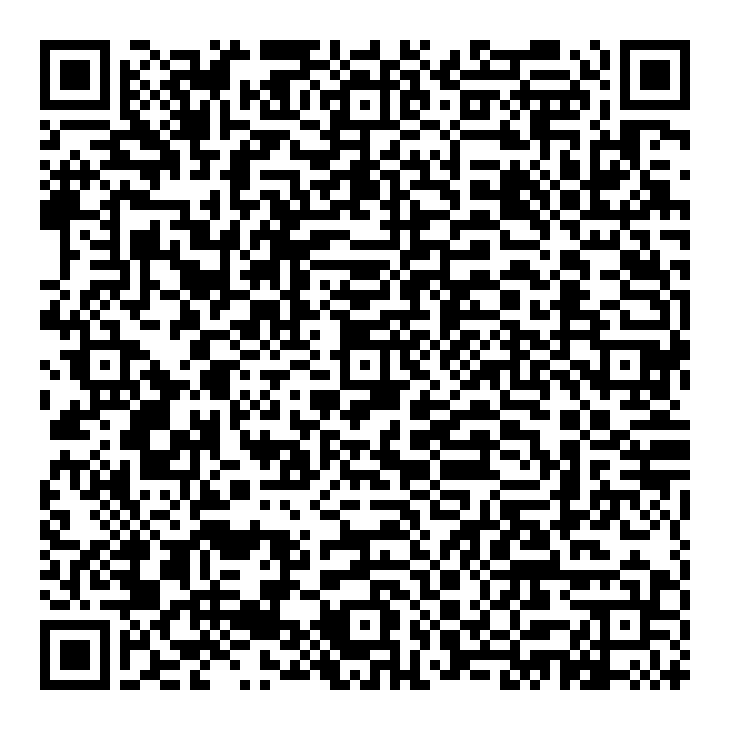C408 | 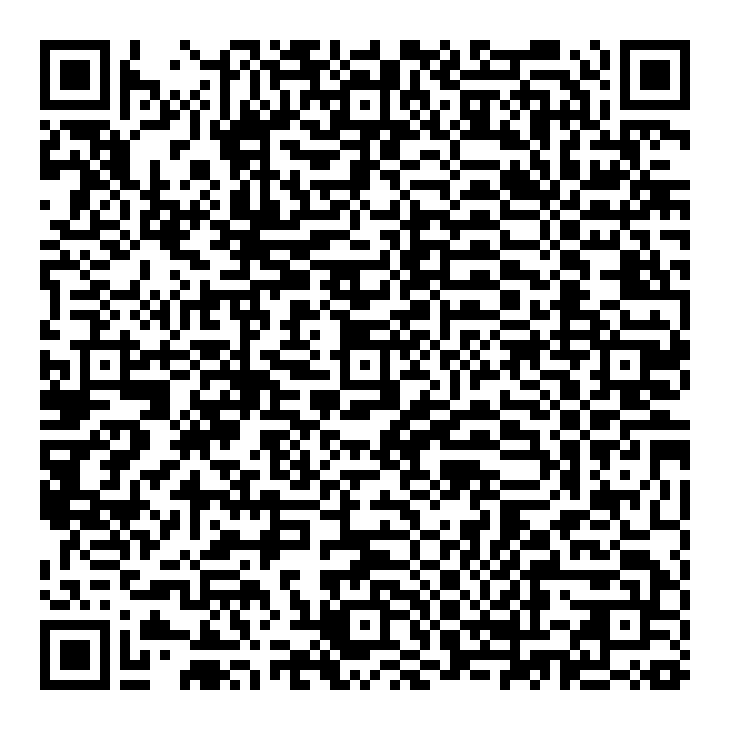C418 |
| 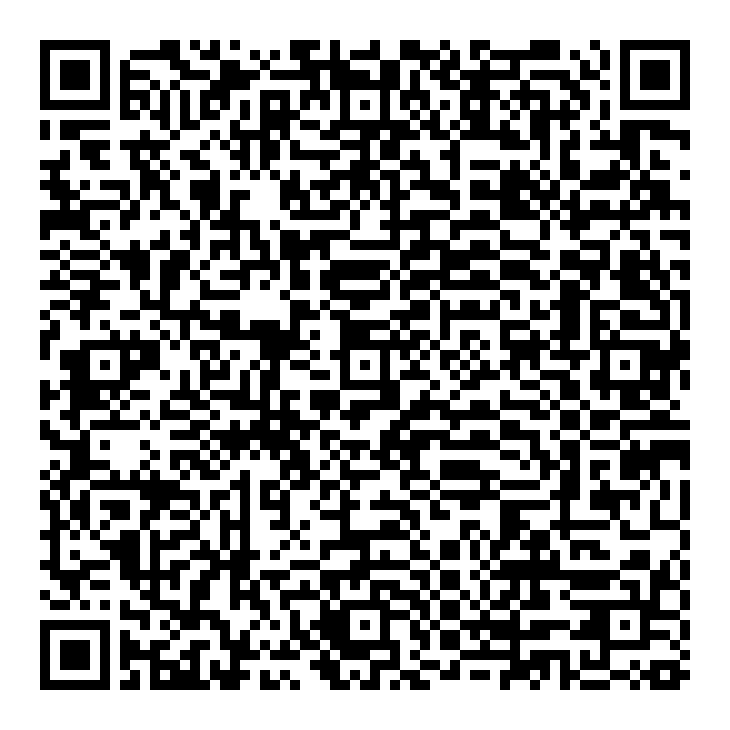C420 | 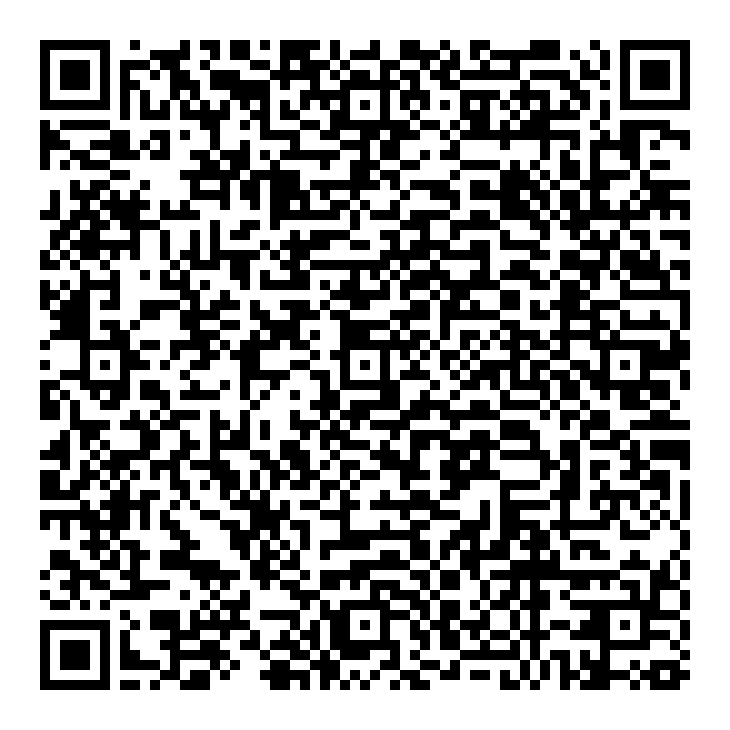C421 | 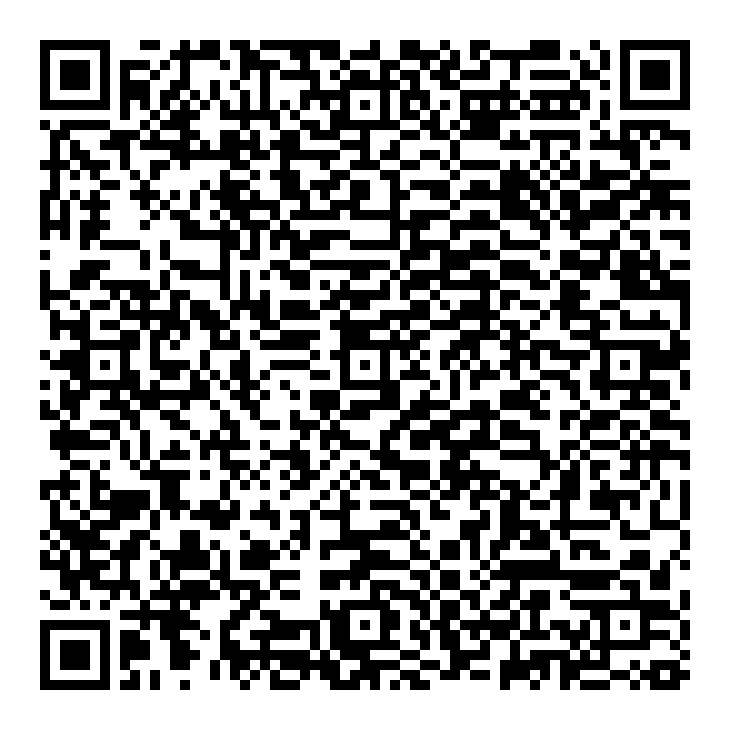C424 | 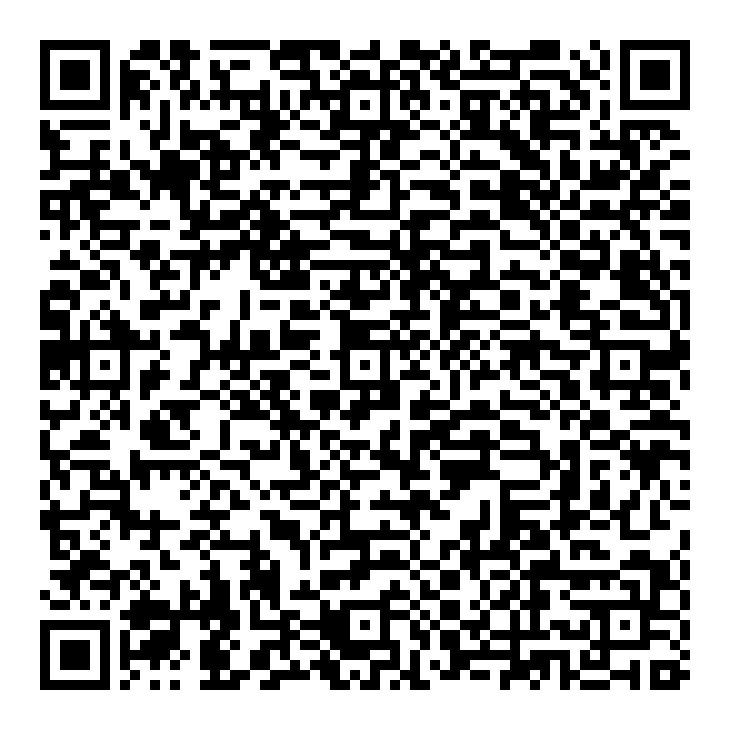C428 |
| 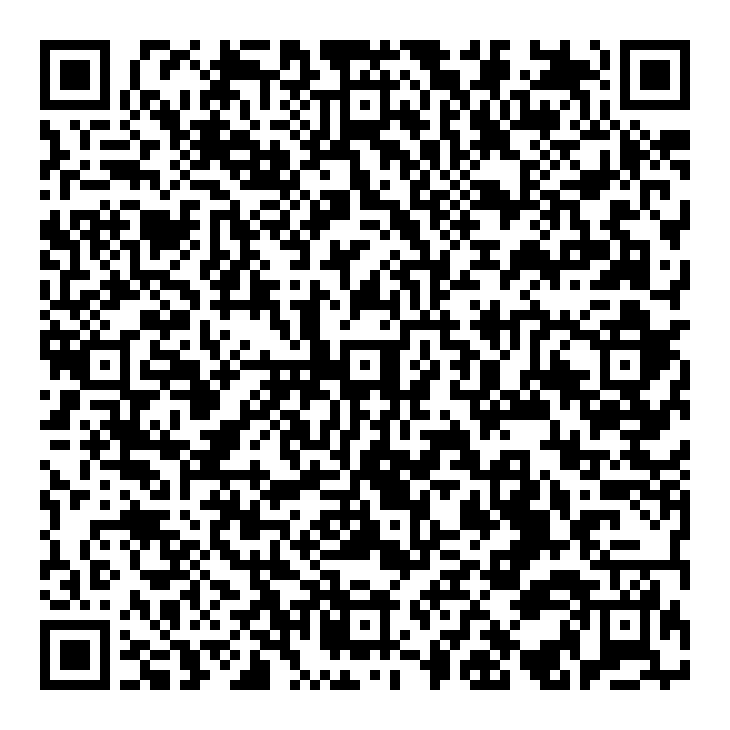C434 | 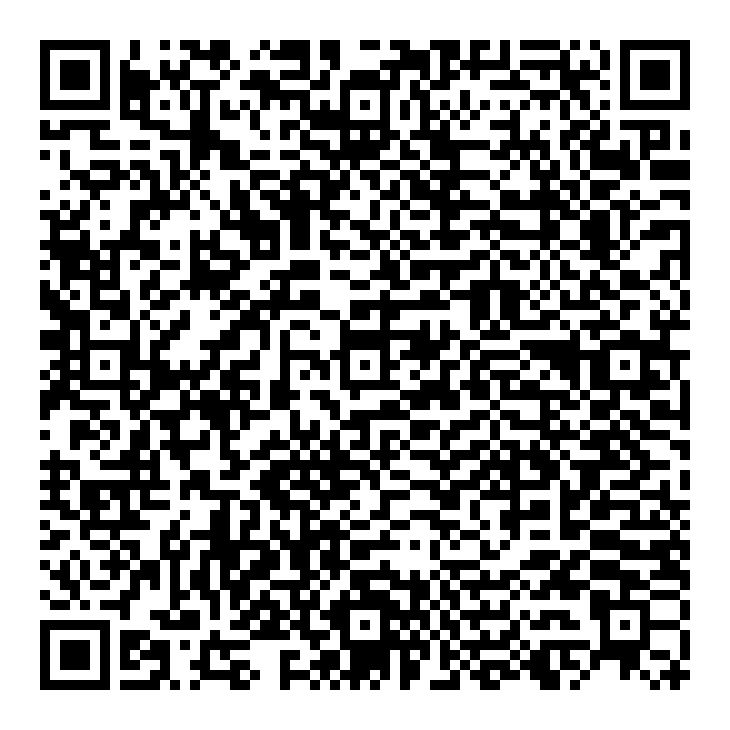S4 | 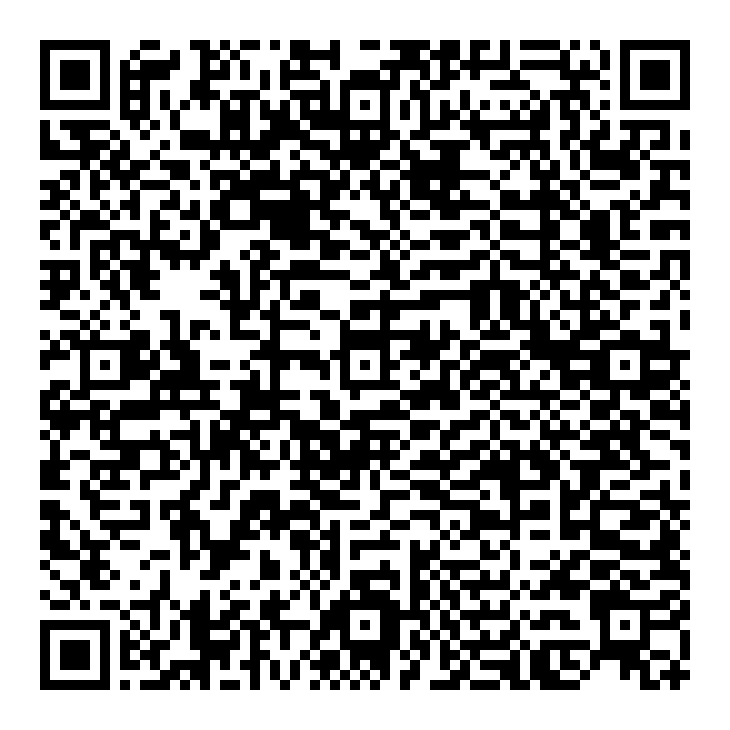S6 | 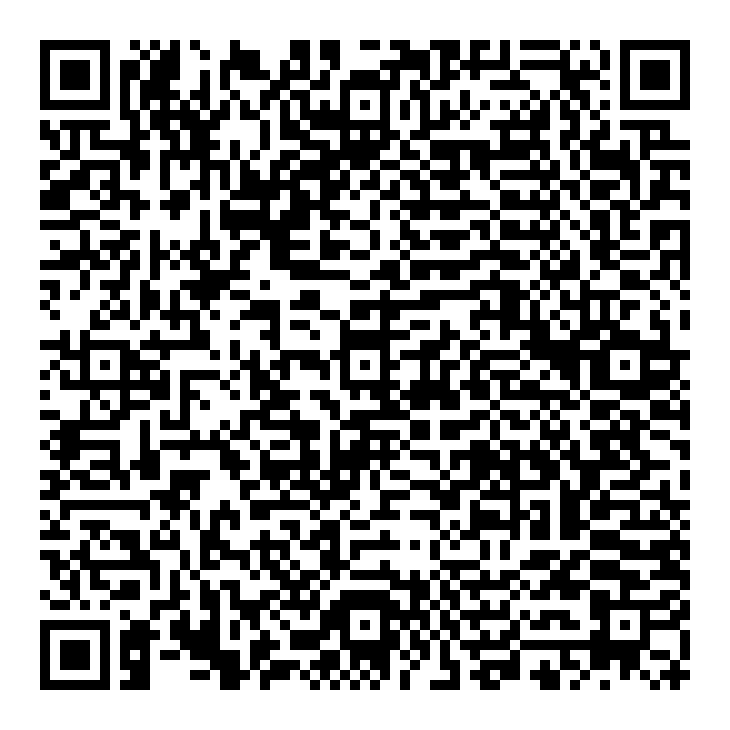S8 |
| 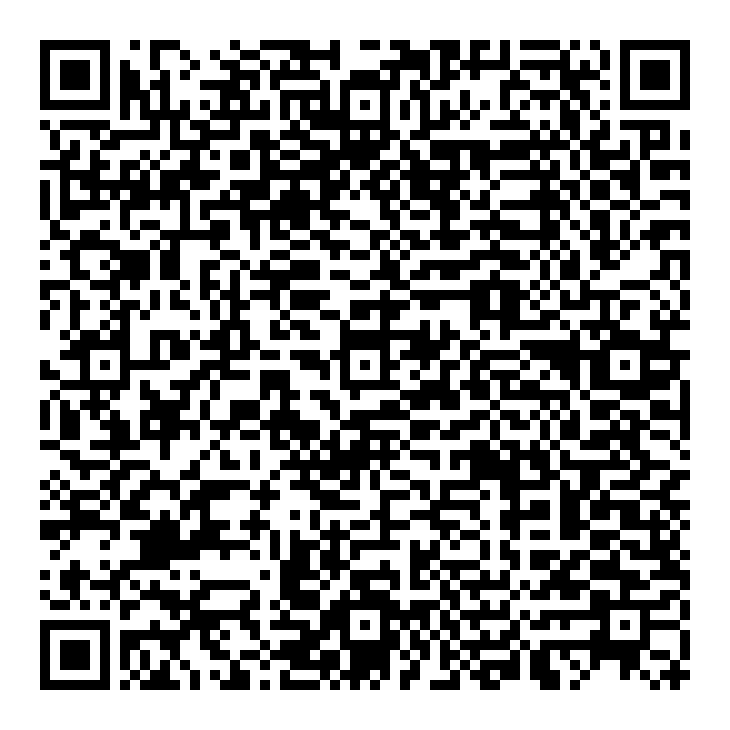S9 | 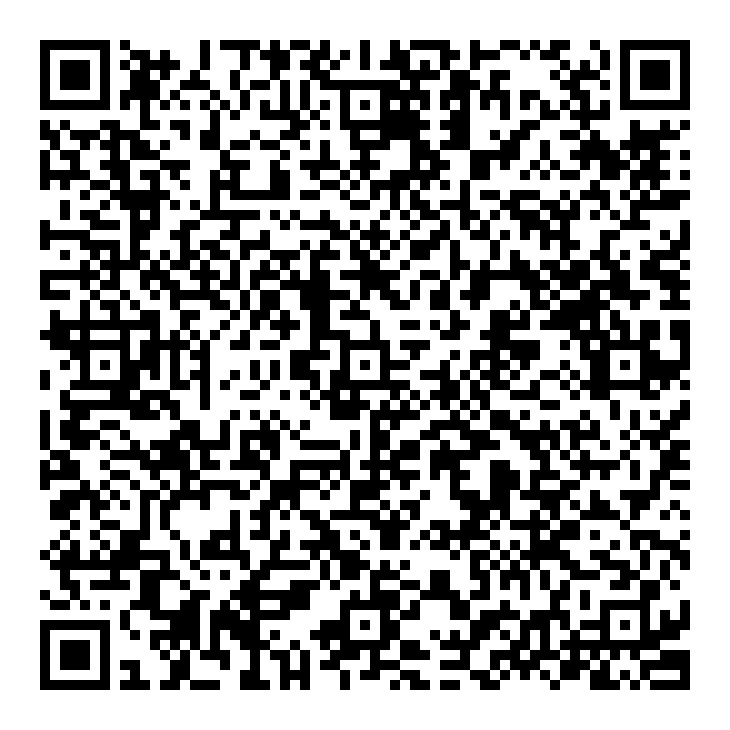S10 | 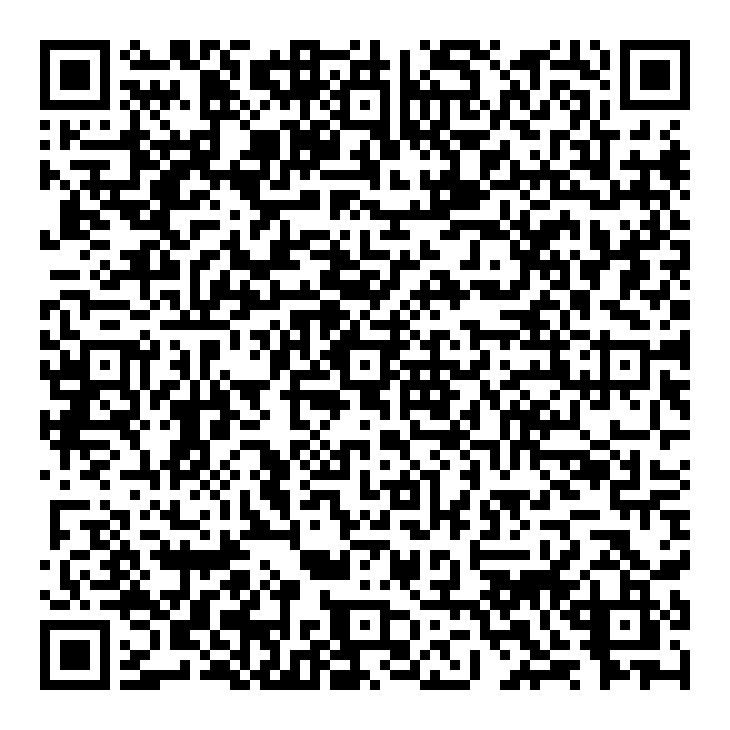S12 | 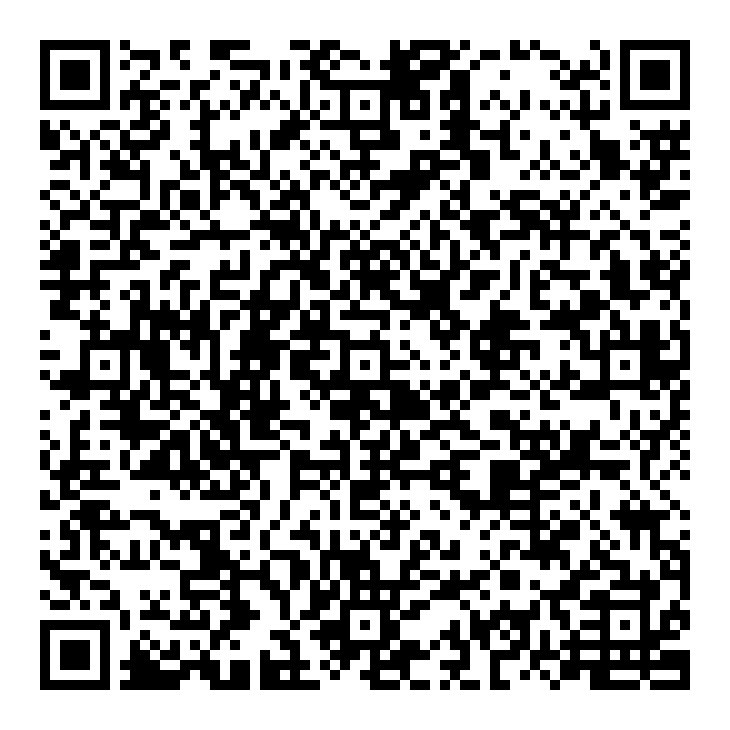S13 |
| 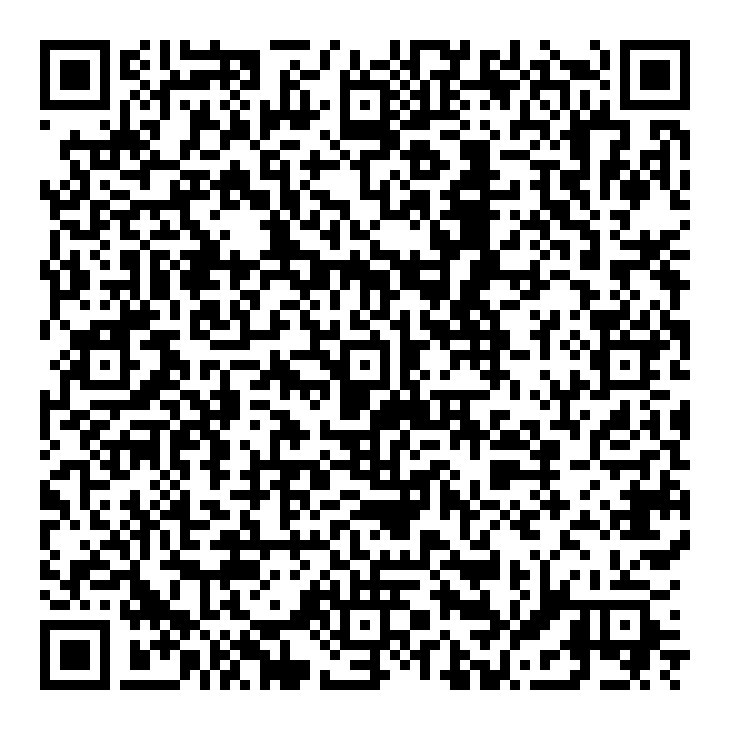S14 | 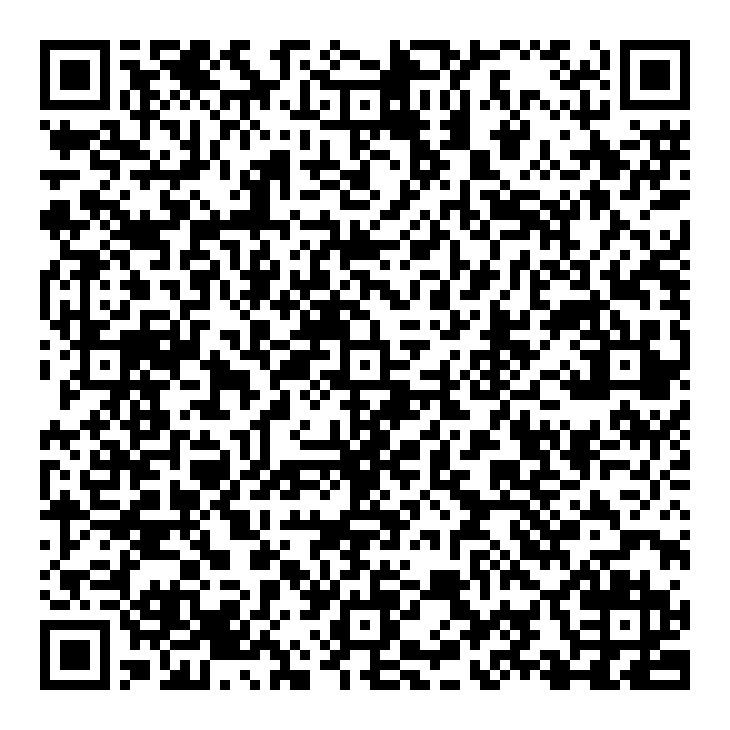S16 | 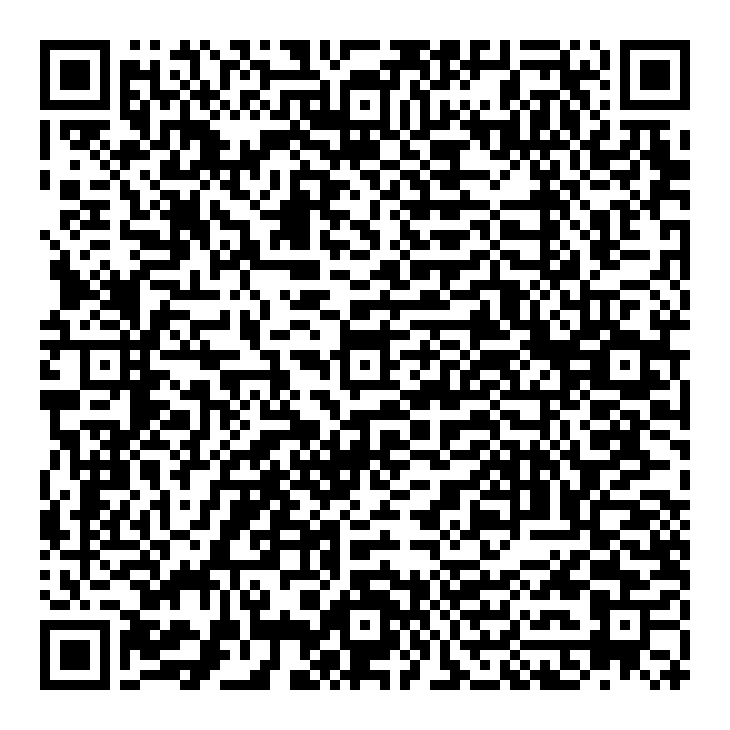W1 | 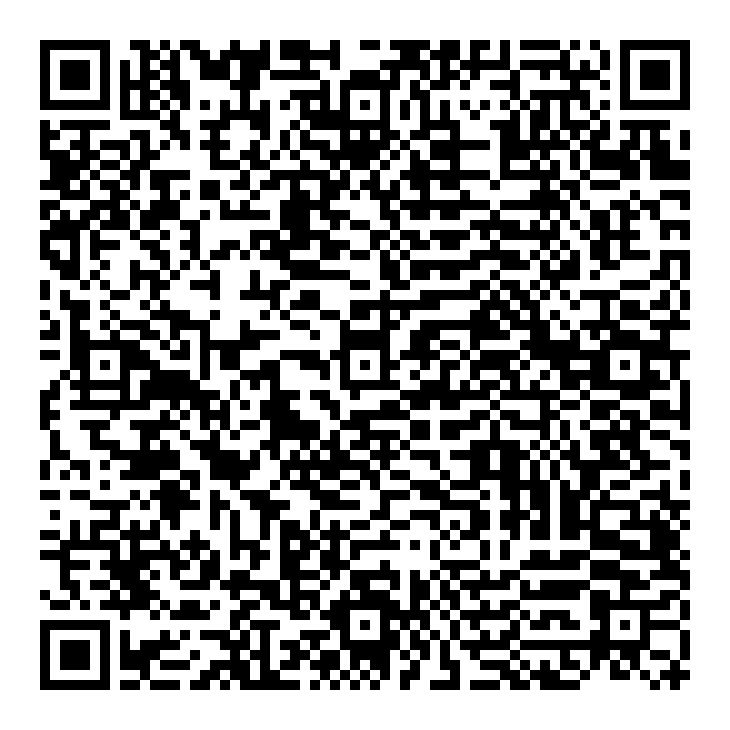W3 |
| 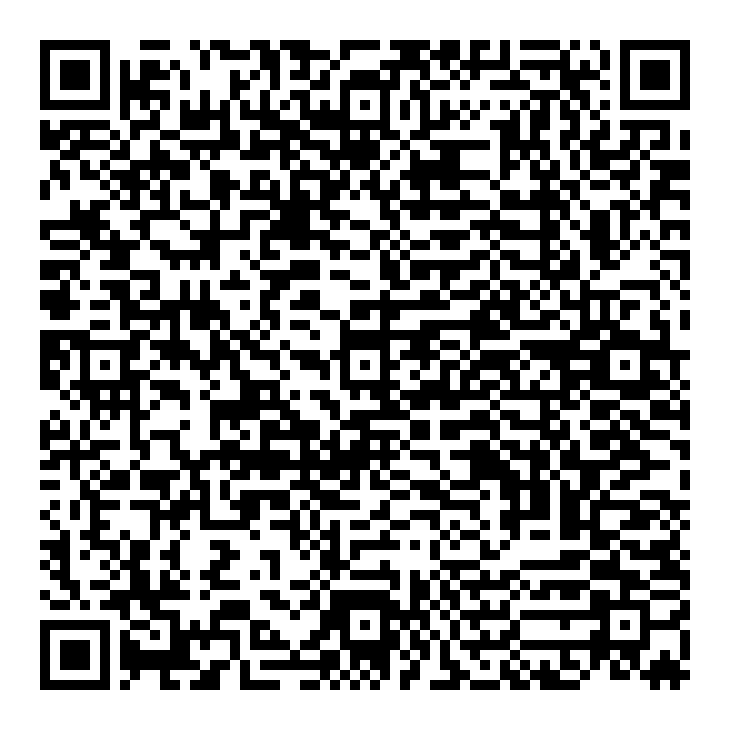W4 | 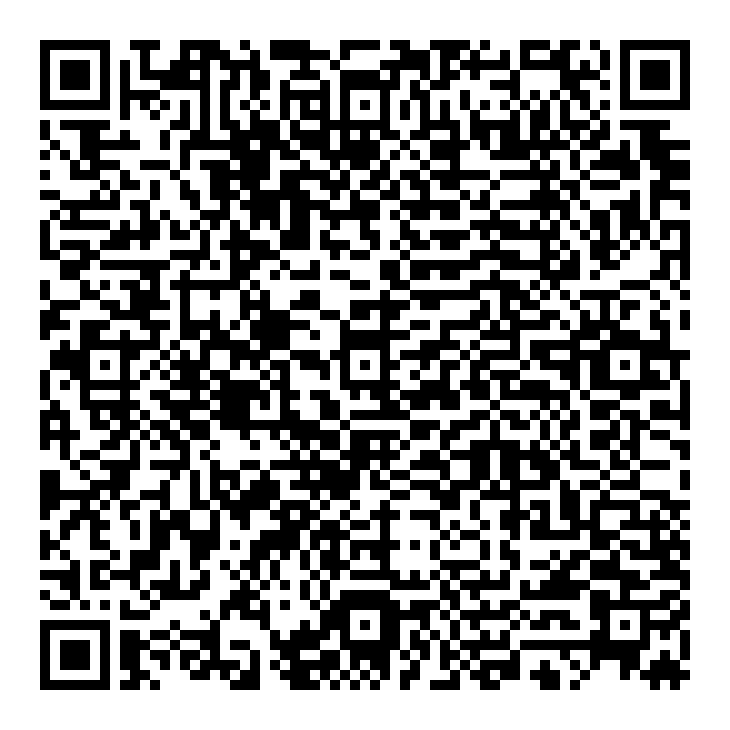W5 | 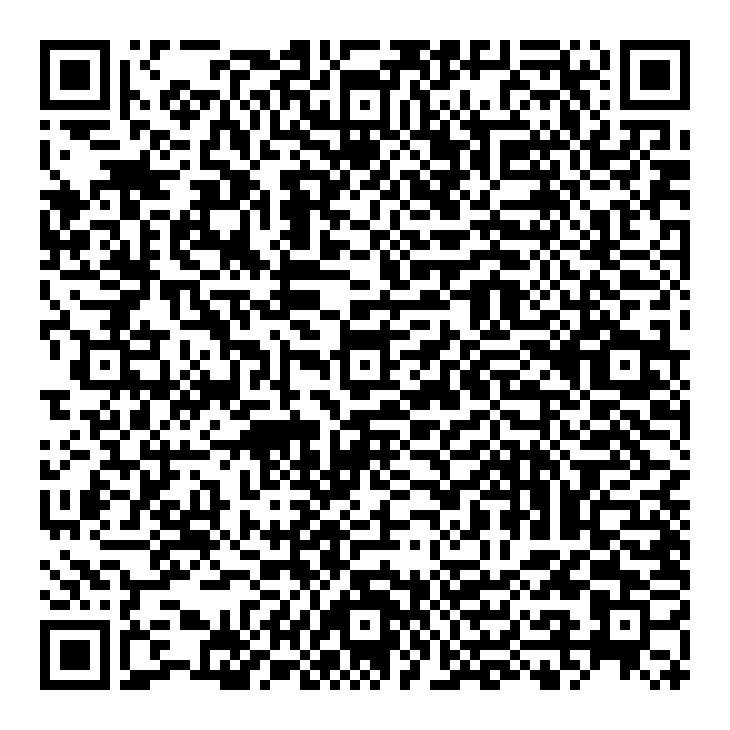W6 | 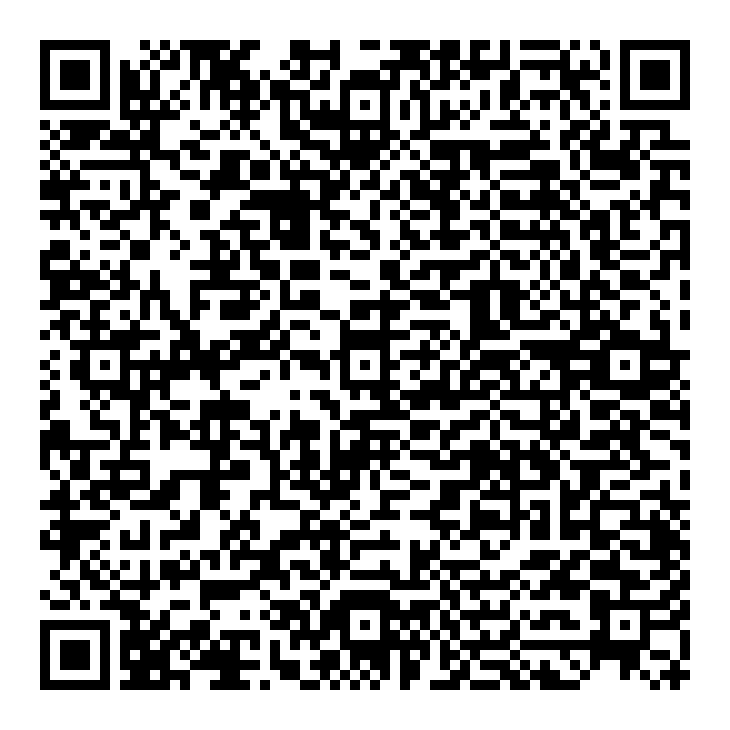W7 |

**Data 1.** The two-dimensional barcode fingerprints of 92 core jujube germplasms

| **46** **natural population samples**  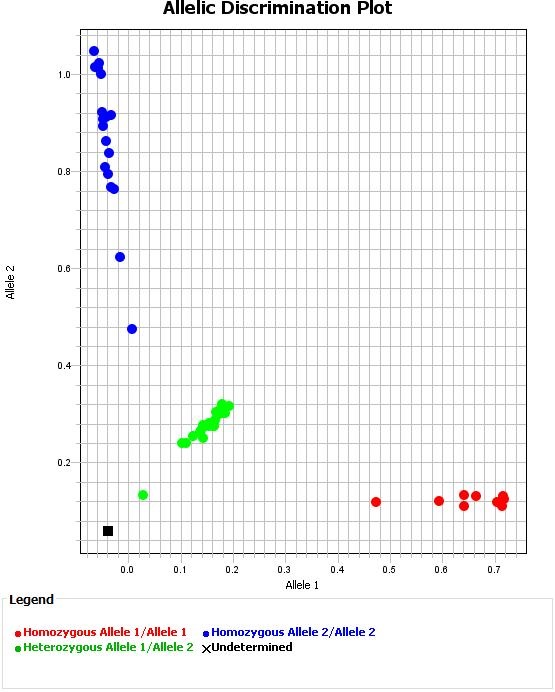  Chr01_748311 | 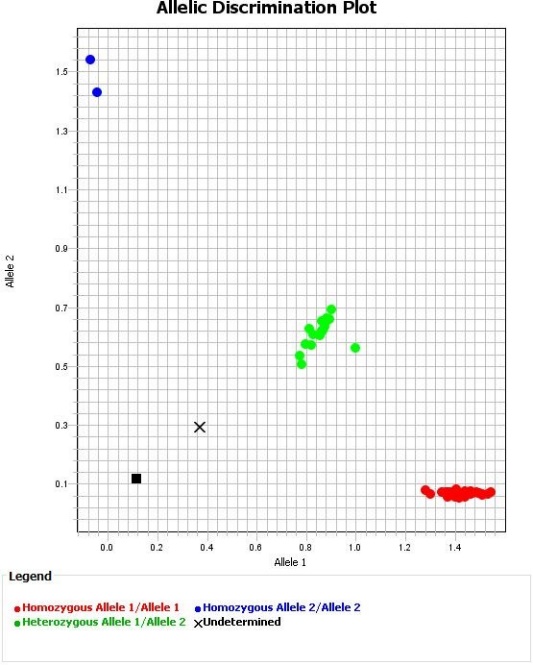  Chr01_15609708 | 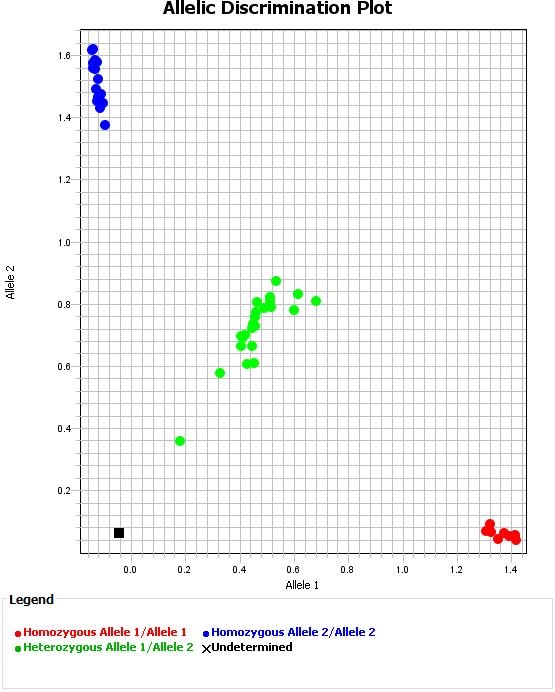  Chr02_891832 |
| --- | --- | --- |
| 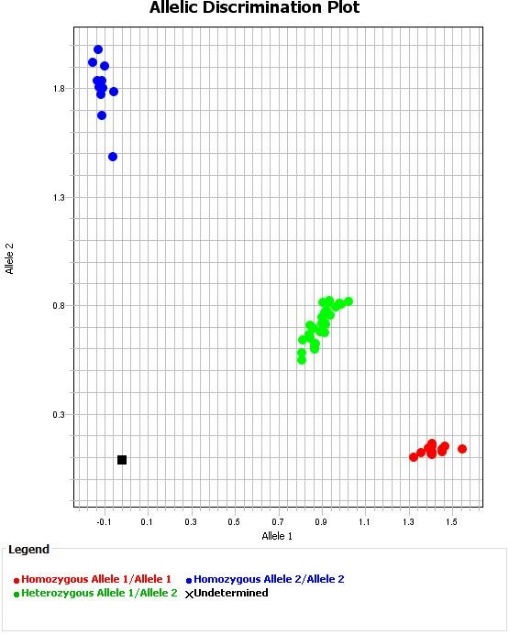  Chr04_1507807 | 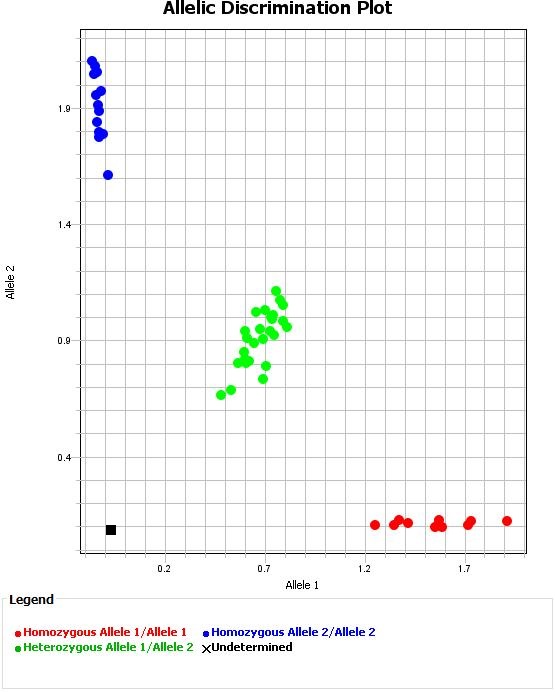  Chr04_2732996 | 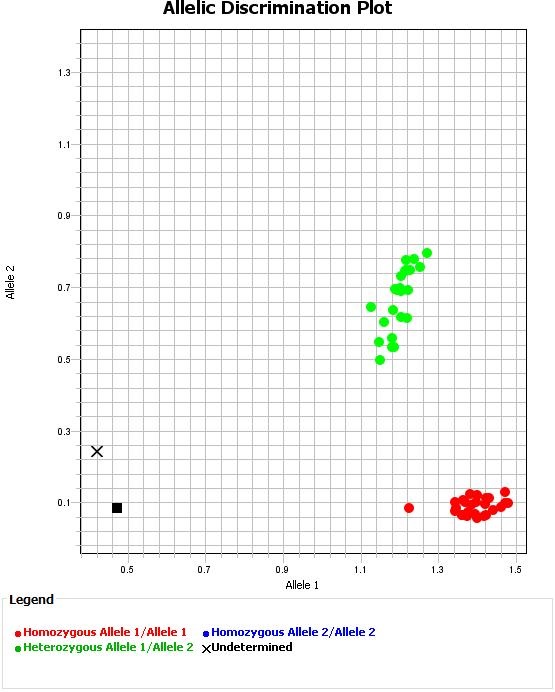  Chr04_19767972 |
| 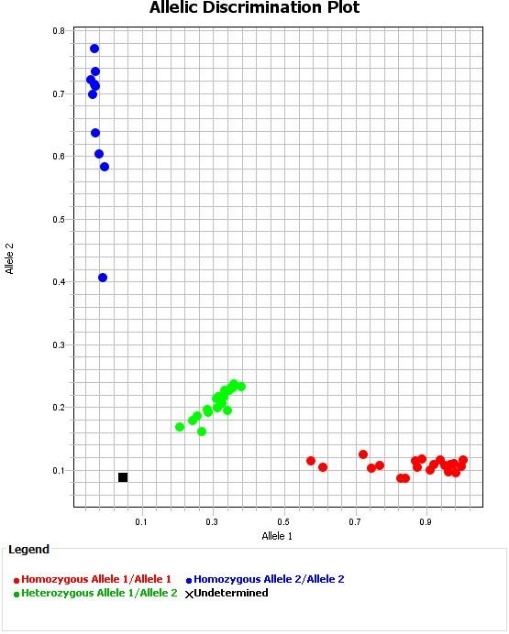  Chr05_840191 | 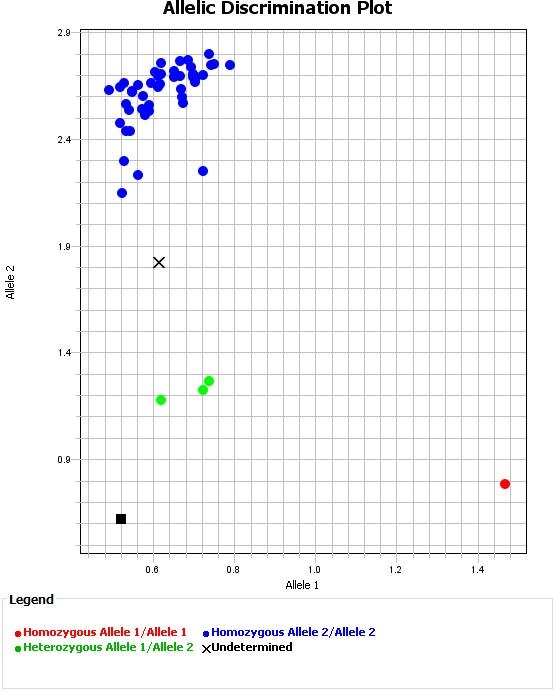  Chr05_14648703 | Chr06_822640 |
| Chr06_1542780 | Chr06_32096649 | Chr07_273819 |
| Chr07_2457030 | Chr07_2539626 | Chr07_12819152 |
| Chr08_1096769 | Chr08_2517264 | Chr08_4052324 |
| Chr09_2609642 | Chr10_2162356 | Chr10_16289987 |
| Chr11_546091 | Chr11_597692 |  |
| **50 hybrid offspring samples** | | |
| Chr01_748311 | Chr01_15609708 | Chr02_891832 |
| Chr04_1507807 | Chr04_2732996 | Chr04_19767972 |
| Chr05_840191 | Chr05_14648703 | Chr06_822640 |
| Chr06_1542780 | Chr06_32096649 | Chr07_273819 |
| Chr07_2457030 | Chr07_2539626 | Chr07_12819152 |
| Chr08_1096769 | Chr08_2517264 | Chr08_4052324 |
| Chr09_2609642 | Chr10_2162356 | Chr10_16289987 |
| Chr11_546091 | Chr11_597692 |  |

**Data 2.** The representative KASP-labelled fluorescence assay results of 46 natural population samples and 50 hybrid offspring samples
